# Selective Aurora A-TPX2 Interaction Inhibitors Have *In Vivo* Efficacy as Targeted Antimitotic Agents

**DOI:** 10.1021/acs.jmedchem.4c01165

**Published:** 2024-08-27

**Authors:** Simon R. Stockwell, Duncan E. Scott, Gerhard Fischer, Estrella Guarino, Timothy P. C. Rooney, Tzu-Shean Feng, Tommaso Moschetti, Rajavel Srinivasan, Esther Alza, Alice Asteian, Claudio Dagostin, Anna Alcaide, Mathieu Rocaboy, Beata Blaszczyk, Alicia Higueruelo, Xuelu Wang, Maxim Rossmann, Trevor R. Perrior, Tom L. Blundell, David R. Spring, Grahame McKenzie, Chris Abell, John Skidmore, Ashok R. Venkitaraman, Marko Hyvönen

**Affiliations:** † Medical Research Council Cancer Unit, University of Cambridge, Cambridge CB2 0XZ, U.K.; ‡ Yusuf Hamied Department of Chemistry, University of Cambridge, Cambridge CB2 1EW, U.K.; § Department of Biochemistry, University of Cambridge, Cambridge CB2 1GA, U.K.

## Abstract

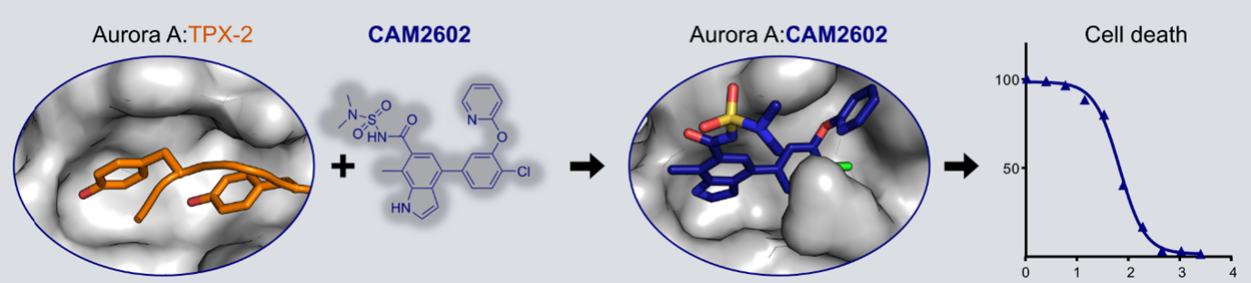

Aurora A kinase, a cell division regulator, is frequently overexpressed in various cancers, provoking genome instability and resistance to antimitotic chemotherapy. Localization and enzymatic activity of Aurora A are regulated by its interaction with the spindle assembly factor TPX2. We have used fragment-based, structure-guided lead discovery to develop small molecule inhibitors of the Aurora A-TPX2 protein–protein interaction (PPI). Our lead compound, **CAM2602**, inhibits Aurora A:TPX2 interaction, binding Aurora A with 19 nM affinity. **CAM2602** exhibits oral bioavailability, causes pharmacodynamic biomarker modulation, and arrests the growth of tumor xenografts. **CAM2602** acts by a novel mechanism compared to ATP-competitive inhibitors and is highly specific to Aurora A over Aurora B. Consistent with our finding that Aurora A overexpression drives taxane resistance, these inhibitors synergize with paclitaxel to suppress the outgrowth of pancreatic cancer cells. Our results provide a blueprint for targeting the Aurora A-TPX2 PPI for cancer therapy and suggest a promising clinical utility for this mode of action.

## Introduction

Aurora A is a serine/threonine protein kinase that plays an important role in controlling early stages of mitosis, including centrosome maturation and separation, mitotic entry, and bipolar spindle formation.^1,2^ Aurora A may be upregulated in cancer cells as a consequence of chromosome rearrangements, aberrant gene expression, or through protein stabilization. Aurora A overexpression is a common feature of several cancers, including ovarian, prostate, pancreas, and breast, and it has been linked to poor treatment outcome.^3−5^ Disruption of the spindle assembly checkpoint due to Aurora A overexpression promotes tumorigenesis via chromosomal instability and aneuploidy.^3,5−7^ Conversely, genomically unstable cancer cells may become critically reliant on Aurora A function.^8,9^ Androgen receptor-positive models of castration-resistant prostate cancer also show significant sensitivity to Aurora A inhibition.^10^ Furthermore, nongenetic elevation of Aurora A levels is reported to drive resistance to current generation EGFR inhibitors in nonsmall cell lung cancer models,^11^ and tumor resistance to taxanes is a further consequence of aberrant expression.^12,13^ Aurora A inhibitors are also increasingly finding use against AML and related leukemias.^14−16^ Consequently, the cancer therapeutic promise of an effective inhibitor of Aurora A is of much interest and the focus of multiple drug discovery studies.^17−19^

Targeting protein for *Xenopus* kinesin-like protein 2 (TPX2) is a spindle assembly factor essential for mitotic spindle organization, maintaining spindle-pole integrity and microtubule nucleation.^20^ Its interaction with Aurora A mediates localization of Aurora A to spindle microtubules,^21^ regulates Aurora A kinase activity by stabilization of the active protein,^22,23^ and protects the activating Thr288 residue in the catalytic domain of Aurora A from the action of PP1 phosphatase.^24,25^ Aurora A and TPX2 are frequently co-overexpressed in tumors;^26^ therefore, the association of Aurora A and TPX2 comprises a novel oncogenic unit that presents a promising target for cancer therapy.^1,22^

**Figure 1:**
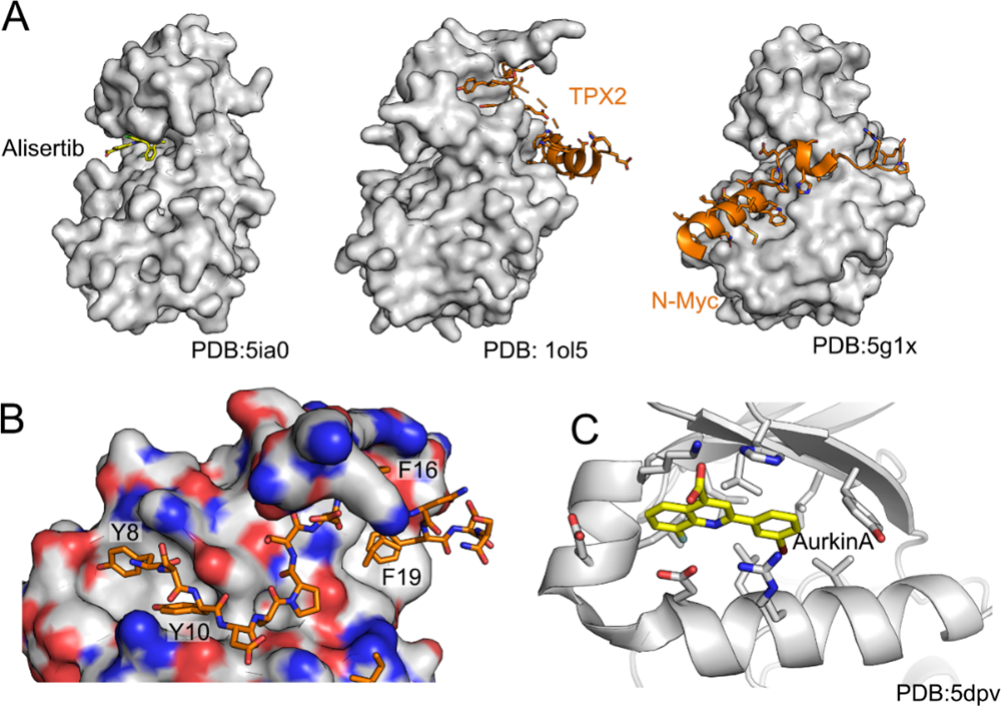
Aurora A interactions and inhibition. (A) Complexes of Aurora A (gray) with different interacting molecules. From left to right, ATP-competitive clinical stage inhibitor alisertib (yellow carbons, PDB: 5ia0(43)), TPX2 peptide (orange carbons, PDB: 1ol5(24)), and N-Myc (orange carbons, PDB: 5g1x(44)). (B) Interaction of the N-terminal part of the TPX2 peptide with Aurora A, with key aromatic residues labeled in the two pockets on the N-lobe. (C) Complex of Aurora A with Aurkin A (yellow carbons, 5dpv^37^), a low-affinity inhibitor of Aurora:TPX2 interaction.

Significant effort has been applied to developing ATP-competitive inhibitors of the Aurora kinases and several have progressed to clinical trials.^17,27,28^ Reported Aurora A inhibitors bind to the highly conserved ATP-binding site of the kinase and consequently exhibit variable selectivity for Aurora A over related kinases, most notably Aurora B and Aurora C.^17,29^ High similarity between Aurora A and Aurora B, especially in their catalytic sites,^30^ makes it challenging to develop highly selective small molecule inhibitors for Aurora A. Alisertib (MLN8237, Figure 1A),^31^ an Aurora A inhibitor in clinical trials, is reported to have a selectivity for Aurora A over Aurora B of approximately 200-fold,^32^ although work using cellular assays to profile and characterize Aurora A inhibitors has indicated an order of magnitude lower specificity.^18,31^ A modest number of early studies have pursued orthogonal approaches to Aurora A inhibition not dependent directly on competition with ATP. Aurora A interaction with N-Myc (Figure 1A) has been disrupted allosterically by ATP-competitive inhibitors, and orthosteric competitors have been identified for the protein–protein interaction (PPI) site with functional binding partner proteins, such as TPX2 (Figure 1A).^33−37^ It is established that kinase inhibitors that target sites other than the ATP pocket can lead to improved selectivity and novel pharmacology.^38,39^ Additionally, therapeutically targeted PPIs are less likely to accommodate mutations without loss of protein function, therefore reducing the potential for emergence of resistance.^40,41^

Although ATP-binding site inhibitors that allosterically disrupt the interaction of Aurora A and N-Myc have demonstrated efficacy in xenografts,^42^ to date, no reported PPI inhibitors of Aurora A-TPX2 have exhibited the potency or pharmacokinetics to be advanced to *in vivo* preclinical models. By targeting the TPX2 binding site unique to Aurora A, we aimed to develop a small molecule inhibitor of Aurora A that was expected to show the therapeutic potential demonstrated by clinical agents such as alisertib and additionally avoid the selectivity issues that typify ATP-competitive molecules. Moreover, by disrupting binding to a scaffolding protein TPX2, we also hope to achieve greater efficacy or new biological effects through different mechanisms of action.

## Results

### Development of Aurora A:TPX2 Interaction Inhibitors

#### Fragment-Based Drug Design

We have pursued a structure-guided fragment-based drug development approach to develop inhibitors of the Aurora A:TPX2 interaction. Previous work from us and others have shown that the key interactions between Aurora A and TPX2 involve residues in the N-terminal half of the TPX2 epitope with mutation of tyrosines 8 or 10 or phenylalanine 19 resulting in significant drop in affinity for Aurora A^34^ (Figure 1B). Also, the previously described Aurora A:TPX2 inhibitor Aurkin A binds to the so-called Tyr pocket, inhibiting this interaction (Figure 1C). This region of the TPX2 interaction does not overlap with where N-Myc binds to Aurora A (Figure 1A).

**Figure 2:**
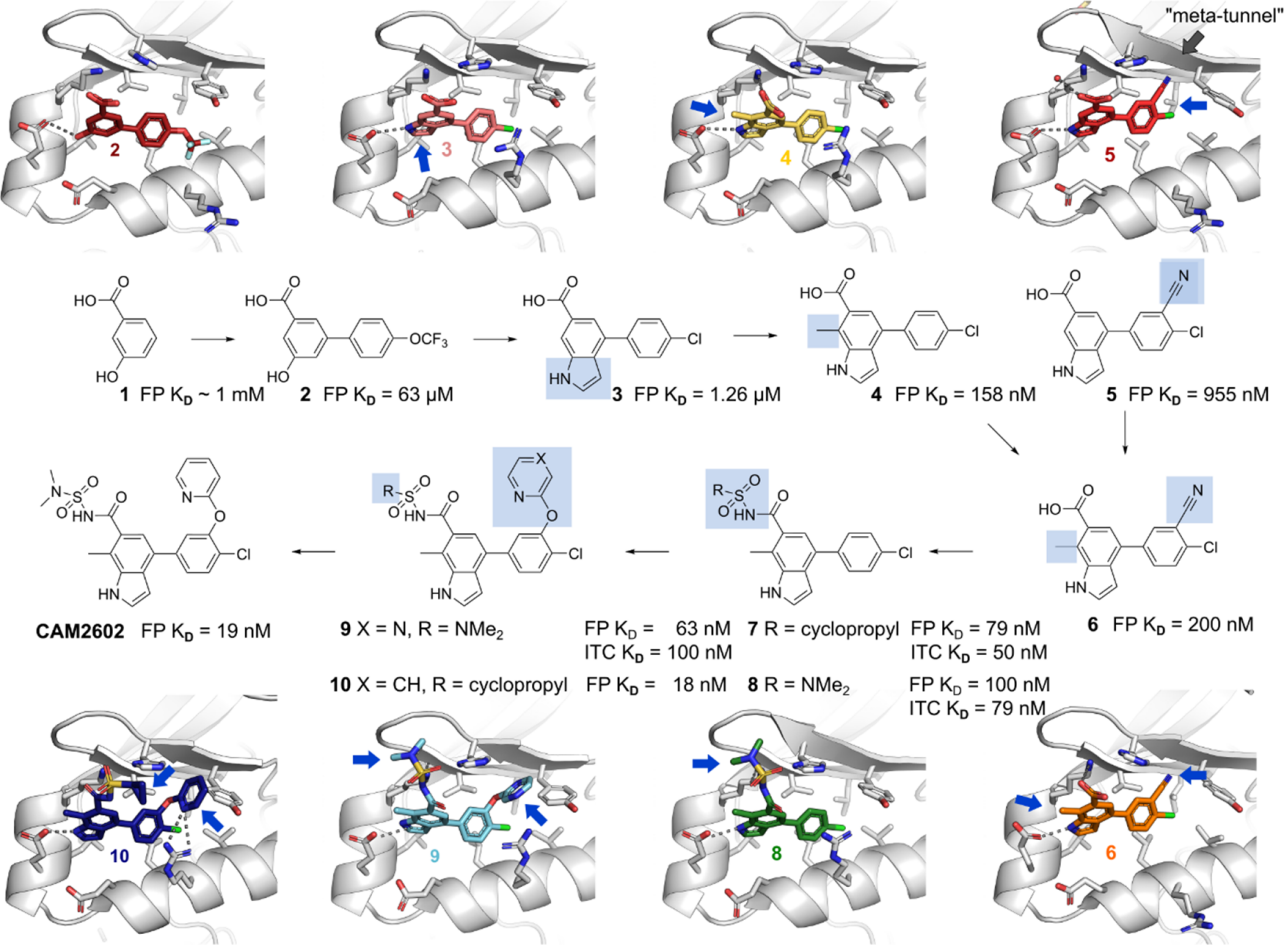
Aurora A:TPX2 interaction inhibitor design. Overview of the fragment-based development of **CAM2602** to inhibit the Aurora A:TPX2 protein–protein interaction. Chemical structures are shown for compounds **1–10** and **CAM2602**. Crystal structures of compounds **2 (PDB:8C1M)**, **3 (8C15)**, **4 (8C1D)**, **5 (8C1E)**, **6 (8C1F)**, **8 (8C1H)**, **9 (8C14)**, and **10 (8C1I)** in complex with Aurora A are shown next to the chemical structures. The blue boxes on the chemical structures and corresponding blue arrows on the crystal structures highlight the key change(s) at each step. The “meta-tunnel” is marked in the structure of **5**. The *K*_D_ values are obtained from a competitive FP assay or a direct ITC measurement.

Our aim was to develop potent inhibitors binding at this Tyr pocket with properties that would enable *in vivo* evaluation of this approach to Aurora A inhibition. We started this process by screening a library of 600 fragments by a thermal shift assay in the presence of an ATP-site binding inhibitor to focus fragment binding to sites other than the ATP site. Thermal shift hits were progressed into ligand-based NMR experiments, where a number of these such as 3-hydroxybenzoic acid (**1**) were shown to bind Aurora A and could be displaced by a TPX2 peptide fragment (amino acids 7–22) but not by a tight-binding ATP-site ligand. We established a competitive fluorescence polarization (FP) assay with a longer, fluorescently labeled TPX2 peptide to mimic the native-like interaction, but these NMR hits had no measurable activity in this assay. Moreover, we could not observe electron density for the fragments in X-ray crystallographic soaks. A focused iteration of chemical elaboration of these hits yielded further fragments that maintained the desired competition profile in ligand-based NMR experiments, possessed activity in the FP assay, showing *K*_D_ values of around 1 mM, and were confirmed to bind to Aurora A by isothermal titration calorimetry (ITC). Crucially, we were also able to obtain crystal structures of some of these hits in complex with the Aurora A protein, enabling a structure-based drug design. A representative of such fragment is compound **2** (Figure 2), a biphenyl molecule bearing a carboxylic acid and a phenol group on one ring and a lipophilic trifluoromethoxy group on the other. Compound **2** has a *K*_D_ of 63 μM as measured by our competitive FP assay and a *K*_D_ of 145 μM as determined by ITC. The binding of **2** to Aurora A, as determined by X-ray crystallography, alongside some key structural motifs showing both the ATP site and TPX2 peptide binding sites, is highlighted in Figure 2. Our ligand-observed Carr-Purcell-Meiboom-Gill (CPMG) NMR experiments and FP studies showed that these fragments are competitive with the TPX2 peptide (Figure S1), and X-ray crystallography revealed that the hit fragments bind to part of the TPX2 binding site (Figure 2), normally occupied by the Tyr8 and Tyr10 of TPX2.

**Figure 3:**
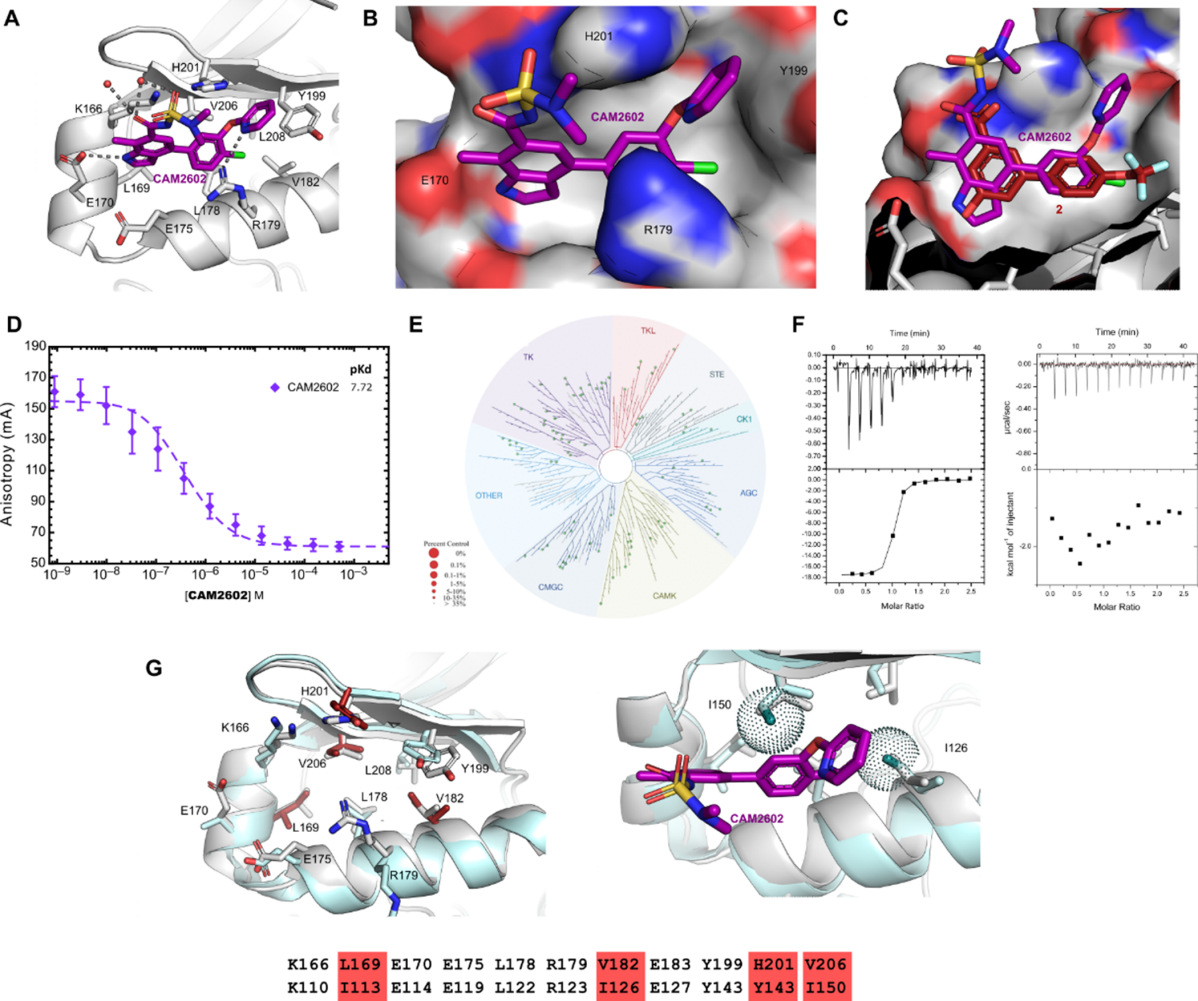
**CAM2602** characterization. (A) X-ray crystal structure of **CAM2602** bound to Aurora A is shown (purple carbons; PDB: 8C1K) along with key interactions in the Tyr pocket (B) View from above the Tyr pocket with Aurora A as a molecular surface. (C) Overlay of compound **2** and **CAM2602** shows remarkable preservation of the binding pose across the inhibitor development series. (D) Competition fluorescence polarization assay of **CAM2602** competing with the TPX2 peptide. (E) Kinase panel results were obtained using compound **9**. Red spheres indicate cross-reactivity with kinases in the phylogenetic tree with no observed reactivity for compound **9** in the panel of 97 human kinases; details in Figure S3. (F) Isothermal titration calorimetry titration of compound **8** to Aurora A (left) and to Aurora B (right). (G) Conservation of residues in the Tyr pocket between Aurora A (light gray, PDB: 8C1K) and Aurora B (pale blue, PDB: 4AF3) with residues lining the Tyr pocket shown as sticks and nonconserved residues in Aurora B colored red. The same residues are shown below the figure with red background for nonconserved ones. On the right is a zoomed-in view of **CAM2602** binding to Aurora A, overlaid with the Aurora B structure (pale blue). The surfaces of additional methyl groups in Ile126 and Ile150 are displayed with surface dots, showing their proximity to **CAM2602**.

Through a further iterative development of the inhibitors utilizing the crystal structure-guided drug design and biophysics (FP and ITC; Figures S1, S2, and Table S3), we improved the affinity of our weak, millimolar fragments by over 10,000-fold to generate the lead compound **CAM2602.** An early modification was to change the phenol group of **2** into indole while replacing the trifluoromethoxy with a smaller chlorine to give **3**, which improved the *K*_D_ to 1.26 μM (Figure 2). The indole-aryl core of the molecule lays in a hydrophobic pocket assembled from Leu169, Leu178, Val182, Val206, and the side chain of Lys166. The indole nitrogen proton forms a hydrogen bond with the side chain of Glu170 thus mimicking the phenol of Tyr8 of TPX2. The carboxylic acid group was observed to interact with Lys166 and His201. Furthermore, the electron density supported it being twisted from the plane of the indole ring in order to form a salt-bridge with Aurora A (Figure 2). Our analysis of ligands in PDB and CSD^45^ databases shows that carboxylic acids are more commonly in-plane with the aromatic ring (data not shown) and presumably this twisting incurs an energetic penalty upon binding. To minimize the loss of binding energy and to stabilize the torsional twist in the ground state, we introduced a methyl group in the position C-7 of the indole system to give **4**, which improved the K_D_ to 158 nM. We found that introduction of a *meta* nitrile group in the *para*-chloro ring led to a further modest improvement in potency and the crystal structure of Aurora A in complex with **5** revealed that the induced movement of Tyr199 generated a small pocket for the nitrile group between Tyr199 and His201 (the “*meta*-channel”). Combining the modifications in compounds **4** and **5** to give **6** resulted in FP activity similar to that of **4** and good cell permeability, permitting us to use **6** as a tool compound, particularly for early cell-based experiments. However, the potential utility of **6***in vivo* is primarily hampered by poor hepatocyte stability, which was improved significantly through the introduction of isosteric replacements for the carboxylic acid, particularly acyl sulfonamide and sulfamide in compounds **7** and **8**, respectively. In addition, the *meta-*channel between Tyr199 and His201 could be further exploited by the replacement of nitrile with heteroaryl ether, to give compounds **9**, **10,** and the lead compound **CAM2602** (Figures 2 and 3). **CAM2602** engages with the Tyr pocket through hydrophobic interactions at the bottom of the pocket and with polar interactions further outside. The indole NH hydrogen bonds with the Glu170 side chain, and the acyl sulfonamide stacks against His201 and Lys166. The pyridine ring in the *meta* position pushes Tyr199 sideways, creating a channel between His201 and Tyr199 and forming a T-stacked aromatic interaction with the latter. Finally, Arg179 latches on to the central aromatic ring, with **CAM2602** bound to a well-defined pocket which is partly induced by the binding of the inhibitor (Figure 2A,B).

Our lead series maintains the acidic group present in fragment **2**, either as a carboxylic acid, an acyl sulfonamide, or acyl sulfamide, while the phenol has been replaced with an NH in the form of an indole. An overlay of the crystal structures of early hit **2** with **CAM2602** bound to Aurora A reveals a remarkable overlap of the core biaryl scaffold in the two compounds (Figure 3C). **CAM2602** displaces TPX2 from Aurora A with a *K*_D_ of 19 nM and a ligand efficiency of 0.33 (Figure 3D).

#### Kinase Selectivity

We thoroughly evaluated the selectivity of our Aurora A:TPX2 inhibitors early on in the program. First, we tested **9** against 97 protein kinases in the DiscoverX KINOMEscan and failed to observe any detectable activity against these kinases at 10 μM, as expected from a non-ATP-site inhibitor (Figure 3E).

Given our inhibitors bound to a PPI site, we hypothesized that they would show high selectivity for Aurora A over other kinases including Aurora B. Achieving selectivity over Aurora B has been recognized as a desirable feature of new drugs, but has thus far been challenging to achieve, due to the high sequence similarity (>70% identity) between the two kinase domains^2,24,46,47^ and the presence of a site that is analogous to the TPX2 binding site that, in the case of Aurora B, binds to the protein INCENP. To ensure our molecules did not bind to Aurora B, we measured binding of lead series representatives **7**, **8**, and **9** to both Aurora A and B by ITC. As expected, a good correlation is observed between the *K*_D_ of our inhibitors for Aurora A measured by competitive FP experiments and that from direct binding to Aurora A by ITC. Additionally, we observed an approximate 300-fold selectivity for Aurora A over Aurora B for compounds **7** and **8** (Figure S2). With the introduction of a *meta*-ether substituent in **9**, the compound’s potency against Aurora B was too weak to be measured—indicating greater than 1000-fold selectivity for Aurora A (Figure 3F). The specificity of **9** for Aurora A over Aurora B is at least as great as the best compounds reported previously.^18,48^

The determinants of Aurora A vs B selectivity could be rationalized from our crystallographic data. Although many key residues that interact with their respective ligands are conserved, the shape of the base of the pocket is altered by three changes. In particular, His201, which in Aurora A is an important side chain that forms a π-stack with the heterocyclic ethers and potentially participates in a charged interaction with the sulfonamide moiety in our lead compounds, is a tyrosine residue in Aurora B (Tyr143). Val182 and Val206 of Aurora A are both replaced by isoleucines in Aurora B, with the extra methyl groups making the Aurora B pocket somewhat smaller (Figure 3G).

Potential toxicity of **CAM2602** was evaluated in protein-based Cerep panels, cellular toxicity assays, and peripheral blood mononuclear cell (PBMC) assays. High-content cell toxicology of compound **7**, up to 40 μM in HepG2 cells, indicates that there were no measurable effects on cell growth, nuclear size, DNA structure, cell membrane permeability, mitochondrial mass, mitochondrial membrane potential, or cytochrome c release (Table S1). The lead compound **CAM2602** exhibits only one off-target activity in the Cerep screen, inhibiting the binding of an agonist radioligand to human adenosine 3 (A3) GPCR by 55% at 10 μM. **CAM2602** does not inhibit hERG or a panel of cytochrome P450 enzymes at 25 μM (Table S3). Full ADMET properties of **CAM2602** are shown in Table S3.

### Mechanistic Characterization of the Aurora A:TPX2 Inhibitors

#### Target Engagement in Cells Induces Aurora A Mislocalization

**Figure 4:**
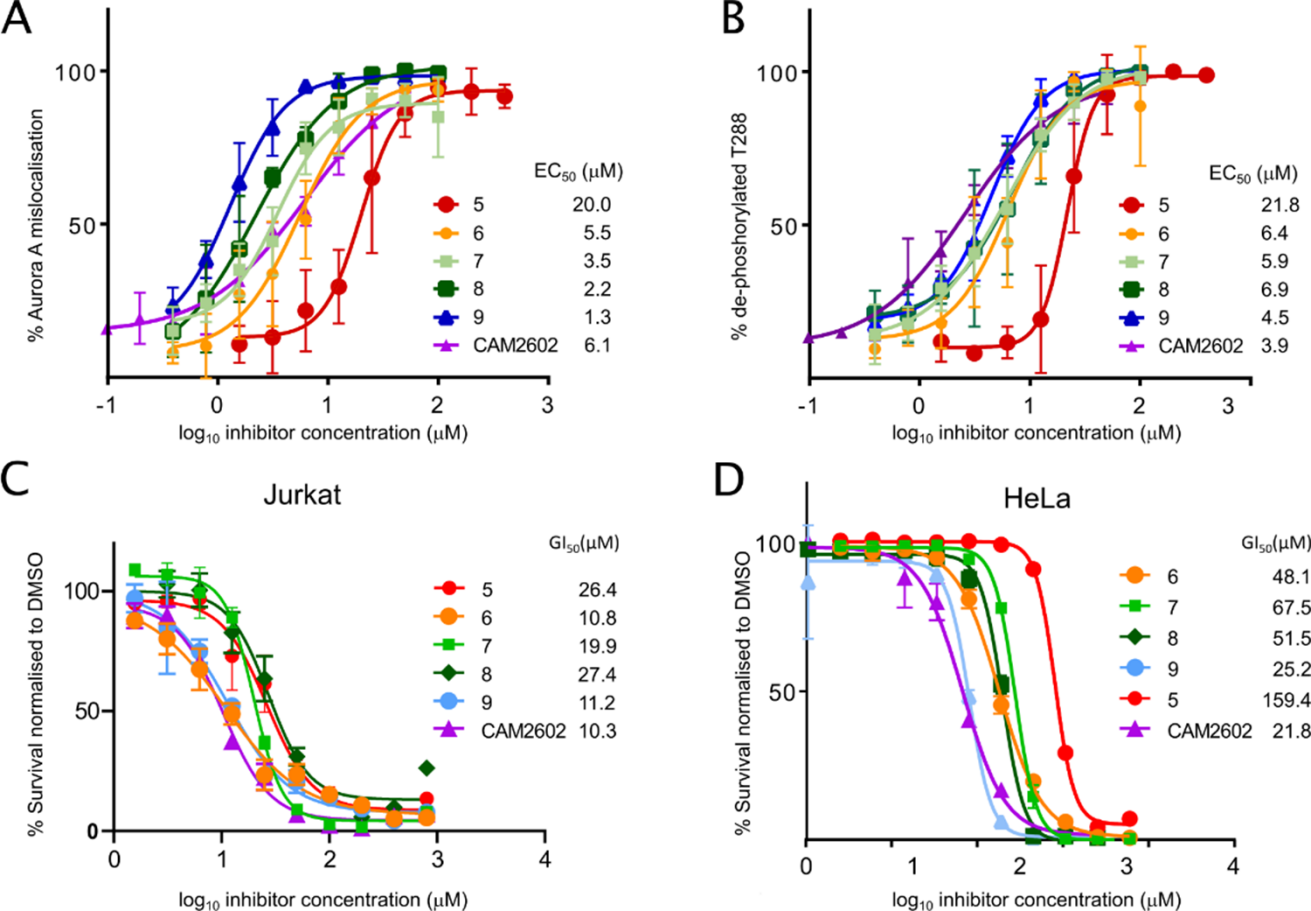
Cellular efficacy of the **CAM2602** series. (A) High-content microscopy assays to evaluate mislocalization of Aurora A from the mitotic spindle or loss of P-Thr288 Aurora A in mitotic nuclei when treated with the inhibitor. HeLa cells were treated with titrations of the indicated compounds for 2 h before being fixed, stained for Aurora A, and analyzed using high-content microscopy to determine the percentage of observed mitotic cells at each concentration with spindle-displaced Aurora A (mislocalization). The indicated EC_50_ values for each compound were calculated from the plots of assay scores against the compound concentration. (B) As in A but stained for dephosphorylated Thr288 Aurora A. (C) Viability assays in Jurkat cells. Jurkat cells were cultured for 72 h with titrations of the indicated compounds. Viability assays were performed following the treatment period and the data normalized to the vehicle-treated controls. GI_50_ values were calculated from plots of the viability assay data. (D) Viability in HeLa cells, determined similarly to (C).

Previous reports have shown that Aurora A is recruited to the mitotic spindle through its protein–protein interaction with TPX2.^21,22^ We have previously reported a high-content screening assay in which we can detect the displacement of Aurora A from the spindle in mitotic cells.^37^ Here, we used this assay to provide a measure of cellular target engagement for our key compounds (Figure 4). In parallel, we performed a related high-content assay measuring loss of the activating phosphorylation at threonine 288 (P-Thr288) on Aurora A. In agreement with previous data,^37^ the EC_50_ values in these two assays were well-correlated (Figure 4A,B).

An acute cellular consequence of inhibiting the mitotic function of Aurora A is the appearance of spindle abnormalities in those cells undergoing mitotic division.^49,50^ Driven by deregulation of centrosome maturation and spindle-pole forces, the abnormalities can be broadly characterized as including loss of spindle bipolarity and/or misalignment of the condensed chromosomes at the metaphase spindle; observations of these phenotypes have been used in preclinical and clinical studies employing ATP-competitive Aurora A inhibitors.^32,51,52^ Treatment of HeLa cells with compound **6** for 6 h resulted in significant increase in misaligned or trailing chromosomes based on immunofluorescence microscopic analysis of chromatin DNA, Aurora A, and α-tubulin (Figure S4A,B).

#### Impact on Viability in Dividing Cancer Cells

Blocking the PPI between Aurora A and TPX2 is predicted to disrupt Aurora A function in dividing cells^20^ leading to defects in spindle assembly, transient activation of the spindle assembly checkpoints, and eventual apoptosis in a postmitotic G1 arrest.^53^ Actively cycling cells experiencing Aurora A inhibition are therefore expected to exhibit an eventual loss of viability due to prolonged disruption of Aurora A function. The compounds were titrated in the growth assay to estimate their cytotoxic impact against either Jurkat acute T cell leukemia cells or HeLa cervical adenocarcinoma. Jurkat cells have been used widely for the preclinical testing and validation of compounds that target enzymes like Aurora A regulating cell cycle arrest and progression.^54^ They exhibit sensitivity to such inhibitors in *ex vivo* culture models and also as xenografted tumors in immunocompromised murine strains.^55^

In general, we observed lower GI_50_s for our compounds in Jurkat cells compared to HeLa cells (Figure 4C,D). To explore the potential therapeutic window for our compounds in dividing cancer cells versus normal tissues, we made use of peripheral blood mononuclear cells (PBMCs). PBMCs are viable in tissue culture conditions, but do not cycle in the absence of a lymphocytic stimulus such as anti-CD3/CD28.^56,57^ Noncycling cells should not require active Aurora A, so assessing cell viability in the PBMCs may serve an indirect measure of potential off-target toxicity. We observed that most of the compounds with cell activity in HeLa and Jurkat cell viability experiments had no impact on the noncycling PBMCs when applied at less than 200 μM, which was an order of magnitude greater than the typical GI_50_ values seen in the equivalent Jurkat cell data (Figure S5). As a control, the PBMCs were also treated with an ATP-competitive Aurora A inhibitor, alisertib, which also demonstrated no toxicity in the PBMCs. Treatment with staurosporine, a nonselective kinase inhibitor that exhibits promiscuous cytotoxicity, resulted in dose-related killing PBMCs, confirming that the assay was capable of reporting nonspecific cell-killing effects.

#### Biomarkers of Aurora A-TPX2 Disruption

Phosphorylation of serine 10 on histone H3 (PH3) has been used as an indicator of mechanistic target engagement for ATP-competitive Aurora A inhibitor alisertib.^32,58−60^ Aurora A inhibition produces a delayed G2/M transition-driving accumulation of PH3 through the activity of Aurora B.^61,62^ We treated Jurkat cells with either an early lead compound (**7**), alisertib, or a vehicle control and followed PH3 levels over time by Western blotting. Accumulation of PH3 in Jurkat lysates was observed from 16 h following treatment with both alisertib and compound **7** (Figure S6A).

**Figure 5:**
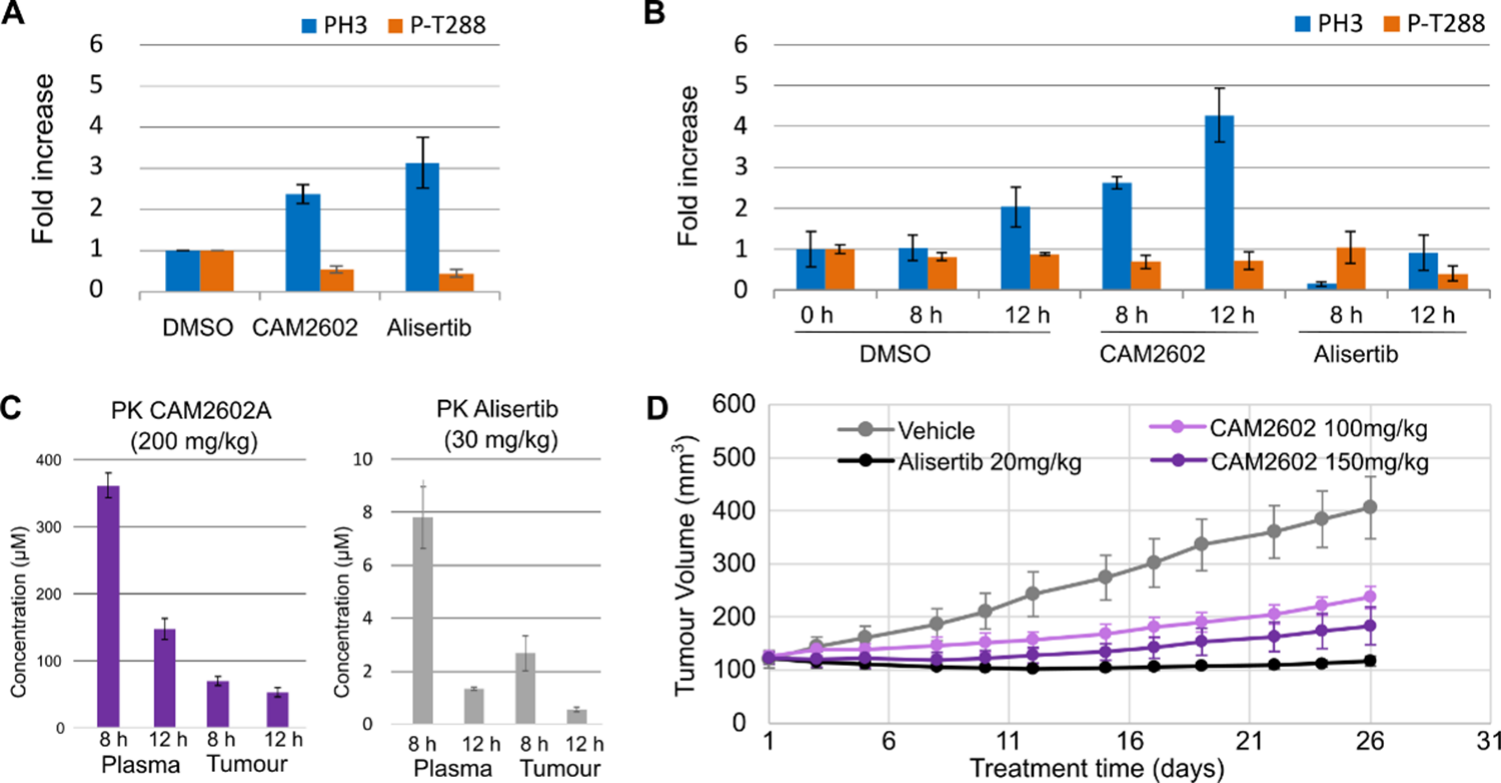
*In vitro* and *in vivo* characterization of **CAM2602**. (A) Jurkat cells were treated for 8 h with 20 μM **CAM2602** or 14 nM alisertib and analyzed by flow cytometry for PH3-positive cells relative to vehicle controls. PH3-positive cells from each sample were assessed for a loss of P-Thr288 positivity. (B) Female NSG mice bearing solid Jurkat tumors (subcutaneous implantation, rear dorsum) were administered a single oral dose of either **CAM2602** or vehicle. Tumor cells from 0, 8, or 12 h of treatment were analyzed by flow cytometry similarly to *in vitro* samples in panel A. (C) Pharmacokinetic analysis of **CAM2602** or alisertib concentrations in tumor and plasma samples taken at 8 or 12 h after dosing with 200 and 30 mg/kg, respectively. (D) NSG mice bearing subcutaneous, solid tumor xenografts of Jurkat cells were dosed orally once per day with either vehicle, **CAM2602,** or alisertib, as indicated (*n* = 5). Tumor volumes were estimated periodically over the 26 days of dosing by calliper measurement. Error bars show the standard deviation from the mean.

It has previously been shown that PH3 accumulation in tumor cells treated with Aurora A inhibitors is detectable from as early as 4–6 h with microscopy.^32,59^ This suggests a sensitivity advantage for techniques that can resolve mitotic cells in asynchronous cell samples, so we next explored flow cytometry for the detection of PH3 and P-T288 changes in Jurkat cells treated *in vitro* with varying GI_50_-multiples of compound **7** or a vehicle control for 8 h. Supporting the validation of PH3 immunostaining in these samples, this marker was only detectable in mitotic cells, identifiable by their 4n DNA. Samples treated with compound **7** demonstrated a consistent increase in PH3-positive mitotic cells compared to those treated with vehicle controls (Figure S7A,B). A 2× GI_50_ dose of compound **7** yielded almost a 3-fold increase in mitotic cells compared to DMSO exposure, with a similar magnitude of increase at a 5× GI_50_ dose. Complementing the PH3 data, decreased P-Thr288 Aurora A was observed in the mitotic cells treated with compound **7**. This detection of biomarker modulation was repeated for the lead compound **CAM2602**, with alisertib as a positive control using Jurkat cells *in vitro* (Figure 5A). Under these conditions, both **CAM2602** and alisertib treatments exhibited similar evidence of inhibition of Aurora A phosphorylation.

#### PPI Inhibitor of Aurora A-TPX2 Demonstrates *In Vivo* Activity

Given the favorable ADMET profile of **CAM2602** (Table S3) and its ability to modulate biomarkers of target engagement *in vitro*, we next sought to demonstrate that **CAM2602** could affect tumor cell biomarker modulation *in vivo* following acute systemic administration in a mouse xenograft model.

We first assessed the pharmacokinetics of **CAM2602** by administering the compound at 3 separate doses in female CD-1 mice and measuring the total concentration of compound in plasma over time (Figure S8). The intravenous dose is cleared in a first-order elimination process. At higher doses, administered orally, the concentration of compounds rapidly reaches a plateau that is maintained for at least 8 h. These clearance profiles suggest that one or more clearance mechanisms, i.e. efflux and/or metabolism, may be saturated at these compound doses. The oral bioavailability of **CAM2602** at 50 mg/kg was 99.8% while no weight loss or adverse events were observed in any PK studies (Figure S9).

For the xenograft model, Jurkat cells were engrafted as a subcutaneous, solid tumor in the flanks of NOD SCID gamma (NSG) mice. Xenografted mice were orally administered a single dose of 200 mg/kg **CAM2602**, 30 mg/kg of alisertib or vehicle, based on our earlier PK data for **CAM2602** (Figure S8) or previously reported studies using alisertib.^32,63,64^ Tumor and plasma samples were then taken 8 or 12 h postdosing. Resected tumors were digested into single cell aspirates, fixed, and processed using flow cytometry to detect modulation of PH3 and P-Thr288 biomarkers (Figure 5B). At both 8 and 12 h postdosing, xenografted tumor cells from **CAM2602**-treated mice demonstrated fold-increases in PH3 over vehicle controls, matching those seen previously *in vitro* (Figure 5A,B). Across the **CAM2602**-treated tumor samples, decreases in the Aurora A P-Thr288 marker were also evident, but changes to this marker were considerably less pronounced than those seen for *in vitro* conditions and were not significant. Plasma and tumor concentrations of **CAM2602** exhibited high micromolar concentrations of the compound in both compartments at both 8 and 12 h time points (Figure 5C). When adjusted for mouse plasma protein binding (Table S3), the predicted free drug concentrations in plasma (5.4 μM at 8 h and 2.2 μM at 12 h) are well in excess of the *K*_D_ (20 nM) for the target, supportive of likely target engagement. Moreover, the measured tumor concentrations (70 μM at 8 h and 54 μM at 12 h) suggest meaningful tissue exposure consistent with levels required for inhibition in cells up to 12 h postdosing. Contrary to our *in vitro* data (Figure 5A), tumor samples recovered from alisertib-treated mice yielded a decrease in PH3 at 8 h, and neither 8 or 12 h samples yielded the increase in PH3 expected from Aurora A inhibition (Figure 5B). Tumor and plasma PK measurements at 8 and 12 h postdosing with alisertib indicated either micromolar or very high nanomolar tissue concentrations for this potent inhibitor (Figure 5C). Alisertib is likely to have off-target activity against Aurora B at these high concentrations, which might be expected to decrease PH3, therefore overriding the increase in PH3 expected from Aurora A inhibition.^62,64^

#### CAM2602 Induces Growth Suppression of Tumor Xenografts

Tolerability studies with 50, 100, and 150 mg/kg administered to NSG mice (daily dosing for 7 days, followed by 7 days without dosing) indicated that the highest dose examined of 150 mg/kg was tolerated without overt toxicity (Figure S9). An efficacy study was performed using xenografted NSG mice bearing subcutaneous Jurkat cells implanted as solid tumors with a daily oral dose of either 100 or 150 mg/kg of **CAM2602**, 20 mg/kg of alisertib, or vehicle for 26 days. Tumor volume measurements were taken three times per week during this time. The volume data indicated that vehicle-treated mice exhibited continuous tumor growth during the study, whereas the two doses of **CAM2602** were capable of successfully reducing tumor growth, the higher of the two doses having the greater effect (Figure 5D). Alisertib had the greatest impact on tumor growth, likely due to the higher potency of this inhibitor. In agreement with earlier assessments of toxicity, there were no observations of toxic phenomena among the treated mice for the duration of the study, and no evidence of loss of body weight (data not shown). Inhibition of Aurora kinases with ATP-competitive inhibitors has previously been linked to dose-limiting toxicities such as bone marrow ablation and neutropenia.^17,47^ Possible loss of blood cell lineages indicative of such toxicities was additionally analyzed using blood samples taken from all mice upon completion of the efficacy study. These analyses indicated a very mild anemic response in all nonvehicle dosing group animals with a slight drop in hematocrit readings, but this was coincident in all cases with an elevation in the reticulocyte count (Figure S10).

Aurora A overexpression is known to drive resistance to taxanes in cancer cells.^12,13,65^ In addition, compelling data indicate that inhibition of Aurora A synergises with paclitaxel in cell lines exhibiting Aurora A amplification.^66^ Using an earlier compound in our series, compound **6**, with an analogous structure and mode of action to **CAM2602,** we were able to demonstrate drug synergy with paclitaxel in the pancreatic cell line PANC-1, emulating benefits previously observed for ATP-competitive Aurora A inhibitors (Figure S11). Considering the dose-limiting toxicities associated with paclitaxel in the clinic, a major therapeutic implication of these results could be the potential to greatly reduce the required doses of paclitaxel when applied in combination with a drug targeting the Aurora A-TPX2 PPI. A prediction for Aurora A inhibition, including PPI-targeting agents, is the reversal of taxane resistance, which suggests a promising clinical opportunity to treat tumors with combinations of these agents.^12,13,65,66^ Taxane resistance is a major clinical challenge with nearly half of all patients exhibiting primary resistance or eventually relapsing with treatment-resistant disease; agents that reverse taxane resistance would find utility in epithelial ovarian cancers, mammary adenocarcinomas, and nonsmall cell lung carcinomas, for example.^67−70^

## Discussion and Conclusions

Small molecule inhibition of Aurora A is an attractive strategy for the treatment of a wide range of human malignancies.^3−5,12,14−16^ Consequently, several high-potency, orthosteric, ATP-competitive inhibitors of Aurora A have been developed.^17^ Encouraging trial data have been seen for one such inhibitor, alisertib, across a range of cancers, but significant dose-limiting toxicities are consistently observed.^31^ The promise of PPI inhibitors of kinases is that they bind to less conserved sites in the target and are more likely to exhibit better selectivity than orthosteric ATP-competitive molecules.^38,71^ Therefore, small molecule inhibitors targeting PPIs potentially exhibit fewer off-target toxicities and can have reduced propensity to develop resistance in cancer cells.^38−40^ TPX2 is a particularly promising binding partner to block in this way, exhibiting a broad repertoire of activity-promoting properties in relation to Aurora A.^1,20,24^

We have developed through fragment-based, structure-guided approaches a series of novel compounds that inhibit a PPI between Aurora A and TPX2. The initial fragment hits identified from screening with the ATP site blocked by a high-affinity inhibitor were very weakly active, but guided by continuous crystallographic analysis of the inhibitors in complex with Aurora A, we were able to increase target affinity by more than 10,000-fold, clearly demonstrating the ability of fragment-based and structural biology approaches to develop potent PPI inhibitors when a suitable binding pocket is present. These compounds occupy a hydrophobic pocket on the surface of Aurora A, discrete from its ATP-binding catalytic site, which forms the interaction surface for a linear N-terminal segment of the interacting peptide from TPX2. They displace critical interactions made by the Tyr8 and Tyr10 residues of TPX2 with Aurora A, directly inhibiting the binding of TPX2 to a key hot spot in Aurora A.^34,72^ Notably, the compounds interact with Aurora residues that are not conserved in the closely related Aurora B kinase, providing a structural rationale for their high selectivity.

These are the first high-affinity ligands inhibiting this allosteric site, and our lead compound **CAM2602** has pharmacological properties that enable it to be used in *in vivo* studies. We find that these compounds are cytotoxic to cancer cells alone or in a synergistic combination with paclitaxel, with their cytotoxic effects proportional to target engagement marked by Aurora A mislocalization and dephosphorylation on Thr288.

In a solid tumor xenograft model, oral delivery of **CAM2602** successfully elicited biomarkers of target engagement, increasing PH3-positive cells and decreasing the proportion of those cells positive for P-Thr288 Aurora A; moreover, this compound also reduced tumor growth. These results show that an inhibitor of the Aurora A-TPX2 PPI is a viable route to a therapeutic intervention in cancer.

The lack of overt toxicity seen *in vitro* and particularly in *in vivo* studies with lead compound **CAM2602** is noteworthy. Considering the high doses we administered to deliver sufficient drug levels intra-tumourally, we expected to observe toxicity similar to that seen with ATP-competitive Aurora A inhibitors in the clinic.^31^ However, such toxicity did not limit the practical utility of **CAM2602** in our sustained multidose efficacy study. This apparent lack of toxicity may reflect the particularly high target specificity which is characteristic of enzyme inhibition by the PPI mode rather than at the ATP-binding pocket.^38,39^ We cannot rule out the possibility that some of the effects of **CAM2602** are driven by off-target activity. However, the free drug concentration in the solid tumor xenograft study at 8 h was 5.4 μM, which given the prolonged plateau in the pharmacokinetics (Figure S8) suggests that the maximum exposures in this study were likely around these levels. The selectivity data for **CAM2602** at 10 μM (Table S2) and the kinase selectivity for a representative compound from the series at the same concentration (Figure S3) were excellent suggesting that at these concentrations, there is likely to be little engagement with off-targets while the biomarker data strongly support target engagement. We therefore conclude that it is likely that the efficacy seen in this study is due to inhibition of the Aurora A:TPX2 interaction. In conclusion, we have developed a small molecule inhibitor of the Aurora A:TPX2 interaction, for which we provide a first example of efficacy in a xenograft model, providing a proof of concept for further development. In addition, the encouraging *in vitro* synergy demonstrated with paclitaxel suggests an important clinical modality for this new class of inhibitors.

During the course of this work, Bayliss and co-workers have published the results of two crystallographic fragment screens against Aurora A.^34,35^ Our target pocket, where tyrosines 8 and 10 of TPX2 bind, was identified as one of the hot spots for this PPI, and a number of diverse fragments were found in this pocket, providing possibilities for further development of Aurora A:TPX2 inhibitors.

Abbreviations: SAR, structure activity relationship.

## Experimental Section

### Cell Culture

HeLa, PANC-1, and Jurkat cells were maintained in humidified incubators at 37 °C, 5% CO_2_ using either DMEM (HeLa and PANC-1: high glucose, GlutaMAX Supplement, pyruvate; ThermoFisher Scientific 10569010) or RPMI 1640 (Jurkat and PBMC: GlutaMAX Supplement, HEPES; ThermoFisher Scientific 72400021) medium supplemented with 10% fetal bovine serum. As a positive control in the high-content screening assays, we made use of a previously reported stable HeLa FlpIn TREx cell line expressing a fusion mCherry-TPX2-1-43 protein which was inducible upon addition of doxycycline (0.5 mg mL^–1^).^37^ New vials of PBMCs were obtained for each viability experiment (ATCC, PCS-800-011).

### Viability Assays

Cells were seeded onto sterile, flat-bottomed, 96-well tissue culture plates in antibiotic-free media; HeLa cells were seeded the day before treatment at a density of 5 × 10^3^ per well, whereas Jurkat and PBMCs were seeded at 2 × 10^4^ or 1 × 10^5^ per well, respectively, on the day of treatment. All wells per plate contained 100 μL of cells and/or media, and the outermost wells of each plate contained media-only controls. On the day of treatment, 10-point, 2-fold dilution series of each compound were prepared in antibiotic-free media on separate, sterile, round-bottomed 96-well plates. All series concentrations were adjusted to 5-fold higher than the intended final concentrations before 25 μL of these were then pipetted in triplicate to the flat-bottomed plates with cells, yielding a final volume of 125 μL per well. Matching DMSO-treatment dilution series were included in triplicate on each plate. Media-only edge wells received 25 μL of media to maintain equal final volumes across all wells on the plates, which were then sealed with sterile, breathable membranes beneath the plate lids and incubated in humidified incubators at 37 °C, 5% CO_2_ for 72 h. Depending on cell line, cell growth per well was assessed using the CellTiter-Blue assay (Promega; Jurkat cells) or sulforhodamine B assay (HeLa cells). Cell-free control wells were used to calculate assay blanks for subtraction from assay values per treatment condition per plate; triplicate means of corresponding DMSO control well assay values were used to determine fold-survival values for each compound treatment condition. GI_50_ values were calculated from four-parameter dose–response curves that were fitted using the GraphPad Prism software (La Jolla, CA).

### High-Content Screening

The high-content imaging Aurora A mislocalization and Thr288 dephosphorylation assays have been described previously by our lab.^37^ Briefly, 24 h after seeding 8 × 10^3^ HeLa cells in 100 μL of media per well of tissue culture-treated 96-well plates (ThermoFisher, 167008), the cells were treated with 9-point, 2-fold titrations of compound in media for 2 h under standard tissue culture conditions. Drugging volumes were managed as described above for the viability assays (i.e., 25 μL is added to a final volume of 125 μL on cells to yield 5× dilution). Drugging media was supplemented to give a final concentration of 10 μM bortezomib (Selleck Chemicals) to reduce numbers of anaphase cells yielding false-positivity during image analysis. Following 2 h incubation under drugging conditions, the plates were aspirated, fixed, permeabilized, and stained as described before.

Imaging of the plates was performed on an ImageXpress Micro Confocal High-Content Imaging System (Molecular Devices) using a 20× ELWD objective (optimal for 96-well plates with standard 1.9 mm thick transparent bases) and laser autofocus per field. For each well, 12 nonoverlapping fields in 3 fluorescent channels were acquired with bright-field optics and 2 × 2 binning, which allowed for approximately 100 mitotic cell observations per triplicate well. Custom Module Editor (CME) image analysis software (Molecular Devices) was used to quantify mitotic cell phenotypic responses, which were used to calculate assay end points.

Aurora A mislocalization assay image data were analyzed in CME by using Hoechst/DAPI channel image data to locate all individual nuclei per field. Corresponding TPX2/Cy5 channel image data were used to identify the mitotic cell subpopulations in each field through TPX2-positivity of their nuclei. Intensity thresholds >100 times that of the image background were set in CME to distinguish DAPI and FITC channel signal from any noise. For each mitotic nucleus a top-hat filter with a 25 μm kernel was used to define a fine mitotic spindle mask. Per mitotic spindle mask, the corresponding average Aurora A/FITC channel intensity was measured. The resulting cell-level data were exported and analyzed in Excel whereby the highest spindle Aurora A intensity in the darkest 10% of mitotic cells from untreated control wells was used to set a per-plate assay threshold below which Aurora A was classified as delocalized from the spindle. The assay threshold was then applied across all mitotic cells recorded per well, and the percentage of cells with Aurora A intensity in the spindle mask below the threshold was reported as the percentage of mitotic cells per well with mislocalized Aurora A. The Thr288 dephosphorylation assay was performed and analyzed the same way as for the mislocalization assay, but substituted PH3 and P-Thr288 Aurora A antibodies for TPX2 and total Aurora A, respectively. In this case, PH3-positivity was used to identify mitotic cells and the mitotic spindle mask was replaced with a whole-nucleus mask for the purpose of measuring P-Thr288 loss. A percentage of mitotic cells per well exhibiting dephosphorylated Thr288 Aurora A measure used the same assay threshold calculation as used for the mislocalization assay. The diagram of the imaging scheme and image analysis are shown in Supplemental Figure S13.

### Confocal Microscopy

HeLa cells were grown on sterile type-I borosilicate glass coverslips placed in 6-well tissue culture plates with 2 × 10^5^ cells per well. Twenty-four h following seeding, the cells were treated as indicated; then, the media was aspirated, and the cells were fixed using ice-cold methanol for 10 min. Fixed cells were permeabilized with 0.1% Triton-X100, 0.1% TWEEN20 in PBS for 10 min at room temperature before being washed in blocking buffer (3% BSA, 0.1% TWEEN20 in PBS) for 30 min. Anti-Aurora A (Abcam, ab52973, 1:500) and anti-tubulin (Abcam, ab6160, 1:500) were diluted in blocking buffer and used to probe the cells for 30 min at room temperature. Excess antibody was washed with 3 rounds of 0.1% TWEEN20 in PBS, followed by probing with secondary antibodies (goat anti-rabbit Alexafluor 488, A11034, 1:500; goat anti-rat Alexa Fluor 647, A21247, 1:500, ThermoFisher Scientific) applied and washed as per the primary antibodies, supplemented with 4 μg/mL Hoeschst 33342. Imaging was performed on a Leica SP5 confocal microscope using a 100 × 1.4 NA oil objective. Maximum projection images were created with z-stacks taken at 1 μm intervals. Pixel intensities were kept subsaturation. Laser exposure and detector settings were identical across an experiment to allow comparison between samples.

### Flow Cytometry

Jurkat cells from either tissue culture or resected tumor xenografts were washed, fixed, and permeabilized using reagents from BD Biosciences (Stain Buffer, 554657; BD Cytofix, 554655; Perm Buffer III, 558050). Ideally, 1.5 × 10^6^ cells per sample were washed once with 500 μL of cold stain buffer and transferred to clean 1.5 mL centrifuge tubes. Samples were pelleted and aspirated before fixing with 250 μL of BD Cytofix buffer, following a brief vortex in the fixative and incubation on ice for 15 min. The fixed cells were then washed as before and subsequently pelleted and aspirated prior to being permeabilized by slow addition of 500 μL of cold Perm Buffer III while vortexing. Samples were incubated on ice for 30 min and then washed as before. The cells were then sequentially stained in three steps with anti-Aurora A P-Thr288 (1:100, Cell Signaling no. 3079), goat anti-rabbit Alexa Fluor 555 (1:500, Life Technologies no. A21429), and finally Alexa Fluor 647-conjugated anti-histone H3 (phospho-S10, 1:400, Cell Signaling #3458). For resected xenograft samples, Alexa Fluor 488-conjugated human-specific anti-CD3 (1:200, BD Pharmingen 557694) was included in the final staining step to allow the exclusion of possible host cell contamination. The sequence of antibody staining is required to avoid species cross-reactivity among the chosen antibodies. The antibodies were applied to the cell samples in 100 μL of staining buffer, incubated for 30 min at room temperature with rotation, and washed twice in 500 μL of stain buffer between each antibody step. Cells remained in the final wash supplemented with 4 μg/mL of Hoechst 33342 and 250 μg/mL of RNase A. The cells were transferred to flow cytometry tubes and incubated in the dark at room temperature for 30 min before being analyzed. Analysis of flow cytometry samples was performed on a BD LSRFortessa equipped to excite the samples at 355, 488, and 640 nm and to resolve the fluorescent probes using separate detectors. Experiment data were analyzed using FlowJo Ver.10 software (FlowJo, LLC). Gating strategies are shown in Figure S7.

### Western Blotting

Total protein was isolated by directly lysing the cells in nondenaturing lysis buffer (50 mM HEPES-HCl pH7.4, 250 mM NaCl, 0.2% Triton X-100, 1 mM EDTA, 1 mM dithiothreitol, 1 mM NaF, 10 mM β-glycerophosphate, 0.1 mM Na_3_VO_4_, 1× Roche cOmplete protease inhibitors). Protein lysates (12 μg per lane) were resolved on SDS-PAGE gels, transferred onto an Immobilon-P, PVDF membrane (0.45 μm, Millipore), and probed with either anti-histone H3 (1:1000, NEB, 9715S) or anti-histone H3 (phosphor S10, 1:2000, Abcam, ab14955). Secondary HRP-conjugated antibodies were used (GE Healthcare), and the signal was detected using an Amersham enhanced chemiluminescence system (ECL, GE Healthcare).

### *In Vivo* Studies

*In vivo* pharmacodynamics, tolerability, and efficacy studies were carried out by Axis Bioservices Ltd. (Northern Ireland). Pharmacokinetic work was carried out at WuXi AppTech (China). Female CD-1 mice were used in pharmacokinetics studies, and female NOD-SCID gamma (NSG) mice were used for all other *in vivo* studies. For xenograft studies, Jurkat E6.1 cells (ATCC) were bulk-grown in RPMI 1640 media (GlutaMAX Supplement, HEPES; ThermoFisher Scientific 72400021) supplemented with 10% fetal bovine serum. Tumor cell implantation employed 2 × 10^7^ cells in matrigel per tumor, injected subcutaneously to the rear dorsum. Tumor volumes postimplantation were monitored using caliper measurements and mice were advanced for treatment when tumor volumes between 150 mm and 200 mm^3^ were reached. Where used, compounds were formulated in DMSO:20% HP-β-CD (2-hydroxypropyl-beta-cyclodextrin in PBS, 2.5:97.5) with pH adjusted to 7.6. All treatments were administered by oral gavage.

For pharmacodynamic biomarker studies, mice aged 5–7 weeks at the time of implantation were administered single doses of the indicated treatments and were harvested for tumor resection and collection of whole blood by cardiac puncture at 0, 8, or 12 h postdosing. Plasma samples were submitted for PK analysis (Xenogesis Ltd.). Resected tumors were digested to single cell aspirates in dissociation buffer (RPMI medium supplemented with 5% FBS, collagenase type I (200 U/mL), and DNase I (100 μg/mL)) for 30 min at 37 °C with periodic vortexing and passed through a 70 μm filter with PBS washes. Tumor samples were cryogenically frozen and stored prior to being processed for flow cytometry as described above. Efficacy studies employed xenografted mice aged 6–8 weeks. Dosing was applied daily for 26 days, and tumor volumes (4/3π*r*^3^) were recorded three times per week by caliper measurements using three reference diameters to estimate geometric mean diameter. Samples were harvested 8 h after the final dose. Tolerability studies used nonxenografted mice aged 6–8 weeks. Doses were applied daily for 7 days, followed by a 7 day period with no treatment. Animal bodyweight, behavior, and appearance were monitored daily. All protocols to be used in this study have been approved by the Axis Bioservices Animal Welfare and Ethical Review Board, and all procedures are carried out under the guidelines of the Animal (Scientific Procedures) Act 1986.

### Synergy Analysis

Drug synergy experiments using the Bliss independence model were performed as previously reported.^64^ 96-well plates were seeded with 5 × 10^3^ PANC-1 cells per well 24 h prior to drugging with a dilution series of each drug in an 8 × 8 checkerboard pattern of combinations. For both drugs, the lowest drug concentration value in each series was a no-drug vehicle control, which allowed for true single-agent dosing to be represented among the permutations of drug ratios tested. After SRB staining to obtain the growth inhibition data, we used SynergyFinder Web server (https://synergyfinder.org/)^71^ to identify synergistic drug combinations. The single-agent inhibition values were used to calculate the drug combination surface under the assumption of an additive effect. Regions of synergy were then detected by comparing the observed combination data with the corresponding predicted values assuming additivity. In the final synergy plots, positive values indicate synergy regions, whereas negative difference values identify antagonistic effects.

### Protein Production

Aurora A was expressed from pBAT4 or pHAT4 plasmid^72^ in double cistronic construct with λ phosphatase, without which Aurora A was toxic to *E. coli*. Aurora A for biophysical assays was expressed from plasmid pBAT4-AurAS.003 which encoded for the kinase domain only (residues 126–390) of human Aurora A (Uniprot: O14965) followed by a hexa-His tag. Deletion of the N-terminal localization domain implied the additional benefit of removing a region of the protein that was predicted to be intrinsically disordered. Further tailoring of the construct N- and C-termini was based on expression levels. For crystallography, Aurora A contained also mutations Thr287Ala or Cys290Ala to reduce heterogeneity by activation loop phosphorylation and intermolecular disulfide bond formation, respectively. For earlier compounds, a longer (residues 126–391) version of the protein without a C-terminal His-tag was used for crystallization, as described in Janeček et al.^37^ Aurora B protein was expressed from plasmid pNIC28-AurB (Addgene no. 39119).

Aurora A and B proteins were prepared using the same protocol. The protein expression was carried in the BL21(DE3) strain (which was supplemented with pUBS520 plasmid for rare-Arg codon compensation for Aurora A) in 2YT media with 100 μg/mL of ampicillin. The cells were grown in shaker flasks to OD of 0.8–1.0 and expression induced with 400 μM isopropyl-thio-β-glycopyranoside for 3 h at 37 °C. Cells were harvested by centrifugation, and pellets were stored at −20 °C. Cells were resuspended in 50 mM HEPES pH 7.4, 1 M NaCl, 100 mM Mg acetate, 1 mM ATP/1 mM ADP, 25 mM imidazole, and 5 mM β-mercaptoethanol, with one tablet of protease inhibitors (cOmplete Protease Inhibitor Cocktails, Roche) and 500 μL of 2 mg/mL DNase I (Sigma: DN25). Cells were lysed with sonication or using an Emulsiflex C3 homogenizer and lysate clarified by centrifugation at 30,000*g* for 30 min at 4 °C. The supernatant was filtered and protein purified with automated two-step protocol using an ÄKTA Pure chromatography system. The protein was captured in 5 mL FF HisTrap column (Cytiva) and washed with 50 mM HEPES/Na pH 7.4, 500 mM NaCl, 100 mM magnesium acetate, 1 mM ATP/1 mM ADP, 40 mM imidazole, 5 mM β-mercaptoethanol, and 10% v/v glycerol until baseline stabilized. Protein was eluted in reverse flow with 50 mM HEPES/Na pH 7.4, 500 mM NaCl, 100 mM Mg acetate, 1 mM ATP/1 mM ADP, 600 mM imidazole, 5 mM β-mercaptoethanol, 10% v/v glycerol, and the eluted protein directed to injection loop and injected directly to HiLoad 16/60 Superdex 75 pg column (Cytiva) which had been equilibrated with 50 mM HEPES pH 7.4, 50 mM NaCl, 100 mM Mg acetate, 1 mM ADP, 0.5 mM TCEP, 10% v/v glycerol, and column ran at 1 mL/min. Peak fractions were pooled, concentrated, and stored in flash-frozen aliquots at −80 °C.

TPX2 peptide (residues 7–43, Uniprot: Q9ULW0) with a C-terminal GGGCSS tail was expressed in *E. coli* as a GB1 fusion with an N-terminal His-tag and HRV 3C protease cleavage site for tag removal in vector pOP3BP, as described above. A pellet from 2 L culture was resuspended in 50 mM HEPES pH 7.4, 500 mM NaCl, 40 mM imidazole, 10% glycerol, 0.5 mM TCEP and 500 μL of DNase I (2 mg/mL) and lysed using a sonicator. Lysate was centrifuged for 30 min at 150,000*g* and filtered supernatant loaded on 1 mL gravity flow Ni Sepharose column (Cube Biotech). After washing with lysis buffer, the protein was eluted with 50 mM HEPES pH 7.4, 500 mM NaCl, 300 mM imidazole, 10% glycerol, 0.5 mM TCEP. Peak fractions were pooled and buffer exchanged with PD-10 column to remove imidazole and glycerol. Alexa Fluor 488 C5 maleimide (A10254, Thermo Fisher Scientific) was added to the protein sample in 25-fold molar excess to label the C-terminal cysteine for 2 h at room temperature. The reaction was terminated with excess cysteine and protein cleaved with HRV 3C protease overnight. The cleaved protein was passed through second Ni Sepharose column to remove fusion protein and uncleaved material. Labeled peptide was purified by a reversed phase chromatography using HiChrom 300 Å 4.6 × 250 mm C18 column with gradient elution from 10% acetonitrile, 0.1% trifluoroacetic acid to 90% acetonitrile at 3 mL/min flow rate, dried under vacuum, resuspended in 50 mM HEPES pH 7.4, 100 mM Mg acetate, 50 mM NaCl and stored at −80 °C in dark.

Sequence of the peptide used in the assay is shown below, with TPX2 part underlined and cysteine that was labeled with Alexa Fluor 488 is highlighted in bold in italics. GPGSYSYDAPSDFINFSSLDDEGDTQNIDSWFEEKANLENLKGGG***C***SS.

### Fluorescence Polarization (FP) Assay

The FP assay was done using a BMG Pherastar FS plate reader with a gain of 20% and target 90 mP. The *K*_D_ for TPX2 binding to Aurora A was determined to be 1.2 nM by direct titration of up to 200 nM of Aurora A protein to 11 nM labeled TPX2 peptide in 100 mM HEPES pH 7.4, 100 mM magnesium acetate, 50 mM NaCl, 0.02% P20, 1 mM DTT, 1 mM ATP, 10% (v/v) DMSO. The competition FP assay was run in the same buffer with 10 nM TPX2 peptide and 30 nM Aurora A. Twelve concentrations of compounds were used as competitors in triplicate. The data were monitored for both anisotropy and for change in total fluorescence to account for any artifacts, such as compound interference or aggregation. The resulting competitive binding isotherms were measured and fitted using the expression described by Wang^70^ using the Pro Fit software package (Quan Soft).

### Isothermal Titration Calorimetry

Isothermal titration calorimetry (ITC) was performed using a Microcal itc200 instrument at 25 °C, in the following experimental buffer (unless specifically indicated otherwise): 0.1 M HEPES/Na pH 7.4, 0.1 M magnesium acetate, 0.05 M NaCl, with the addition of 10% v/v DMSO, fresh 1 mM ATP and fresh 0.25 mM TCEP.

Prior to the experiment, Aurora A protein was thawed and buffer exchanged in the experimental buffer using NAP-5 Columns (GE Healthcare). Experiments typically involved titrating 25 μM protein in the sample cell with 300 μM compound in the syringe. The raw ITC data were fitted using a single site binding model using the Microcal ITC LLC data analysis program in the Origin 7.0 package.

### Crystallization and Structure Determination

To a solution of 3.8 mg/mL of Aurora A SilverBullet screen solution 82 (Hampton Research), *trans*-1,2-cyclohexanedicarboxylic acid was added to a final concentration of 8% by volume, and the sample was centrifuged for 5 min at room temperature at maximum speed in a microcentrifuge. Crystallization was performed in 96-well “MRC” plates (Molecular Dimensions) using a Mosquito nanoliter robot (TTP Labtech) with 300 nL + 300 nL drop with 30% PEG5000 MME (28–32%), 0.1 M (NH_4_)_2_SO_4_, 0.1 M MES pH 6.5 as the mother liquor. For soaking, 1 μL of 100 mM compound in DMSO was diluted with 9 μL of 30% PEG5000 MME (28–32%), 0.1 M (NH_4_)_2_SO_4_, and 0.1 M MES pH 6.5 and added to the crystals between 2 h and overnight. Crystals were collected into a nylon loop and flash cooled in liquid nitrogen and stored for data collection. Data collection was typically done for 180 images at 1° oscillation per image at Diamond Light Source beamlines I04-1, I03 and I24. Data reduction and automatic structure determination were done using the pipedream workflow from Global Phasing Ltd. with automatic ligand fitting. Ligand restraints were generated with grade and mogul from CCDC. The structure was analyzed and corrected using Coot and refined with autoBuster. Final ligand electron densities are shown in Figure S12. Data collection and structure refinement statistics are listed in Table S4.

### General Chemistry Methods

Unless otherwise stated, starting materials and reagents were purchased from regular suppliers. Dry solvents were purchased and used as provided. Thin layer chromatography (TLC) was performed on glass plates coated with Merck 60 F254 silica, and visualization was achieved by UV light or by staining with potassium permanganate. Flash column chromatography was using a Biotage Isolera One and Biotage Isolera Four systems with UV detection at 254 and 280 nm and commercially available cartridges. ^1^H NMR spectra were recorded on a Bruker Avance 400 (400 MHz) or Bruker Avance Cryo 500 (500 MHz). Chemical shifts are quoted in ppm and are referenced to the residual nondeuterated solvent peak, and are reported (based on appearance rather than interpretation) as follows: chemical shift δ/ppm (multiplicity, coupling constant *J*/Hz, number of protons) [br, broad; s, singlet; d, doublet; t, triplet; q, quartet; qui, quintet; sept, septet; m, multiplet]. All *J* values are given in Hz. High-resolution mass measurements were performed on a Waters LCT Premier mass spectrometer or a Kratos Concept mass spectrometer. Low-resolution measurements were recorded on a Waters/ZQ LCMS and on a Waters Acquity UPLC HClass LCMS. The method parameters are provided in Table 1.

**Table 1:** Method Parameters

column	additive	flow rate	gradient (time, %MeCN in H_2_O)
HSS C18 (100 Å, 1.8 μm, 2.1 mm × 50 mm)	0.1% HCO_2_H	0.6 mL/min	0 min, 5%; 0.8 min, 5%; 8.3 min, 95%; 9.3 min, 95%; 9.5 min, 5%; 10.5 min, 5%

Abbreviations: TEA: triethylamine; DCM: dichloromethane; DME: dimethoxyethane; CDI: carbonyldiimidazole; DBU: 1,8-Diazabicyclo[5.4.0]undec-7-ene; LCMS: liquid chromatography–mass spectrometry; FC: flash chromatography

All compounds are >95% pure by HPLC analyses. Synthetic routes are reported in the SI (Schemes S1–S5), along with NMR and LCMS spectra for final compounds (Figures S14–S31).

### Method A—Suzuki Coupling

Aryl bromide (1 equiv), boronic acid (1 equiv), and triethylamine (3 equiv) were dissolved in DME (1.5 mL) and water (0.5 mL), and nitrogen was bubbled through for 10 min. Pd(dppf)Cl_2_·CH_2_Cl_2_ (10 mol %) was added, and the reaction was heated with microwave irradiation at 120 °C for 30 min. After cooling to room temperature, the solvents were evaporated in vacuo. The crude residue was dissolved in DCM (10 mL), filtered through a hydrophobic frit, and then evaporated and purified by FC (SiO_2_, 10–100% EA in pet ether 40–60) to give the product.

### Method B—Ester Hydrolysis, Thermal

The methyl ester (1 equiv) was dissolved in THF (2 mL) and H_2_O (2 mL), and LiOH (or NaOH if specified) (3 equiv) was added and stirred overnight at 45 °C. After cooling to room temperature, ethyl acetate (2 × 50 mL) and H_2_O (50 mL) was added, and the organic layers were discarded. The aqueous layer was acidified with dilute HCl to pH 4 and extracted with ethyl acetate (2 × 50 mL) and dried with Na_2_SO_4_, and the solvent was removed in vacuo to give the product.

### Method C—Ester Hydrolysis, Microwave

The methyl ester (1 equiv) and lithium iodide (10 equiv) were dissolved in pyridine (2 mL), and the reaction mixture was heated at 180 °C for 1 h under microwave irradiation. After cooling to room temperature, the solvent was removed in vacuo and the residue was taken up in ethyl acetate (20 mL) and sat. aq. NaHCO_3_ (20 mL). The aqueous layer was carefully acidified (pH 2) and extracted with ethyl acetate (2 × 30 mL). The organic layers were combined, and the solvent was removed in vacuo to give the product.

### Method D1—Biaryl Ether Formation

To a stirred solution of phenol (1.5 mmol) in DMF (1.3 mL) were added potassium carbonate (2.5 mmol) and the appropriate 2-bromopyridine (1.5 mmol). The reaction mixture was stirred at 150 °C overnight. The mixture was diluted in water, and the organic layer was extracted with EtOAc (×3). The combined organic layers were washed with brine and dried over Na_2_SO_4_ and filtered. The filtrate was evaporated in vacuo to obtain the crude which was purified by FC (SiO_2_, 0–25% EA in pet ether 40–60) to provide the product.

### Method D2—Biaryl Ether Formation

A stirred solution of phenol (1–1.5 mmol) and cesium carbonate (2.5 mmol) in dry DMSO (5 mL) was heated at 45 °C for 10 min. The appropriate fluoro-pyrimidine (1 mmol) or fluoro-pyrazine was then added to the mixture, and the mixture was flushed with nitrogen and heated between 65 and 150 °C in sealed microwave vials for 1–16 h according to the starting material’s reactivity. The resulting mixture was poured into water and extracted with EtOAc (3×). The combined organics were dried over Na_2_SO_4_ and filtered. The filtrate was evaporated in vacuo to give a crude product which was purified by FC (SiO_2_, 0–25% EtOAc in pet ether 40–60) to provide the product.

### Method E—Sulfonamide Coupling

A solution of the requisite carboxylic acid (0.21 mmol) and carbonyldiimidazole (0.63 mmol, 3.0 equiv) in THF (6 mL) was heated at 45 °C for 3 h. Then, a solution of DBU (0.84 mmol, 4.0 equiv) and the requisite sulfonamide (0.31 mmol, 1.5 equiv) in THF (2 mL) was added and stirring continued at 80 °C overnight. If the reaction was incomplete after 18 h, additional sulfonamide was added and the mixture was stirred overnight. Upon completion, the reaction mixture was concentrated in vacuo, diluted with DCM:IPA (4:1, 50 mL), washed with 1 M HCl (2 × 25 mL) and brine (25 mL) and then passed through a hydrophobic frit, and the solvent was removed in vacuo. Purification via FC (SiO_2_, 10–60% EtOAc in pet ether 40–60 (both with 0.5% AcOH)) provided the desired product.

### Methyl 4-Bromo-7-methyl-1*H*-indole-6-carboxylate **12**

To a solution of methyl 5-bromo-2-methyl-3-nitrobenzoate **11** (500 mg, 1.82 mmol) in dry THF (18 mL) at −78 °C was added dropwise over 10 min a solution of vinylmagnesium bromide (1.0 M in THF, 5.84 mL, 5.84 mmol, 3.2 equiv). The reaction mixture was stirred at–78 °C for 1.5 h and then allowed to warm to room temperature and stirred overnight. The reaction mixture was quenched by slow addition of NH_4_Cl (5 mL), concentrated in vacuo, resuspended in EtOAc (50 mL), then washed with NH_4_Cl (50 mL) and brine (2 × 50 mL), passed through a hydrophobic frit, and concentrated in vacuo. Purification by FC (SiO_2_, 5–40% EtOAc in pet ether 40–60) gave **12** (208 mg, 43%) as a cream-colored solid. ^1^H NMR (400 MHz, Chloroform-*d*) δ 8.51 (s, 1H), 7.96 (s, 1H), 7.43 (dd, *J* = 2.9, 2.9 Hz, 1H), 6.66 (dd, *J* = 3.2, 2.2 Hz, 1H), 3.94 (s, 3H), 2.78 (s, 3H). LCMS retention time = 2.20 min (100%), and (*m*/*z*) [M – H]^−^ = 266.

### Methyl 7-Methyl-4-(4,4,5,5-tetramethyl-1,3,2-dioxaborolan-2-yl)-1*H*-indole-6-carboxylate **13**

4-Bromo-7-methyl-indole-6-carboxylic acid methyl ester **12** (500 mg, 1.8 mmol, 1 equiv), bis(pinacolato)diboron (585 mg, 2.3 mmol, 1.3 equiv), potassium acetate (521 mg, 5.3 mmol, 3.0 equiv) and Pd(dppf)Cl_2_·DCM (145 mg, 0.18 mmol, 0.1 equiv) were stirred in anhydrous DMSO (1 mL) and heated at 90 °C for 4 h, after which time, the reaction was completed by LC-MS monitoring. The reaction mixture was allowed to cool to room temperature, and water (10 mL) was added. The resulting precipitate was filtered and washed with water (10 mL). The organic residue was taken up in DCM (10 mL), washed with water (10 mL), brine (10 mL), dried (hydrophobic frit), and the solvent was removed in vacuo. The crude material was purified by FC (20–50% EtOAc/Pet ether) to yield an off-white solid 535 mg (96%). ^1^H NMR (400 MHz, CDCl_3_) δ 8.37 (br s, 1H), 8.26 (s, 1H), 7.42 (t, *J* = 2.8 Hz, 1H), 7.10 (t, *J* = 2.8 Hz, 1H), 3.93 (s, 3H), 2.83 (s, 3H), 1.42 (s, 12H). LCMS retention time = 2.20 min (100%), (*m*/*z*) [M – H]^−^ = 314.2, [M + H]^+^ = 316.3.

### 2-(5-Bromo-2-chlorophenoxy)pyridine **14**

5-Bromo-2-chlorophenol (312 mg, 1.50 mmol) and 2-bromopyridine (359 mg, 2.28 mmol) were reacted and purified according to Method D1, in dry DMF (1.3 mL) with K_2_CO_3_ at 150 °C for 18 h, to give the product **14** as a white solid (247 mg, 58%). ^1^H NMR (400 MHz, acetone-d6) δ: 8.10 (dd, *J* = 5.0, 1.6 Hz, 1H), 7.91 (ddd, *J* = 8.2, 7.2, 2.0 Hz, 1H), 7.55–7.46 (m, 3H), 7.18–7.10 (m, 2H). LCMS *m*/*z*: 285.8 (M + H)^+^.

### 4-(4-Chloro-3-(pyridin-2-yloxy)phenyl)-7-methyl-1*H*-indole-6-carboxylic Acid **16**

Methyl 4-(4,4,5,5-tetramethyl-1,3,2-dioxaborolan-2-yl)-1*H*-indole-6-carboxylate **13** (108 mg, 0.342 mmol) and 2-(5-bromo-2-chlorophenoxy)pyridine **14** (107 mg, 0.377 mmol) in DME:water (3:1, 4 mL) were reacted and purified according to Method A to give methyl 4-(4-chloro-3-(pyridin-2-yloxy)phenyl)-7-methyl-1*H*-indole-6-carboxylate **15** (65 mg, 48%). LCMS *m*/*z*: 393.2 (M + H)^+^. The methyl ester **15** (20 mg) was stirred with LiOH (11 mg, 0.254 mmol, 5 equiv) in THF:water (2:1, 1.1 mL) and reacted and purified according to Method B to give the product **16** as a white-off solid (3 mg, 16%). ^1^H NMR (400 MHz, acetone-d6) δ 10.81 (s, 1H), 8.14 (dt, *J* = 5.0, 1.3 Hz, 1H), 7.90 (t, *J* = 1.5 Hz, 1H), 7.88 (s, 1H), 7.69 (d, *J* = 8.8 Hz, 1H), 7.63 (tt, *J* = 5.4, 2.5 Hz, 3H), 7.19–7.10 (m, 2H), 6.76 (dd, *J* = 3.2, 1.8 Hz, 1H), 2.88 (s, 3H). LCMS retention time = 2.13 min (92%), (*m*/*z*) [M – H]^−^ = 377.1, [M + H]^+^ = 379.2.

### 4-(4-Chloro-3-(pyridin-2-yloxy)phenyl)-*N*-(*N*,*N*-dimethylsulfamoyl)-7-methyl-1*H*-indole-6-carboxamide **CAM2602**

Acid **16** (18 mg, 0.05 mmol, 1.0 equiv) and dimethylsulfamide (9 mg, 0.07 mmol, 1.5 equiv) were reacted and purified according to Method E to give the product (5.4 mg, 22%) as a white solid. ^1^H NMR (400 MHz, acetone) δ 10.78 (s, 1H), 8.14 (ddd, *J* = 4.9, 2.1, 0.9 Hz, 1H), 7.90 (ddd, *J* = 8.2, 7.2, 2.0 Hz, 1H), 7.72–7.63 (m, 3H), 7.60 (dt, *J* = 3.0, 1.4 Hz, 1H), 7.50 (s, 1H), 7.18–7.10 (m, 2H), 6.77 (dd, *J* = 3.2, 1.8 Hz, 1H), 3.02 (s, 6H), 2.74 (s, 3H). LCMS retention time = 3.15 min, *m*/*z* (M – H)^−^ = 482.8.

### 4-(4-Chloro-3-(pyridin-2-yloxy)phenyl)-*N*-(cyclopropylsulfonyl)-7-methyl-1*H*-indole-6-carboxamide **10**

Acid **16** (16 mg, 0.042 mmol, 1.0 equiv), carbonyldiimidazole (21 mg, 0.13 mmol), and cyclopropanesulfonamide (7 mg, 0.058 mmol) were reacted in THF (1 mL) according to Method E. The crude was purified by preparative HPLC (Column: Supelco Supelcosil LC-18, 5–95% ACN in water + 0.1% formic acid) to give the product as a white solid (6.0 mg, 29%). ^1^H NMR (400 MHz, acetone-d6) δ 10.82 (s, 1H), 8.14 (dd, *J* = 4.5, 1.6 Hz, 2H), 7.94–7.86 (m, 1H), 7.71–7.64 (m, 3H), 7.63–7.60 (m, 1H), 7.51 (s, 1H), 7.19–7.10 (m, 2H), 6.77 (d, *J* = 3.2 Hz, 1H), 3.22 (tt, *J* = 8.1, 4.8 Hz, 1H), 1.33–1.24 (m, 2H), 1.22–1.12 (m, 2H). LCMS retention time = 3.46 min (100%), *m*/*z* (M – H)^−^ = 480.0, (M + H)^+^ = 482.2.

### Methyl 4-(4-Chlorophenyl)-7-methyl-1*H*-indole-6-carboxylate **17**

Compound **12** (220 mg, 0.82 mmol) and 4-chlorophenylboronic acid (154 mg, 0.98 mmol, 1.2 equiv) were reacted according to Method A. Purification by FC (SiO_2_, 8–66% EtOAc in pet ether 40–60) gave the product as a cream-colored solid (202 mg, 82%). ^1^H NMR (400 MHz, Chloroform-*d*) δ 8.49 (s, 1H), 7.81 (s, 1H), 7.69–7.61 (m, 2H), 7.50–7.42 (m, 3H), 6.74 (dd, *J* = 3.2, 2.1 Hz, 1H), 3.95 (s, 3H), 2.85 (s, 3H). LCMS retention time = 2.46 min (100%), (*m*/*z*) [M – H]^−^ = 298.

### 4-(4-Chlorophenyl)-7-methyl-1*H*-indole-6-carboxylic Acid **4**

Methyl ester **17** (118 mg, 0.39 mmol) was deprotected with LiI (527 mg, 3.94 mmol, 10.0 equiv) according to Method C. Purification by trituration with hexane gave the product as a buff solid (107 mg, 95%).

^1^H NMR (400 MHz, Methanol-*d*_4_) δ 7.73 (s, 1H), 7.71–7.63 (m, 2H), 7.54–7.45 (m, 3H), 6.65 (d, *J* = 3.2 Hz, 1H), 2.84 (s, 3H). LCMS retention time = 2.16 min (100%), (*m*/*z*) [M – H]^−^ = 284.

### 4-(4-Chlorophenyl)-*N*-(cyclopropylsulfonyl)-7-methyl-1*H*-indole-6-carboxamide **7**

Compound **4** (40 mg, 0.14 mmol, 1.0 equiv) and cyclopropane sulfonamide (25 mg, 0.21 mmol, 1.5 equiv) were reacted according to Method E. Purification by FC (SiO_2_, 10–60% EtOAc in pet ether 40–60 (both with 0.5% AcOH)) gave a colorless oil which was dissolved in Et_2_O and then precipitated with hexane to give the product as a white solid (40 mg, 73%). ^1^H NMR (400 MHz, methanol-*d*_4_) δ 7.76–7.67 (m, 2H), 7.55–7.46 (m, 3H), 7.30 (s, 1H), 6.67 (d, *J* = 3.2 Hz, 1H), 3.21 (tt, *J* = 8.0, 4.8 Hz, 1H), 2.73 (s, 3H), 1.36–1.30 (m, 2H), 1.24–1.15 (m, 2H). LCMS retention time = 2.17 min (97%), (*m*/*z*) [M – H]^−^ = 387.

### 4-(4-Chlorophenyl)-*N*-(*N*,*N*-dimethylsulfamoyl)-7-methyl-1*H*-indole-6-carboxamide **8**

Compound **4** (80 mg, 0.27 mmol) and dimethylsulfamide (47 mg, 0.38 mmol) were reacted and purified according to Method E to give the product as a white solid (50 mg, 47%). ^1^H NMR (400 MHz, CD_3_OD) δ 7.71 (d, *J* = 8.0 Hz, 2H), 7.50 (d, *J* = 8.0 Hz, 2H), 7.49 (s, 1H), 7.27 (s, 1H), 6.66 (d, *J* = 2.8 Hz, 1H), 3.04 (s, 6H), 2.70 (s, 3H). LCMS retention time = 2.20 min, *m*/*z* 390.1 (M – H)^−^.

### 2-(5-Bromo-2-chlorophenoxy)pyrazine **19**

5-Bromo-2-chlorophenol (500 mg, 2.41 mmol) and 2-fluoropyrazine (236 mg, 2.41 mmol) were reacted in dry DMSO (4 mL) with CsCO_3_ at 90 °C for 16 h according to Method D2. The crude was purified by FC (SiO_2_, 0–20% EtOAc in Pet ether 40–60) to give the product as a white solid (444 mg, 65%). ^1^H NMR (400 MHz, acetone-d6) δ: 8.60 (d, *J* = 1.4 Hz, 1H), 8.40 (d, *J* = 2.7 Hz, 1H), 8.14 (dd, *J* = 2.7, 1.4 Hz, 1H), 7.66 (dd, *J* = 1.9, 0.6 Hz, 1H), 7.56 (dd, *J* = 2.3, 1.3 Hz, 2H). LCMS retention time = 2.70 min (86%), (*m*/*z*) [M + H]^+^ = 286.6.

### 4-(4-Chloro-3-(pyrazin-2-yloxy)phenyl)-7-methyl-1*H*-indole-6-carboxylic Acid **18**

Methyl 4-(4,4,5,5-tetramethyl-1,3,2-dioxaborolan-2-yl)-1*H*-indole-6-carboxylate **13** (40 mg, 0.13 mmol) and 2-(5-bromo-2-chlorophenoxy)pyrazine **19** (42 mg, 0.15 mmol) were reacted in DME/water = 3:1 (2 mL) according to Method A to give methyl 4-(4-chloro-3-(pyrazin-2-yloxy)phenyl)-7-methyl-1*H*-indole-6-carboxylate as a white solid (44 mg, 84%). LCMS *m*/*z*: 392.2 (M – H)^−^. Methyl 4-(4-chloro-3-(pyrazin-2-yloxy)phenyl)-7-methyl-1*H*-indole-6-carboxylate (44 mg, 0.11 mmol) was reacted with LiOH (23 mg, 0.56 mmol, 5 equiv) in THF/water = 2:1 (2.25 mL) according to Method B to give the product as a white-off solid (7 mg, 16%). ^1^H NMR (400 MHz, DMSO-d6) δ 12.54 (s, 1H), 11.64 (s, 1H), 8.70 (d, *J* = 1.4 Hz, 1H), 8.42 (d, *J* = 2.6 Hz, 1H), 8.23 (dd, *J* = 2.7, 1.4 Hz, 1H), 7.73 (d, *J* = 8.3 Hz, 1H), 7.67–7.59 (m, 4H), 6.63 (dd, *J* = 3.2, 1.8 Hz, 1H), 2.79 (s, 3H). LCMS retention time = 2.01 min (93%), (*m*/*z*) [M – H]^−^ = 378.2, [M + H]^+^ = 380.2.

### 4-(4-Chloro-3-(pyrazin-2-yloxy)phenyl)-*N*-(*N*,*N*-dimethylsulfamoyl)-7-methyl-1*H*-indole-6-carboxamide **9**

4-(4-Chloro-3-(pyrazin-2-yloxy)phenyl)-7-methyl-1*H*-indole-6-carboxylic acid **18** (25 mg, 66 μmol), carbonyldiimidazole (32 mg, 0.198 mmol), and dimethylsulfamide (9 mg, 72 μmol) were reacted in THF (1.5 mL) according to Method E. The crude was purified by preparative HPLC (Column: Supelco Supelcosil LC-18, 5–95% ACN in water + 0.1% formic acid) to give the product as a white solid (6 mg, 19%). ^1^H NMR (400 MHz, acetone-d6) δ 10.81 (s, 1H), 8.62 (d, *J* = 1.4 Hz, 1H), 8.39 (d, *J* = 2.6 Hz, 1H), 8.16 (dd, *J* = 2.7, 1.4 Hz, 1H), 7.78–7.75 (m, 1H), 7.74–7.69 (m, 2H), 7.64–7.59 (m, 1H), 7.51 (s, 1H), 6.80–6.72 (m, 1H), 3.02 (s, 6H), 2.74 (s, 3H). LCMS retention time = 2.10 min (100%), (*m*/*z*) [M – H]^−^ = 484.2, [M + H]^+^ = 486.2.

### Methyl 4-(4-Chloro-3-cyanophenyl)-7-methyl-1*H*-indole-6-carboxylate **20**

Compound **13** (240 mg, 0.90 mmol) and 4-chloro-3-cyanophenylboronic acid (211 mg, 1.16 mmol, 1.3 equiv) were reacted and purified according to Method A. Purification by FC (SiO_2_, 8–66% EtOAc in pet ether 40–60) gave the product as a cream-colored solid (186 mg, 64%). ^1^H NMR (400 MHz, Chloroform-*d*) δ 8.58 (s, 1H), 8.01 (d, *J* = 2.2 Hz, 1H), 7.88 (dd, *J* = 8.4, 2.2 Hz, 1H), 7.80 (s, 1H), 7.64 (d, *J* = 8.4 Hz, 1H), 7.49 (dd, *J* = 2.9 Hz, 1H), 6.68 (dd, *J* = 3.2, 2.0 Hz, 1H), 3.97 (s, 3H), 2.87 (s, 3H). LCMS retention time = 2.33 min (100%), (*m*/*z*) [M – H]^−^ = 323.

### 4-(4-Chloro-3-cyanophenyl)-7-methyl-1*H*-indole-6-carboxylic Acid **6**

Methyl ester **20** (99 mg, 0.30 mmol) was deprotected according to Method C to give the product as a cream-colored solid (88 mg, 93%). ^1^H NMR (400 MHz, Methanol-*d*_4_) δ 8.09 (d, *J* = 2.2 Hz, 1H), 7.99 (dd, *J* = 8.5, 2.2 Hz, 1H), 7.80–7.73 (m, 2H), 7.55 (d, *J* = 3.1 Hz, 1H), 6.66 (d, *J* = 3.2 Hz, 1H), 2.86 (s, 3H). LCMS retention time = 2.04 min (98%), (*m*/*z*) [M – H]^−^ = 309.

### Methyl 4-(4-Chloro-3-cyanophenyl)-1*H*-indole-6-carboxylate **21**

Methyl 4-bromo-1*H*-indol-6-carboxylate (100 mg, 0.39 mmol) and 4-chloro-3-cyanophenylboronic acid (79 mg, 0.43 mmol, 1.1 equiv) were reacted according to Method A. Purification by FC (SiO_2_, 8–66% EtOAc in pet ether 40–60), then trituration with CH_2_Cl_2_, gave the product as an off-white solid (77 mg, 63%). ^1^H NMR (400 MHz, Chloroform-*d*) δ 8.65 (s, 1H), 8.24 (d, *J* = 1.3 Hz, 1H), 8.04 (d, *J* = 2.1 Hz, 1H), 7.93–7.84 (m, 2H), 7.66 (d, *J* = 8.4 Hz, 1H), 7.51 (t, *J* = 2.9 Hz, 1H), 6.69 (t, *J* = 2.5 Hz, 1H), 3.99 (s, 3H). LCMS: retention time = 2.31 min (97%), *m*/*z* (ES−) 309 ([M – H]^−^, 100%).

### 4-(4-Chloro-3-cyanophenyl)-1*H*-indole-6-carboxylic Acid **5**

Methyl ester **21** (16 mg, 0.05 mmol) was hydrolyzed with NaOH (6 mg, 0.15 mmol, 3 equiv) and purified according to Method B to give the product as a cream-colored solid (12 mg, 79%). NMR: ^1^H NMR (400 MHz, DMSO-*d*_6_) δ 12.68 (s, 1H), 11.77 (s, 1H), 8.24 (d, *J* = 2.1 Hz, 1H), 8.13 (s, 1H), 8.05 (dd, *J* = 8.5, 2.2 Hz, 1H), 7.87 (d, *J* = 8.5 Hz, 1H), 7.77–7.69 (m, 2H), 6.67 (t, *J* = 2.3 Hz, 1H). LCMS: retention time = 2.02 min (98%), *m*/*z* (ES−) 295 ([M – H]^−^, 100%).

### Methyl 5-Hydroxy-4′-(trifluoromethoxy)-[1,1′-biphenyl]-3-carboxylate **22**

Methyl 3-hydroxy-5-(4,4,5,5-tetramethyl-1,3,2-dioxaborolan-2-yl)benzoate (42 mg, 0.15 mmol) and 1-bromo-4-(trifluoromethoxy)benzene (36 mg, 0.15 mmol) were reacted and purified according to Method A, to give the product as a white solid (37 mg, 78%). ^1^H NMR (400 MHz, Chloroform-*d*) δ 7.84 (t, *J* = 1.5 Hz, 1H), 7.64–7.58 (m, 3H), 7.33–7.26 (m, 3H), 3.97 (s, 3H).

### 5-Hydroxy-4′-(trifluoromethoxy)-[1,1′-biphenyl]-3-carboxylic Acid **2**

Methyl ester **22** (37 mg, 0.12 mmol) was hydrolyzed with LiOH monohydrate (15 mg, 0.36 mmol) and purified according to Method B to give the product as a white solid (24 mg, 68%). ^1^H NMR (400 MHz, Methanol-*d*_4_) δ 7.76 (t, *J* = 1.6 Hz, 1H), 7.74–7.70 (m, 2H), 7.47 (dd, *J* = 2.4, 1.4 Hz, 1H), 7.38 (d, *J* = 8.2 Hz, 2H), 7.27 (t, *J* = 2.1 Hz, 1H). LCMS retention time = 2.06 min, *m*/*z* = 297.1 (M – H)^−^.

### Methyl 4-(4-Chlorophenyl)-1*H*-indole-6-carboxylate **23**

Methyl 4-bromo-1*H*-indol-6-carboxylate (200 mg, 0.79 mmol) and 4-chlorophenylboronic acid (148 mg, 0.94 mmol, 1.2 equiv) were reacted according to Method A. Purification by FC (SiO_2_, 6–50% EtOAc in pet ether 40–60) gave the product as a pale yellow solid (193 mg, 86%). ^1^H NMR (400 MHz, Chloroform-*d*) δ 8.56 (s, 1H), 8.19 (d, *J* = 1.2 Hz, 1H), 7.88 (d, *J* = 1.3 Hz, 1H), 7.71–7.62 (m, 2H), 7.53–7.43 (m, 3H), 6.74 (t, *J* = 2.9 Hz, 1H), 3.98 (s, 3H). LCMS retention time = 2.37 min (95%), *m*/*z* (ES−) 284 ([M – H]^−^, 100%).

### 4-(4-Chlorophenyl)-1*H*-indole-6-carboxylic Acid **3**

Methyl ester **23** (189 mg, 0.66 mmol) was hydrolyzed with NaOH (79 mg, 1.98 mmol, 3 equiv) according to Method B to give the product as a pale yellow solid (175 mg, 97%). ^1^H NMR (400 MHz, Methanol-*d*_4_) δ 8.17 (t, *J* = 1.2 Hz, 1H), 7.79 (d, *J* = 1.4 Hz, 1H), 7.75–7.66 (m, 2H), 7.56–7.48 (m, 3H), 6.67 (dd, *J* = 3.2, 0.9 Hz, 1H). LCMS retention time = 2.11 min (100%), *m*/*z* (ES−) = 270 ([M – H]^−^, 100%).

## Supporting Information Available

A file with detailed experimental methods and supporting figures and tables (PDF)
A file with compound SMILES strings (CSV)

## Accession Codes

Protein Data Bank accession codes: 8C1M (**2**), 8C15 (**3**), 8C1D (**4**), 8C1E (**5**), 8C1F (**6**), 8C1G (**7**), 8C1H (**8**), 8C14 (**9**), 8C1I (**10**), and 8C1K (**CAM2602**). Authors will release the atomic coordinates and experimental data upon article publication.
