# Supplementary material for: Selective Aurora A-TPX2 Interaction Inhibitors Have In Vivo Efficacy as Targeted Antimitotic Agents
Source: J Med Chem. 2024 Aug 27;67(17):15521–36. doi: 10.1021/acs.jmedchem.4c01165 (PMC11403621; doi:10.1021/acs.jmedchem.4c01165)
Supplement: Supplementary file 1 — jm4c01165_si_001.pdf [file jm4c01165_si_001.pdf]

Supporting Information
Selective Aurora A-TPX2 interaction inhibitors have in vivo efficacy as targeted anti-mitotic agents

Simon R. Stockwell[1,4#], Duncan E. Scott[2,5#*], Gerhard Fischer[3] , Estrella Guarino[1,6], Timothy P. C. Rooney[2,7], Tzu-Shean Feng[2], Tommaso Moschetti[3,8], Rajavel Srinivasan[2,9], Esther Alza[2,10], Alice Asteian[2,11], Claudio Dagostin[2,4], Anna Alcaide[2], Mathieu Rocaboy[3], Beata Blaszczyk[3], Alicia Higueruelo[3,12], Xuelu Wang[3,13], Maxim Rossmann[3,14], Trevor R. Perrior[15] Tom L. Blundell[3,16], David R. Spring[2], Grahame McKenzie[1,17], Chris Abell[2¶], John Skidmore[2,7*], Ashok R. Venkitaraman[1,18*], Marko Hyvönen[3*]

[1] Medical Research Council Cancer Unit, University of Cambridge, Cambridge CB2 0XZ, UK
[2] Yusuf Hamied Department of Chemistry, University of Cambridge, Cambridge, CB2 1EW, UK
[3] Department of Biochemistry, University of Cambridge, Cambridge CB2 1GA, UK

*Correspondence to:
DES (chemistry), DScott004@dundee.ac.uk
JS (drug discovery), js930@cam.ac.uk
ARV (biology): arv22@nus.edu.sg
MH (biochemistry), mh256@cam.ac.uk

[#] These authors contributed equally
[¶] Deceased
[4] Current address: o2h Discovery, Cambridge, UK
[5] Current address: Drug Discovery Unit, School of Life Sciences, University of Dundee, Dundee, UK
[6] Current address: Spanish National Cancer Research Center (CNIO), Madrid, Spain
[7] Current address: The ALBORADA Drug Discovery Institute, University of Cambridge, Cambridge, UK
[8] Current address: Research & Development, Illumina Cambridge Ltd, Cambridge, UK
[9] Current address: School of Pharmaceutical Science and Technology, Tianjin University, Tianjin, People's Republic of China and Singapore Eye Research Institute, The Academia, Singapore
[10] Current address: Alza & Associates S.L. Sustainable Chemistry, Barcelona, Spain
[11] Current address: Beckman Coulter, Marseille, France
[12] Current address: Exscientia, Oxford, UK
[13] Current address: AstraZeneca, Cambridge, UK
[14] Current address: Cambridge Institute for Medical Research, University of Cambridge, Cambridge, UK
[15] Excellium Consulting, Bury St Edmunds,UK
[16] Current address: Heart and Lung Research Institute, University of Cambridge, Cambridge, UK
[17] Current address: Mosaic Therapeutics, Cambridge, UK
[18] Current address: Cancer Science Institute of Singapore, National University of Singapore, Centre for Translational Medicine, Singapore; Institute of Molecular & Cell Biology (IMCB), Agency for Science, Technology & Research (A*STAR), Singapore

Contents of SI

Supporting Information Selective Aurora A-TPX2 interaction inhibitors have in vivo efficacy as targeted anti-mitotic agents 1

Biophysical, cellular and in vivo analyses........ 4

Figure S1. LO-NMR screening and Fluorescence polarisation anisotropy assay. 4

Figure S2. ITC analysis of selectivity for Aurora A vs. Aurora B for compounds 7,8, and 9. 5

Figure S3. DiscoverX KINOMEscan specificity screen of compound 9. 6

Table S1. High content toxicology analysis. 7

Table S2. Cerep Express Profile screen data. 7

Table S3. Calculated and measured ADMET properties for CAM2602 9

Figure S4. Mitotic spindle abnormalities in cells treated with 6. 10

Figure S5. Toxicity in non-cycling cells. 10

Figure S6. PH3 levels evaluation with compound 7. 11

Figure S7. Flow cytometry gating strategy to detect Aurora A inhibition biomarkers. 11

Figure S8. Pharmacokinetics of CAM2602. 12

Figure S9. Tolerability of CAM2602. 12

Figure S10. Analysis of blood samples upon conclusion of efficacy study. 13

Figure S11. Aurora A:TPX2 PPI inhibitors synergise with paclitaxel in PANC-1 cells. 14

Table S4. Crystallographic data collection and refinement parameters. 15

Figure S12. Electron densities of ligands 19

Figure S13. High-content assay to assess cellular Aurora A engagement by PPI inhibitors. 20

Chemical structures of key compounds 21

Figure S14 Chemical structures of key compounds 21

Synthetic Chemistry 21

Scheme S1 Synthesis of compound 2, 3 and 5 21

Scheme S2 Synthesis of compound 10 and CAM2602 22

Scheme S3 Synthesis of compound 4, 7 and 8 22

Scheme S4 Synthesis of compound 18 and 9 23

Scheme S5 Synthesis of compound 6 23

Final compound characterisation spectra 24

Figure S15 ^{1}H NMR of compound 2 24

Figure S16 ^{1}H NMR of compound 3 25

Figure S17 ^{1}H NMR of compound 4 26

Figure S18 ^{1}H NMR of compound 5 27

Figure S19 ^{1}H NMR of compound 6 28

Figure S20 ^{1}H NMR of compound 7 29

Figure S21 ^{1}H NMR of compound 8 30
Figure S22 ^{1}H NMR of compound 9 31
Figure S23 ^{1}H NMR of compound 10 32
Figure S24 ^{1}H NMR of CAM2602 33
Figure S25. HPLC analysis of 2.. 34
Figure S26. HPLC analysis of 3.. 34
Figure S27. HPLC analysis of 4.. 34
Figure S28. HPLC analysis of 5.. 34
Figure S29. HPLC analysis of 6.. 35
Figure S30. HPLC analysis of 7.. 35
Figure S31. HPLC analysis of 8.. 35
Figure S32. HPLC analysis of 9.. 35
Figure S33. HPLC analysis of 10.. 36
Figure S34. HPLC analysis of CAM2602.. 36

Biophysical, cellular and in vivo analyses

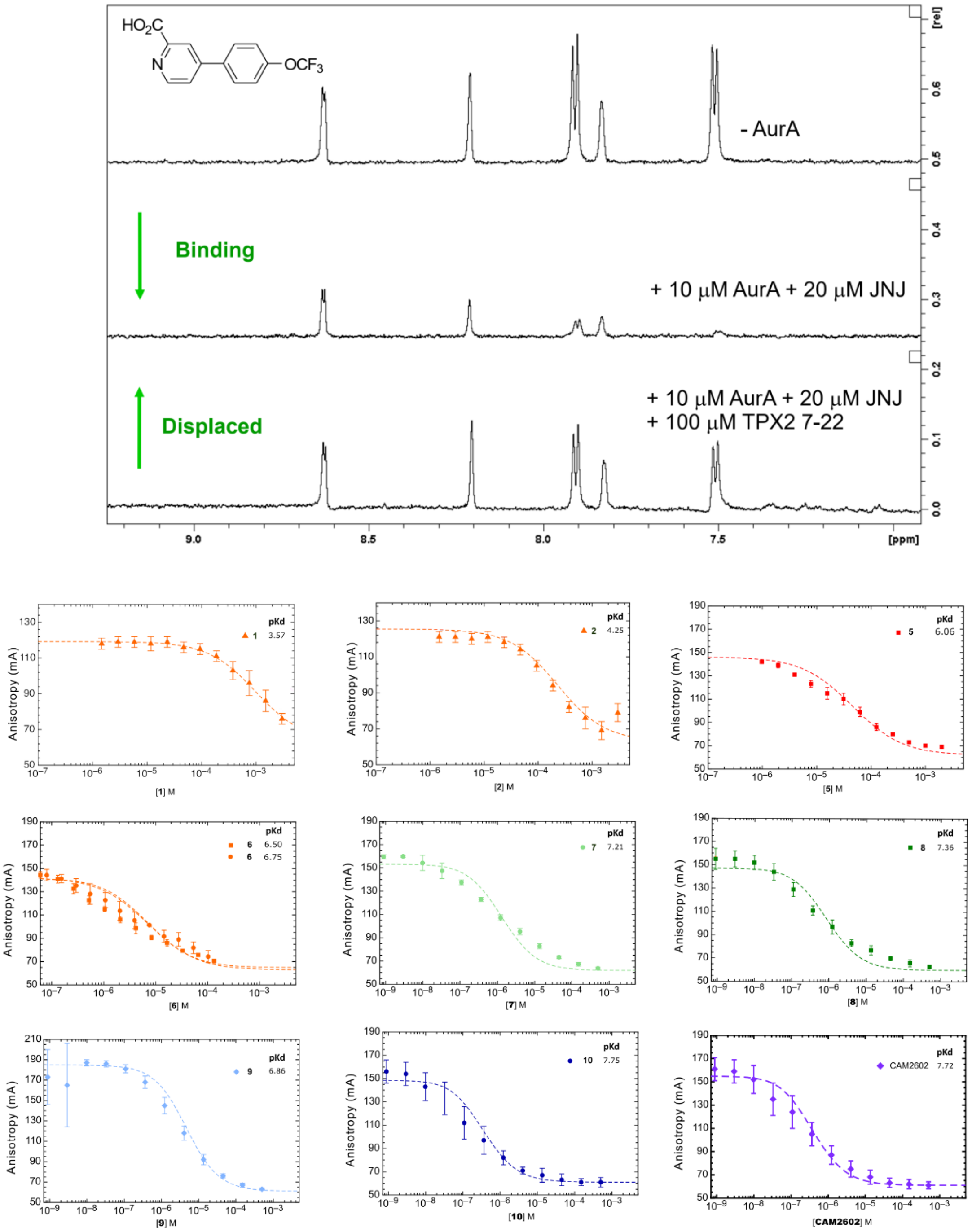

Figure S1. LO-NMR screening and Fluorescence polarisation anisotropy assay.
Top panel: CPMG ligand-observed NMR experiment with analogue of compound **2**. Top: 1D spectrum of the compound. Middle: CPMG experiment in the presence of Aurora A and ATP-site binding compound JNJ-7706621. Bottom: As in the middle but with competition with Tyr-pocket binding TPX2 peptide comprising of residues 7-22.

Bottom panels: Competitive FP assay with selected compounds. AlexaFluor 488-labelled TPX2 peptide was displaced from Aurora A by increasing concentration of inhibitors. pK_D values in each graph represent values from individual experiments in display, while K_D values in Figure 2 are averages from 2-3 replicates.

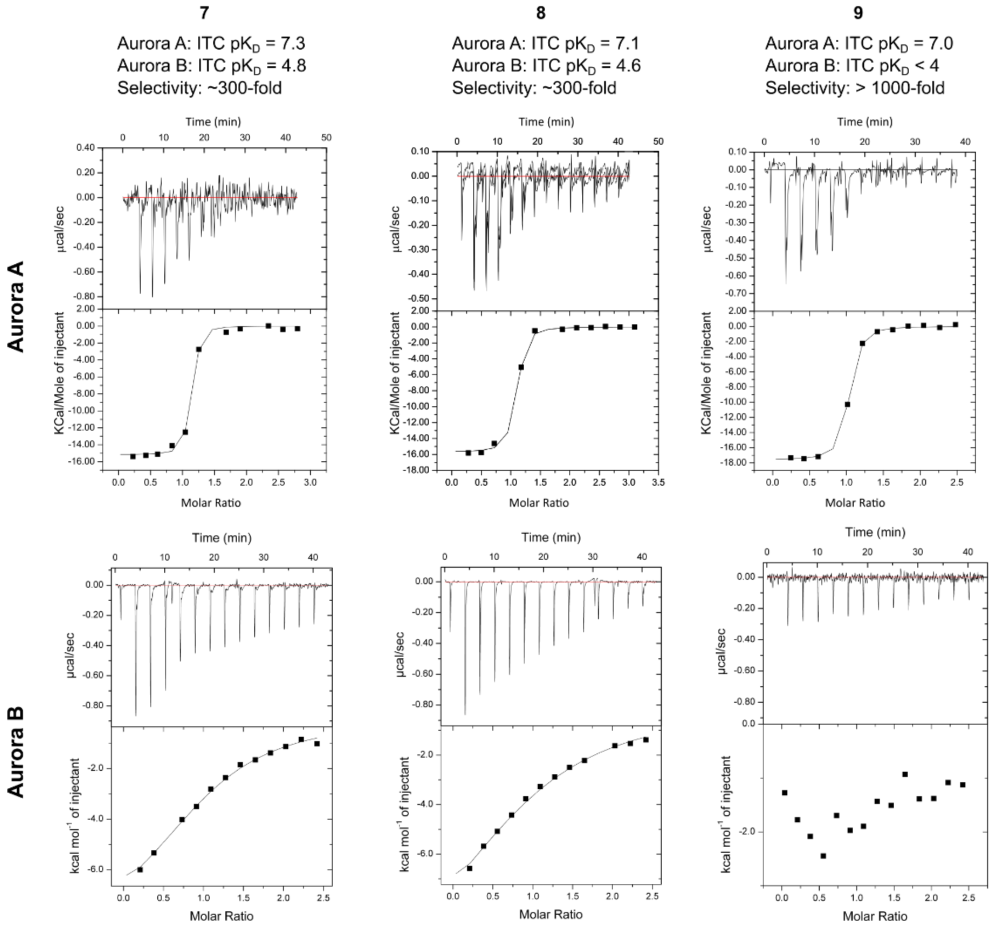

Figure S2. ITC analysis of selectivity for Aurora A vs. Aurora B for compounds 7,8, and 9.

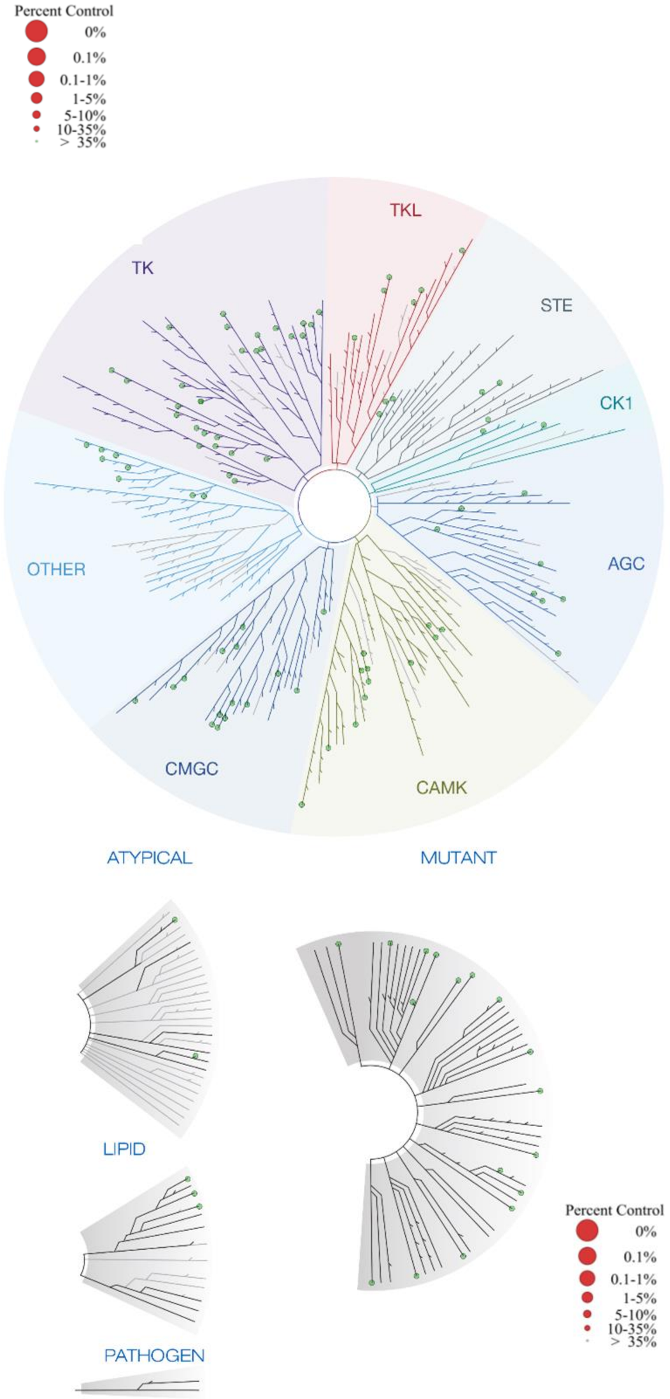

Target	8
Gene Symbol	%Ctrl @ 10000nM
ABL1(E255K)-phosphorylated	100
ABL1(T315I)-phosphorylated	100
ABL1-nonphosphorylated	99
ABL1-phosphorylated	100
ACVR1B	100
ADCK3	99
AKT1	100
AKT2	100
ALK	100
AURKA	71
AURKB	100
AXL	93
BMPR2	100
BRAF	100
BRAF(V600E)	100
BTK	94
CDK11	93
CDK2	95
CDK3	100
CDK7	98
CDK9	81
CHEK1	94
CSF1R	99
CSNK1D	100
CSNK1G2	91
DCAMKL1	96
DYRK1B	97
EGFR	99
EGFR(L858R)	100
EPHA2	99
ERBB2	100
ERBB4	97
ERK1	87
FAK	100
FGFR2	94
FGFR3	96
FLT3	100
GSK3B	88
IGF1R	79
IKK-alpha	100
IKK-beta	100
INSR	100
JAK2(JH1domain-catalytic)	94
JAK3(JH1domain-catalytic)	98
JNK1	93
JNK2	83
JNK3	80
KIT	100

Target	8
Gene Symbol	%Ctrl @ 10000nM
KIT(D816V)	93
KIT(V559D,T670I)	98
LKB1	95
MAP3K4	85
MAPKAPK2	89
MARK3	100
MEK1	98
MEK2	100
MET	91
MKNK1	98
MKNK2	95
MLK1	86
p38-alpha	97
p38-beta	100
PAK1	85
PAK2	52
PAK4	93
PCTK1	100
PDGFRA	100
PDGFRB	100
PDPK1	96
PIK3C2B	97
PIK3CA	100
PIK3CG	100
PIM1	94
PIM2	98
PIM3	98
PKAC-alpha	86
PLK1	96
PLK3	91
PLK4	100
PRKCE	99
RAF1	100
RET	94
RIOK2	100
ROCK2	100
RSK2(Kin.Dom.1-N-terminal)	95
SNARK	95
SRC	79
SRPK3	100
TGFBR1	100
TIE2	100
TRKA	85
TSSK1B	74
TYK2(JH1domain-catalytic)	100
ULK2	100
VEGFR2	100
YANK3	92
ZAP70	100

Figure S3. DiscoverX KINOMEscan specificity screen of compound 9.
Compound **9** was applied at 10 µM to proprietary cell-free assays, which estimate kinase target inhibition as a product of blocking association with immobilised ATP. (**Left**) TREEspot™ plots of the 97 kinases in the screen organised by kinase family where circles indicate target dendrogram position and size/colour indicate degree of inhibition. (**Right**) List of the 97 kinases screened alongside percent-inhibition detected.

Table S1. High content toxicology analysis.

Cell health parameter	Change	MEC (μM)	AC_{50} (μM)	First signal MEC	First signal AC_{50}
Cell count	↓	60.7	>100		•
Nuclear size	↑	45.5	>100		•
DNA structure	↑	29.9	>100		•
Cell membrane permeability	↑	10.1	>100	•	•
Mitochondrial mass		NR	NR		
Mitochondrial membrane potential		NR	NR		
Cytochrome c	↑	15.3	>100		•

Summary of high content toxicology from 72h dose response experiment up to 100 μM of **7** on HepG3 cells. MEC: Minimum effective concentration that significantly crosses vehicle control threshold; AC_{50}: The concentration at which 50% maximum effect is observed for each cell health parameter. First Signal: the cell health feature which responds at the lowest observed dose (marked by •). NR: No response observed.

Table S2. Cerep Express Profile screen data.

CEREP panel data	7 at 10 μM	CAM2602 at 10 μM
Assay	% Inhibition of Control Specific Binding	% Inhibition of Control Specific Binding
A1 (h) (antagonist radioligand)	-11	4
A2A (h) (agonist radioligand)	-16	-17
A3 (h) (agonist radioligand)	25	55
alpha 1 (non-selective) (antagonist radioligand)	4	-1
alpha 2 (non-selective) (antagonist radioligand)	-3	-19
beta 1 (h) (agonist radioligand)	5	6
beta 2 (h) (agonist radioligand)	6	0
AT1 (h) (antagonist radioligand)	12	5
BZD (central) (agonist radioligand)	-2	-14
B2 (h) (agonist radioligand)	-8	-73
CB1 (h) (agonist radioligand)	-7	-26
CCK1 (CCKA) (h) (agonist radioligand)	-24	-48
D1 (h) (antagonist radioligand)	-1	2
D2S (h) (antagonist radioligand)	2	4
ETA (h) (agonist radioligand)	16	21
GABA (non-selective) (agonist radioligand)	-17	-23
GAL2 (h) (agonist radioligand)	1	-1
CXCR2 (IL-8B) (h) (agonist radioligand)	-6	1
CCR1 (h) (agonist radioligand)	-8	-8
H1 (h) (antagonist radioligand)	4	-7
H2 (h) (antagonist radioligand)	-6	-3
MC4 (h) (agonist radioligand)	-1	-3
MT1 (ML1A) (h) (agonist radioligand)	0	0
M1 (h) (antagonist radioligand)	1	7

M2 (h) (antagonist radioligand)	-8	-21
M3 (h) (antagonist radioligand)	7	-4
NK2 (h) (agonist radioligand)	0	-5
NK3 (h) (antagonist radioligand)	-3	-20
Y1 (h) (agonist radioligand)	4	-1
Y2 (h) (agonist radioligand)	-1	-6
NTS1 (NT1) (h) (agonist radioligand)	-19	-1
delta (DOP) (h) (agonist radioligand)	2	14
kappa (KOP) (agonist radioligand)	7	7
mu (MOP) (h) (agonist radioligand)	1	5
NOP (ORL1) (h) (agonist radioligand)	17	0
EP4 (h) (agonist radioligand)	14	4
5-HT1A (h) (agonist radioligand)	-4	20
5-HT1B (antagonist radioligand)	1	-1
5-HT2A (h) (antagonist radioligand)	0	-11
5-HT2B (h) (agonist radioligand)	18	22
5-HT3 (h) (antagonist radioligand)	9	7
5-HT5a (h) (agonist radioligand)	12	0
5-HT6 (h) (agonist radioligand)	3	0
5-HT7 (h) (agonist radioligand)	11	3
sst (non-selective) (agonist radioligand)	2	1
VPAC1 (VIP1) (h) (agonist radioligand)	-7	-7
V1a (h) (agonist radioligand)	4	3
Ca2+ channel (L, verapamil site) (phenylalkylamine) (antagonist radioligand)	-12	-5
KV channel (antagonist radioligand)	0	-3
SKCa channel (antagonist radioligand)	3	-2
Na+ channel (site 2) (antagonist radioligand)	-10	-5
Cl- channel (GABA-gated) (antagonist radioligand)	13	28
norepinephrine transporter (h) (antagonist radioligand)	-2	6
dopamine transporter (h) (antagonist radioligand)	13	11
5-HT transporter (h) (antagonist radioligand)	-9	-5

7 and **CAM2602** were screened at 10 µM against a panel of 55 GPCRs, transporters and ion channels. The percentage inhibition of the binding of a radioactively labelled ligand specific for each target is given.

Table S3. Calculated and measured ADMET properties for CAM2602

Property	Value
MW	483.97 Da
TPSA	96.9 Å^2
LogP[a]	4.38
pK_D (FP)	7.4
Microsomal CLint	Mouse $t_{1/2}$ = 106 min<br>13.1 µL/min/mg protein<br>Human $t_{1/2}$ = 155 min<br>8.9 µL/min/mg protein
Hepatocyte CLint	Mouse $t_{1/2}$ = 139 min<br>10 µL/min/10^{-6} cells<br>Rat $t_{1/2}$ = 408 min<br>3.4 µL/min/10^{-6} cells<br>Human $t_{1/2}$ = 205 min<br>6.8 µL/min/10^{-6} cells
hERG inhibition IC_{50}[b]	>25 µM
Mouse plasma protein binding	98.5%
Human plasma protein binding	99.6%
Caco-2 permeability:	
Papp (10^{-6} cm s^{-1}) A2B / B2A / Efflux ratio	5.1 / 41 / 8.1
CYP450 IC_{50}[c]	All >25 µM

[a] Calculated with ChemDraw version 19.1.0.8

[b] Whole-cell voltage clamping assay with mammalian cells transfected with hERG potassium channel

[c] CYP1A (ethoxyresorufin), CYP2C9 (tolbutamide), CYP219 (mephenytoin), CYP2D6 (dextromethorphan), CYP3A4 (midazolam), CYP3A4 (testosterone).

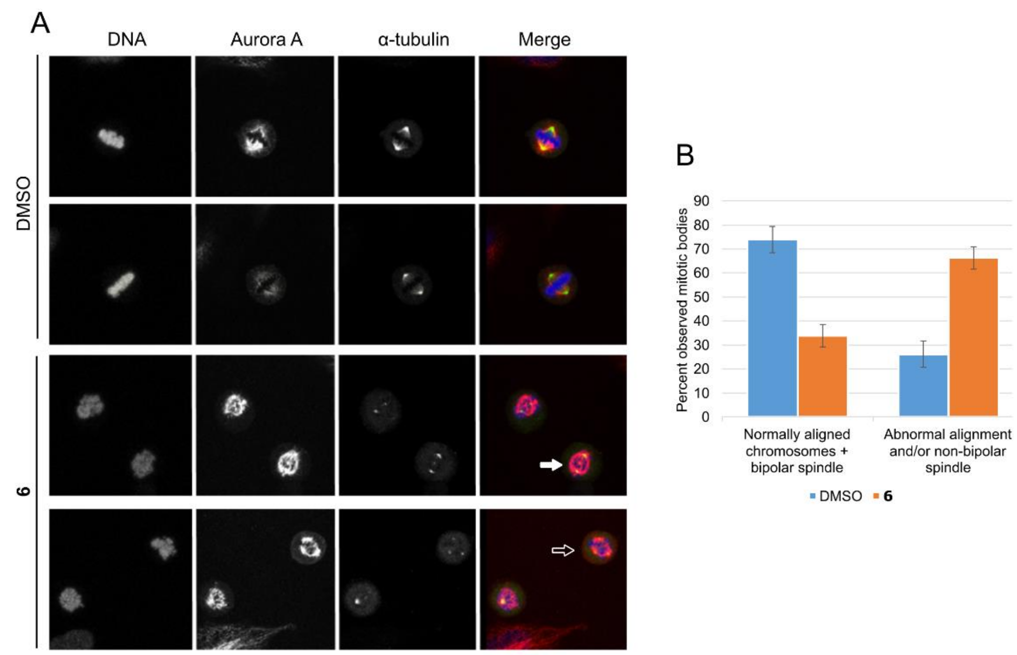

Figure S4. Mitotic spindle abnormalities in cells treated with 6.
(**A**) HeLa cells were treated with 50 µM (1x GI_{50}) **6** or DMSO for 6 hours prior to being fixed, fluorescently stained for the indicated proteins or DNA and imaged using confocal microscopy. Two representative fields containing mitotic cells are shown for both treatment conditions. Mitotic cells were enumerated to exhibit spindle abnormalities if they demonstrated unaligned chromosomes and/or non-bipolarity, examples of which are indicated by the solid and outline arrowhead, respectively. (**B**) Relative proportions of normal and abnormal spindle classes across all imaged mitotic cells for both DMSO and **5** treated cells (>100 mitotic cell observations). Error bars show standard deviations from the mean (n=3 image sets per condition).

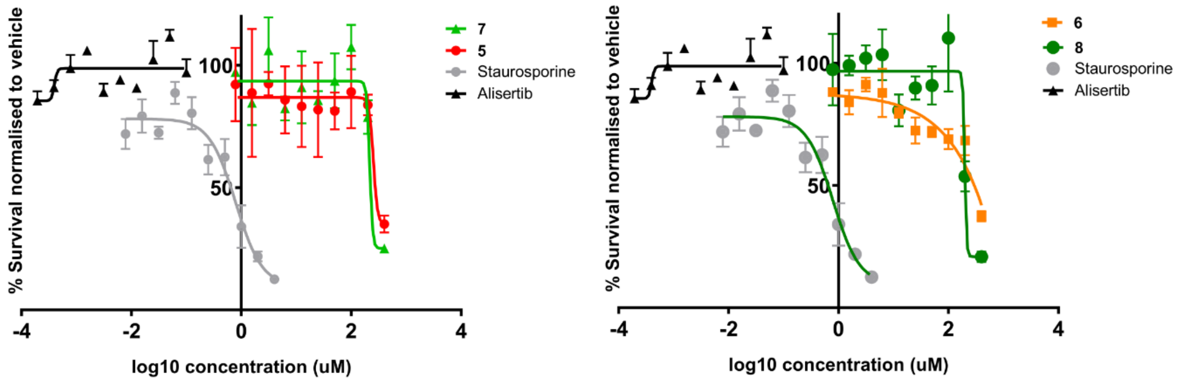

Figure S5. Toxicity in non-cycling cells.
Viable, non-cycling peripheral blood mononuclear cells were grown for 72 hours in the presence of increasing concentrations of the indicated compounds, Alisertib or staurosporine as a positive control. After 72 hours the cell media was supplemented with CellTiter Blue dye to fluorescently quantify the viable cells under each treatment condition. Viability values were normalised against concurrently performed vehicle controls (DMSO for CAM and Alisertib, EtOAc for staurosporine). Concentrations of compounds **5-8;** 0.8-400 uM; Alisertib 0.2-100 nM; staurosporine 8 nM- 4 uM.

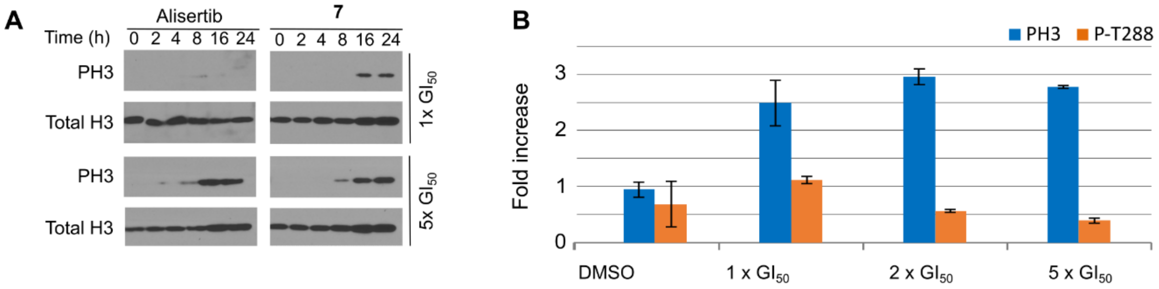

Figure S6. PH3 levels evaluation with compound 7.
(A) Western blot analysis of PH3 levels in Jurkat cells treated with the indicated fold-GI_{50} equivalents of alisertib (7 or 35 nM) and **7** (20 or 100 µM). **(B)** Flow cytometric analysis of Jurkat cells treated with a range of fold-GI_{50} concentrations of **7** (1x GI_{50} = 20 µM) for 8 h. The cells were stained for DNA, PH3 and P-Thr288 Aurora A and were analysed to determine the proportion of mitotic cells (having both 4n DNA and PH3 positivity); additionally, the proportion of cells positive for P-Thr288 within the mitotic population was also measured per treatment condition. Data is plotted as normalised values relative to the untreated control.

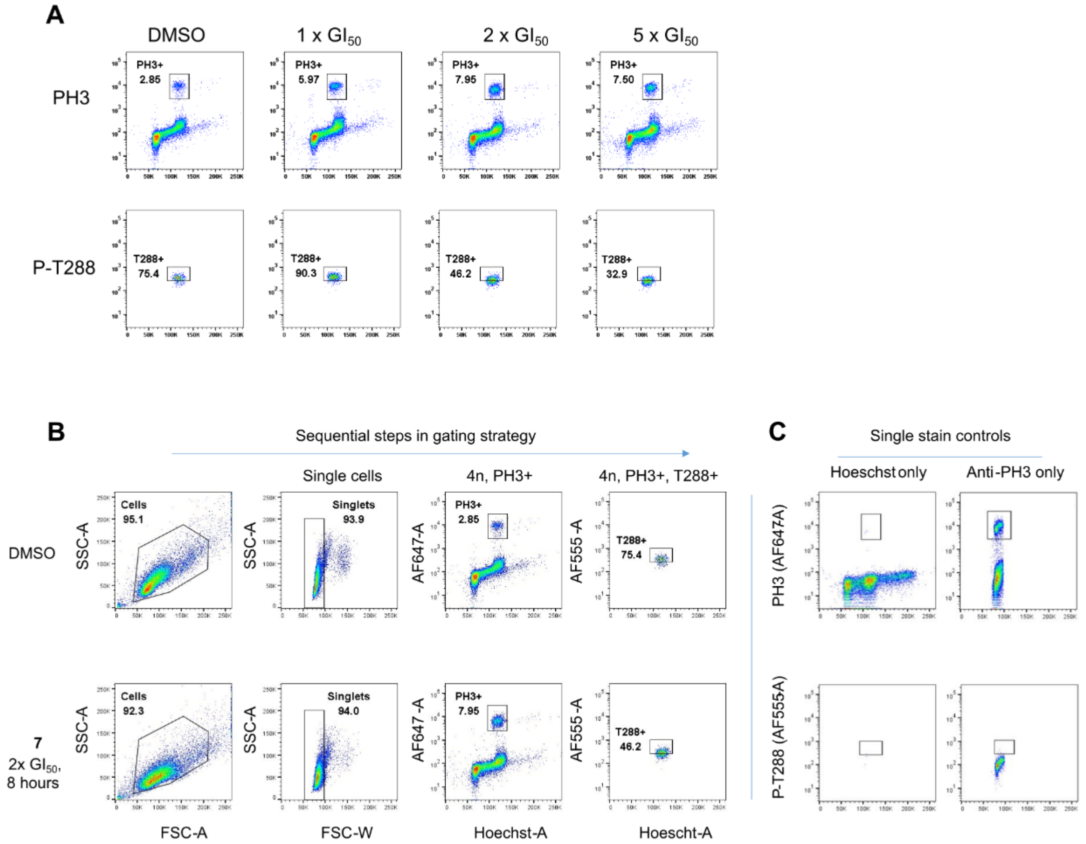

Figure S7. Flow cytometry gating strategy to detect Aurora A inhibition biomarkers.
(A) Flow cytometric data as summarised in main **Fig. 5**. (B) Flow cytometric analysis of Jurkat cells treated for 8 hours with either DMSO or 2x GI_{50} concentrations of **7**. From left to right, the four scatter plots show the sequential analysis used to detect the Aurora A biomarker changes resulting from the compound treatment applied using flow cytometry. Each scatter plot represents only those cells within the gated population (box) of the previous plot to the left. The leftmost two panels

concern the gating of non-debris, single cells in the samples through light scattering properties. The third panel plots these singlets against Hoechst-staining intensity of DNA (X-axis) and Alexa Fluor-labelled anti-phospho-histone 3 (PH3) antibody intensity (Y-axis). The gated population is both PH3-positive and possess 4n DNA, as expected from mitotic cells. In the final panel, the PH3+,4n cells are gated if positive for T288-phosphorylated (P-T288) Aurora A. (C) Positions of the gates for PH3 and P-T288 positivity were determined in untreated, single stain samples of cells.

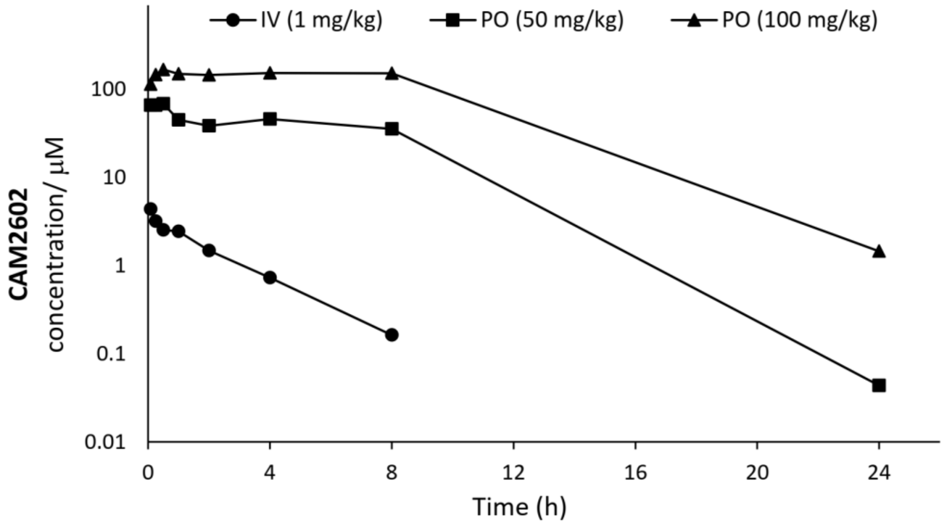

Figure S8. Pharmacokinetics of CAM2602.
CAM2602 was administered at three separate doses in female CD-1 mice and measuring the total concentration of compound in plasma over time.

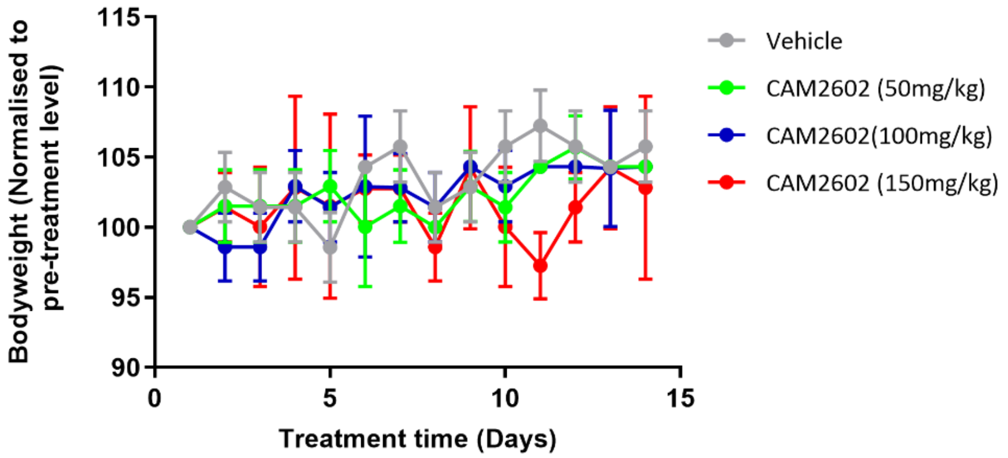

Figure S9. Tolerability of CAM2602.
The indicated doses of CAM2602 were orally administered to female NSG mice, QD for 7 days, followed by 7 days with no dosing. Plotted are normalised bodyweight measurements taken daily. Values shown are mean ±SD; n=3 for all groups. Compound formulation: DMSO:20% HP-β-CD (2-hydroxypropyl-beta-cyclodextrin) in PBS (2.5:97.5) pH 7.6. At all doses, no ill-health indications were observed.

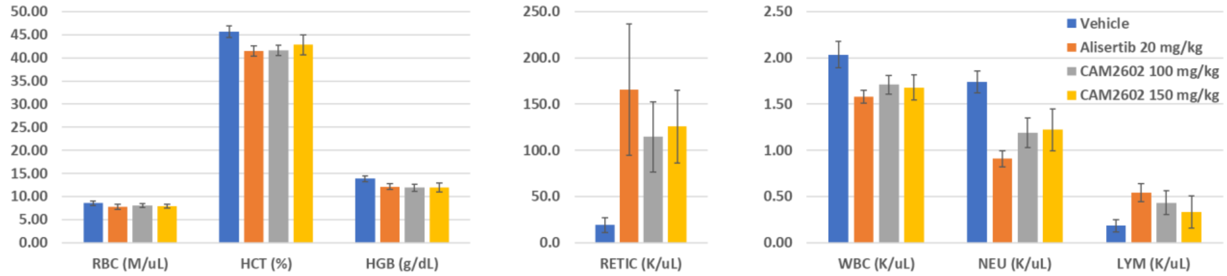

Figure S10. Analysis of blood samples upon conclusion of efficacy study.

On the final day of the study, blood samples from each mouse were assessed to determine relative population sizes of each blood cell type per sample. Units are shown adjacent to categories: absolute counts (millions or thousands per microliter, M/µl, K/µl) or weight per volume (g/dL) or percentage parent population, where indicated. Categories: red blood cells (RBC); haematocrit (HCT); haemoglobin (HGB); reticulocytes (RETIC); white blood cells (WBC); neutrophils (NEU); lymphocytes (LYM). Values are means and standard deviations taken from 5 biological replicates.

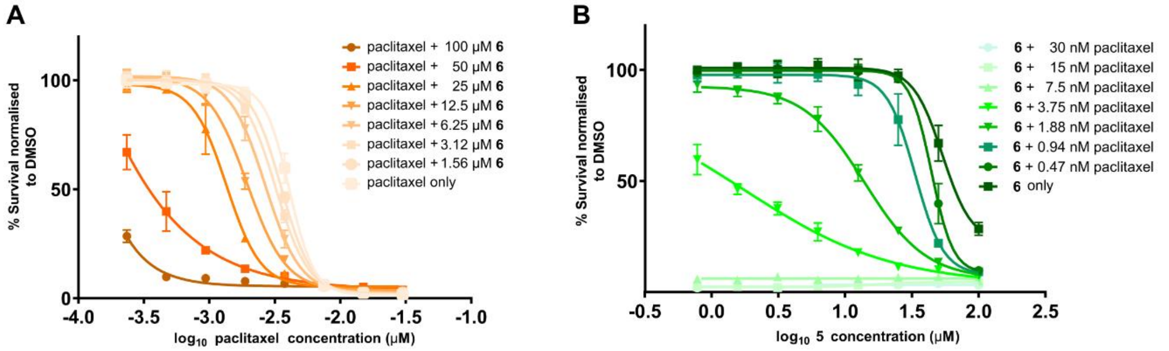

C

paclitaxel :	0 nM	1.88 nM	3.8 nM
**5** GI_{50} (μM):	54	14	1.8
Viability (Taxol only):	100%	94%	60%

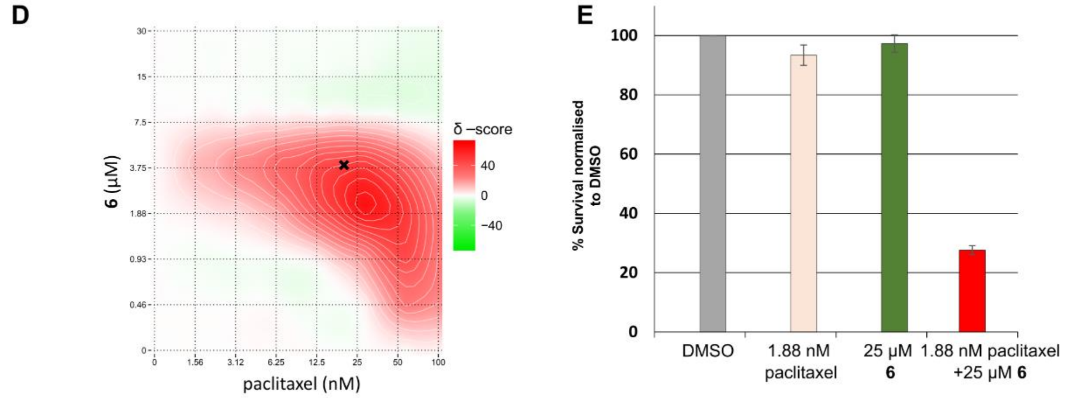

Figure S11. Aurora A:TPX2 PPI inhibitors synergise with paclitaxel in PANC-1 cells.
(A) and **(B)**: PANC-1 cells were dosed with a matrix of concentrations of Paclitaxel and **6**, including single agent and vehicle controls for all concentrations tested. 72 hours following treatment, the cells were assayed for remaining viability relative to vehicle controls. **(C)** Table showing effective decrease in **6** GI_{50} in PANC-1 cells when combined with increasing concentrations of paclitaxel. Also shown are the corresponding viability changes effected by paclitaxel if applied as a single agent. (**D**) The vehicle-normalised viability assay data were processed using SynergyFinder webserver (https://synergyfinder.org/)[57], producing a heatmap indicating the presence of synergy (red) or antagonism (green) between the two drugging agents when compared to modelled predictions of additivity (**E**) Chart comparing vehicle-normalised 72-hour viability assay values between single agent and combined treatments of the concentrations of paclitaxel and **6** yielding the greatest synergic effect. The single-agent inhibition values for paclitaxel alone or **6** alone were used to calculate a drug combination surface under the assumption of an additive effect using SynergyFinder, which is shown as the 'predicted' value. Bars show standard deviations from the mean (n=4).

Table S4. Crystallographic data collection and refinement parameters.

**Ligand**	**2**	**3**	**4**
PDB code	8C1M	8C15	8C1D
**Data Collection:**			
Beamline	DLS I04-1	ESRF ID29	ESRF MASSIF-3
Wavelength (Å)	0.92	0.973	0.9677
Resolution range (Å)	2.84-53.97 (2.91-2.84)	70.02 - 2.41 (2.416 - 2.407)	70.44 - 2.12 (2.122 - 2.115)
Space group	P 41 21 2	P 61 2 2	P 61 2 2
Cell (a b c) (Å)	82.82 82.82 139.0	80.86 80.86 165.16	81.34 81.34 171.53
Cell (α β γ) (°)	90.00 90.00 90.00	90.00 90.00 120.00	90.00 90.00 120.00
Total reflections	148180 (11280)	227396 (2326)	435735 (4149)
Unique reflections	11913 (847)	13083 (129)	19797 (178)
Multiplicity	12.4 (13.3)	17.4 (18.0)	22.0 (23.3)
Completeness (%)	100.0 (99.8)	100.0 (98.5)	99.2 (96.2)
Mean I/σ(I)	25.5 (3.7)	16.6 (1.6)	23.5 (1.3)
R_{merge}	0.071 (0.828)	0.103 (1.94)	0.069 (2.90)
R_{pim}	0.028 (0.323)	0.026 (0.46)	0.015 (0.61)
CC ½	1.000 (0.963)	0.999 (0.67)	0.999 (0.67)
**Refinement:**			
R / R_{free}	0.2529/0.2620	0.199 / 0.207	0.210 / 0.238
No. of atoms	2150	2251	2291
No of ligand atoms	50	102	103
No of waters	0	40	83
Number of protein residues	262	259	259
Average/Wilson B factor (Å^2)	82.353/72.37	75.6 / 74.1	67.0 / 61.4
B-factor for ligands (Å^2)	100.2	99.4	92.1
B-factor for solvent (Å^2)	n/a	71.1	68.9
RMS (bonds) (Å)	0.006	0.01	0.009
RMS (bond angles) (°)	0.87	1.08	1.03
RMS (dihedral angles) (°)	2.55	3.02	3.04

Ligand	5	6	7
PDB code	8C1E	8C1F	8C1G
**Data Collection:**			
Beamline	SOLEIL PROXIMA 2	ESRF MASSIF-3	DLS I03
Wavelength (Å)	0.9801	0.9677	0.97
Resolution range (Å)	135.87 - 2.80 (2.808 - 2.798)	65.26 - 1.92 (1.931 - 1.924)	53.33 - 1.96 (1.965 - 1.959)
Space group	P 41 21 2	P 61 2 2	P 61 2 2
Cell (a b c) (Å)	82.04 82.04 135.87	81.44 81.44 172.01	80.82 80.82 164.67
Cell (α β γ) (°)	90.00 90.00 90.00	90.00 90.00 120.00	90.00 90.00 120.00
Total reflections	83838 (974)	405731 (3313)	224440 (2256)
Unique reflections	12082 (135)	24878 (216)	22054 (234)
Multiplicity	6.9 (7.2)	16.3 (15.3)	10.2 (9.6)
Completeness (%)	100.0 (100.0)	94.2 (83.7)	93.2 (98.7)
Mean I/σ(I)	14.0 (3.4)	25.8 (1.7)	15.3 (1.7)
R_{merge}	0.167 (1.16)	0.057 (1.56)	0.075 (0.87)
R_{pim}	0.069 (0.47)	0.014 (0.41)	0.025 (0.29)
CC ½	0.994 (0.55)	1.000 (0.69)	0.999 (0.81)
**Refinement:**			
R / R_{free}	0.173 / 0.239	0.205 / 0.234	0.199 / 0.225
No. of atoms	2279	2329	2326
No of ligand atoms	54	131	120
No of waters	73	90	115
Number of protein residues	262	258	258
Average/Wilson B factor (Å^2)	48.8 / -	49.1 / 42.1	45.2 / 36.4
B-factor for ligands (Å^2)	41.3	71.5	59.5
B-factor for solvent (Å^2)	47.2	50.5	48.2
RMS (bonds) (Å)	0.015	0.01	0.01
RMS (bond angles) (°)	2.102	1.09	1.06
RMS (dihedral angles) (°)	7.153	3.53	3.24

Ligand	**8**	**9**	**10**
PDB code	8C1H	8C14	8C1I
**Data Collection:**			
Beamline	DLS I03	ESRF MASSIF-3	ESRF ID29
Wavelength (Å)	0.97	0.9677	0.973
Resolution range (Å)	64.61 - 2.23 (2.241 - 2.233)	81.04 - 1.93 (1.937 - 1.930)	83.63 - 2.81 (2.820 - 2.810)
Space group	P 61 2 2	P 41 21 2	P 61 2 2
Cell (a b c) (Å)	81.03 81.03 165.62	81.04 81.04 138.18	81.43 81.43 167.26
Cell (α β γ) (°)	90.00 90.00 120.00	90.00 90.00 90.00	90.00 90.00 120.00
Total reflections	184733 (1826)	418805 (4053)	149762 (1686)
Unique reflections	15901 (152)	32771 (332)	8604 (93)
Multiplicity	11.6 (12.0)	12.8 (12.2)	17.4 (18.1)
Completeness (%)	97.4 (98.7)	92.7 (100.0)	100.0 (98.9)
Mean I/σ(I)	13.6 (1.9)	15.2 (1.4)	17.0 (1.4)
R_{merge}	0.089 (0.98)	0.109 (2.23)	0.148 (1.91)
R_{pim}	0.027 (0.28)	0.032 (0.66)	0.036 (0.45)
CC ½	0.999 (0.86)	0.999 (0.60)	0.998 (0.72)
**Refinement:**			
R / R_{free}	0.211 / 0.245	0.181 / 0.194	0.212 / 0.234
No. of atoms	2294	2513	2249
No of ligand atoms	113	128	111
No of waters	77	205	14
Number of protein residues	258	264	260
Average/Wilson B factor (Å^2)	63.4 / 53.5	42.9 / 38.2	81.5 / 91.9
B-factor for ligands (Å^2)	77.3	67.7	103.7
B-factor for solvent (Å^2)	63.4	54.6	65.3
RMS (bonds) (Å)	0.011	0.01	0.008
RMS (bond angles) (°)	1.09	1.01	1.02
RMS (dihedral angles) (°)	3.28	3.34	3.01

Ligand	CAM2602
PDB code	8C1K
**Data Collection:**	
Beamline	DLS I24
Wavelength (Å)	0.9686
Resolution range (Å)	169.18 - 2.27 (2.304 - 2.265)
Space group	P 61 2 2
Cell (a b c) (Å)	82.14 82.14 169.18
Cell (α β γ) (°)	90.00 90.00 120.00
Total reflections	308579 (4402)
Unique reflections	12671 (190)
Multiplicity	24.4 (23.2)
Completeness (%)	92.5 (65.5)
Mean I/σ(I)	28.3 (1.2)
R_{merge}	0.074 (2.85)
R_{pim}	0.015 (0.60)
CC ½	1.000 (0.66)
**Refinement:**	
R / R_{free}	0.217 / 0.242
No. of atoms	2264
No of ligand atoms	115
No of waters	39
Number of protein residues	262
Average/Wilson B factor (Å^2)	82.2 / 82.1
B-factor for ligands (Å^2)	104.5
B-factor for solvent (Å^2)	74.9
RMS (bonds) (Å)	0.011
RMS (bond angles) (°)	1.25
RMS (dihedral angles) (°)	3.51

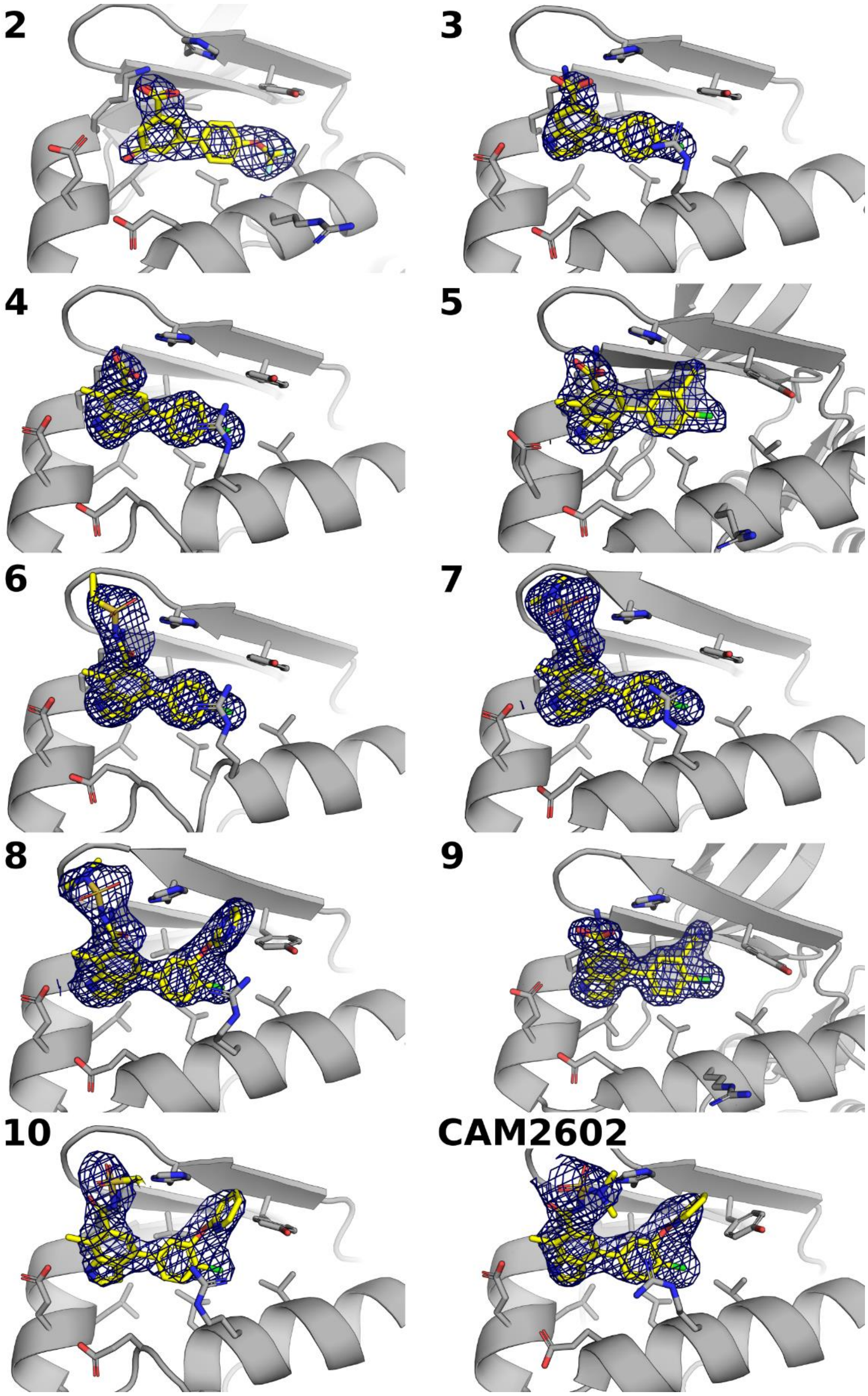

Figure S12. Electron densities of ligands

Electron densities of ligands **2-10** and **CAM2602** in complex with Aurora A after final refinement, all contoured at 1σ level.

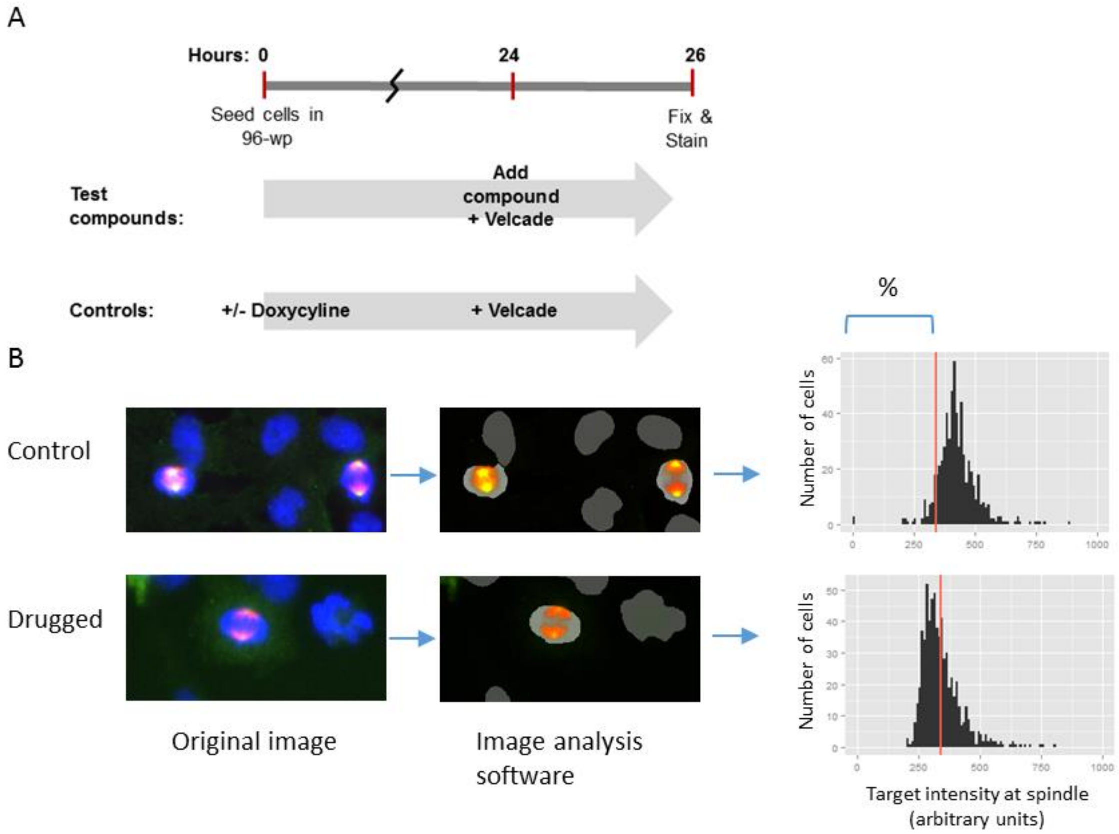

Figure S13. High-content assay to assess cellular Aurora A engagement by PPI inhibitors.
(A) Experimental structure for the HCS assay – timelines proceed from seeding cells to 96-well plates through to the final 2 hour treatment of the cells with compound titrations in the presence of Velcade. The control wells of each plate received either DMSO or doxycycline (for mCherry-TPX2-1–43 peptide induction) immediately following cell seeding, 24 hours prior to the drugging of the compound wells. All wells receive Velcade in the last 2 hours of the assay. (B) Example Aurora A mislocalisation assay images of vehicle control and compound treated HeLa cells after 2 hours of treatment. Left panels show merged-channel images of the fixed cells fluorescently stained for DNA (blue), TPX2 (red) and Aurora A (green). The middle panels show the Aurora A and TPX2 fluorescent stains superimposed onto the software-generated mask of nuclei positions generated from the DNA fluorescence data. Only the TPX2-positive, mitotic nuclei are retained for phenotypic assessment. The right panels show histograms of the two example cell populations where the x-axis shows the observed fluorescence intensity values for Aurora A immunostaining within the limits of the TPX2-immunostained mitotic spindle for every mitotic cell. The red line represents the assay threshold, calculated per plate, to the left of which cells exhibit loss of Aurora A from the spindle.

Chemical structures of key compounds

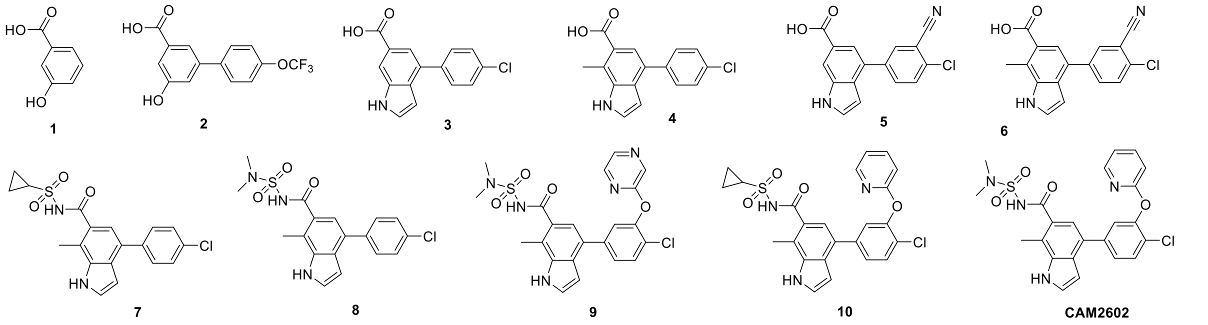

Figure S14 Chemical structures of key compounds

Synthetic Chemistry

Synthetic schemes

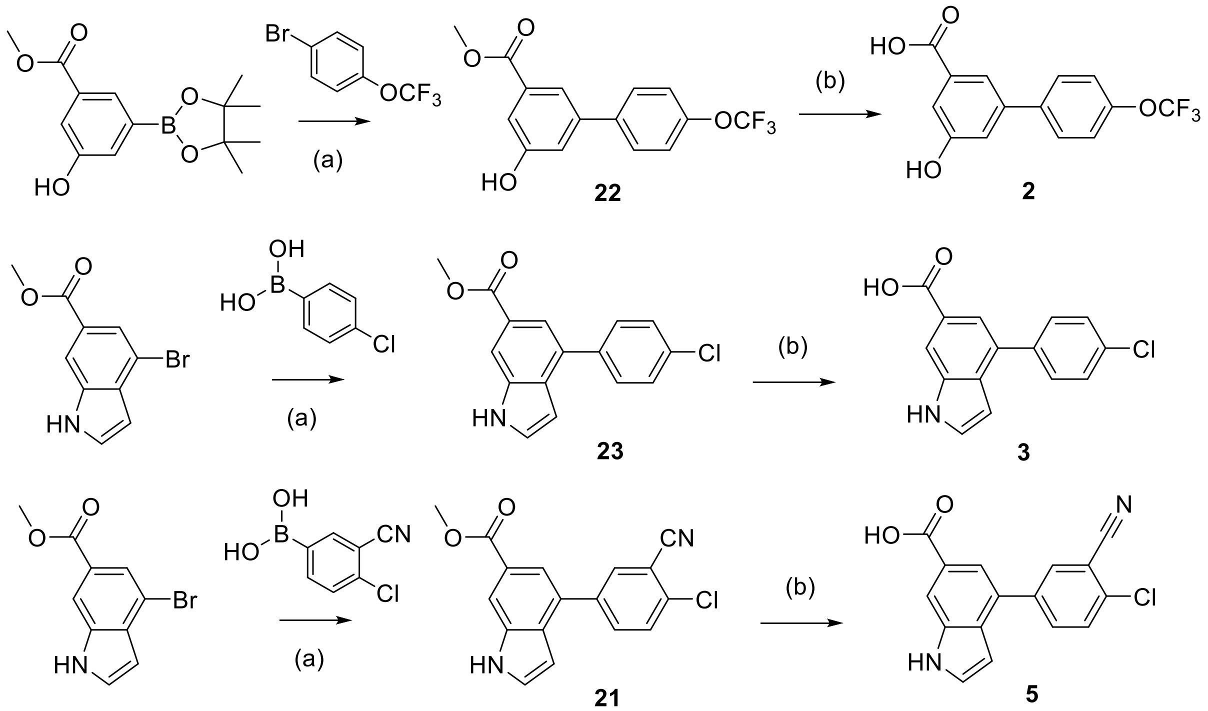

Scheme S1 Synthesis of compound 2, 3 and 5

(a) Pd(dppf)Cl_2 DCM, DME, H_2O, 120 °C (microwave), 0.5 h (b) NaOH, THF, H_2O, 45 °C.

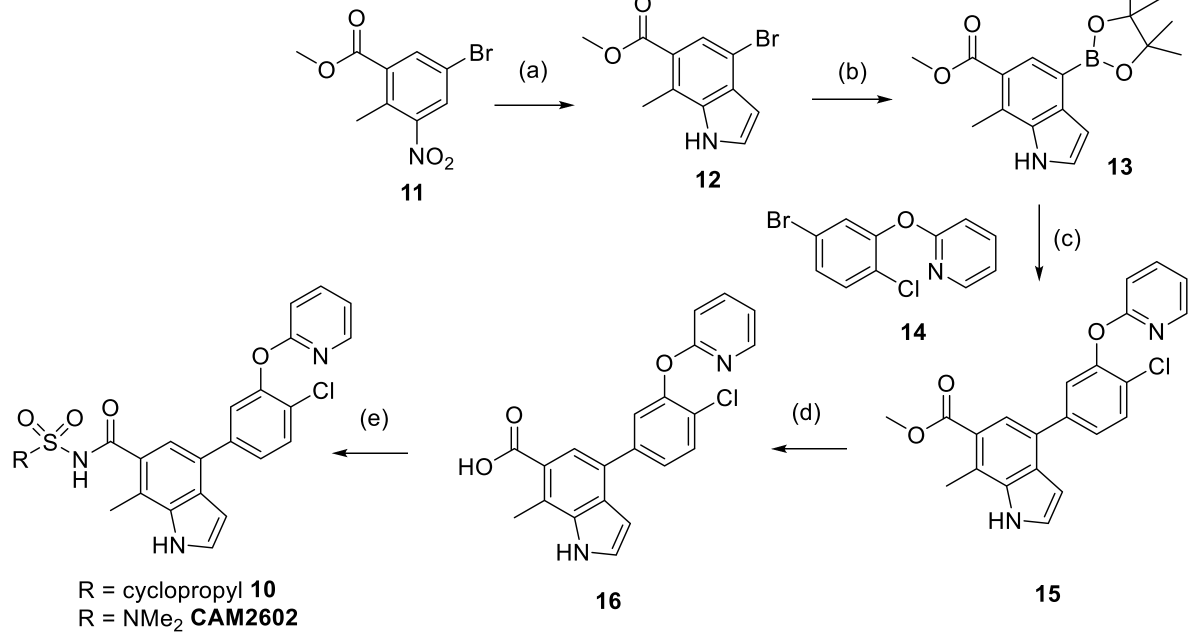

Scheme S2 Synthesis of compound 10 and CAM2602

(a) vinylmagnesium bromide, THF, -78 °C (b) bis(pinacolato)diboron, Pd(dppf)Cl_2·DCM, DMSO, 90 °C, 4 h (c) **14**, Pd(dppf)Cl_2 DCM, DME, H_2O, 120 °C (microwave), 0.5 h (d) LiOH, THF, water, 45 °C (e) (i) CDI, DBU, THF, 45 °C, 3h (ii) dimethylsulfamide, DBU, 80 °C.

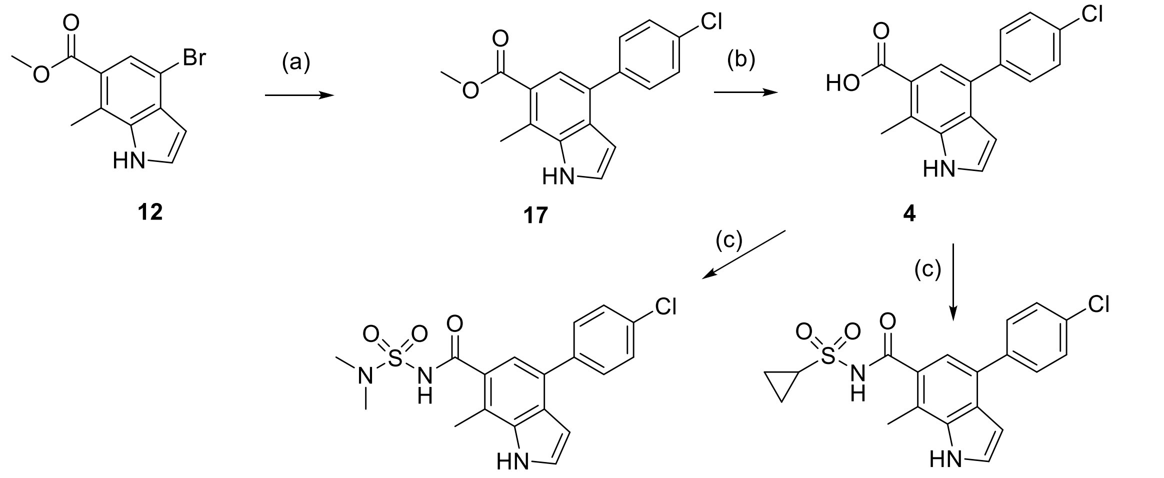

Scheme S3 Synthesis of compound 4, 7 and 8

(a) 4-Chlorophenylboronic acid, TEA, DME, water, Pd(dppf)Cl_2.CH_2Cl_2, 120 °C (microwave), 0.5 h (b) Lithium iodide, 180 °C (microwave), 1 h (c) (i) CDI, DBU, THF, 45 °C, 3h (ii) sulfonamide, DBU, 80 °C.

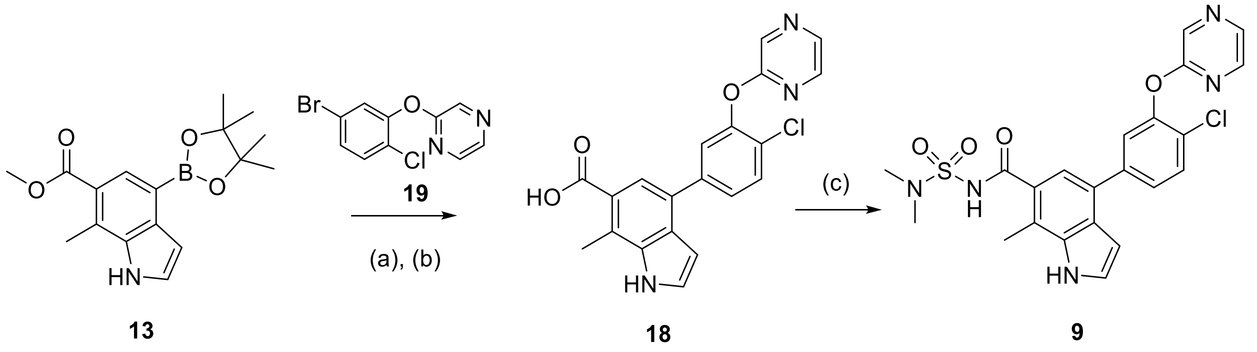

Scheme S4 Synthesis of compound 18 and 9

(a) **19**, TEA, DME, water, Pd(dppf)Cl_2·CH_2Cl_2, 120 °C (microwave), 0.5 h (b) LiOH, THF, water, 45 °C (c) (i) CDI, DBU, THF, 45 °C, 3h (ii) dimethylsulfamide, DBU, 80 °C.

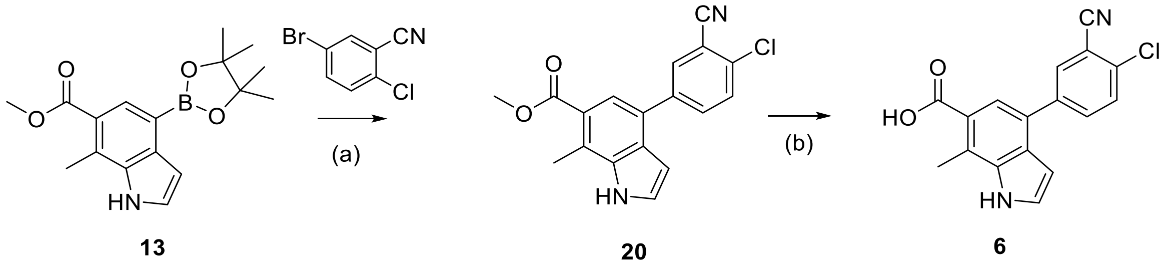

Scheme S5 Synthesis of compound 6

(a) 4-chloro-3-cyanophenylboronic acid, TEA, DME, water, Pd(dppf)Cl_2·CH_2Cl_2, 120 °C, 0.5 h (b) Lithium iodide, 180 °C (microwave), 1 h.

Final compound characterisation spectra

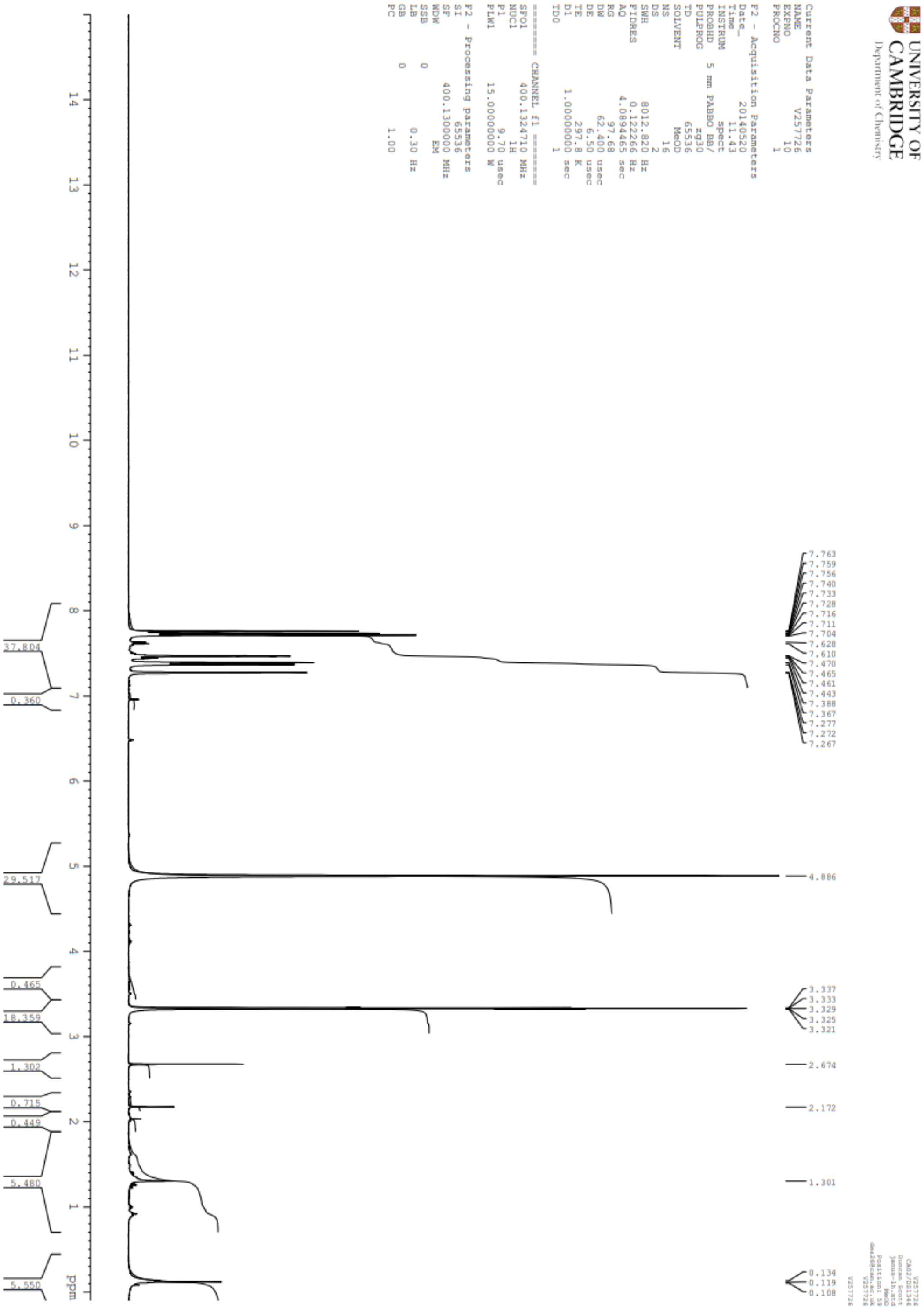

Figure S15 ^{1}H NMR of compound **2**

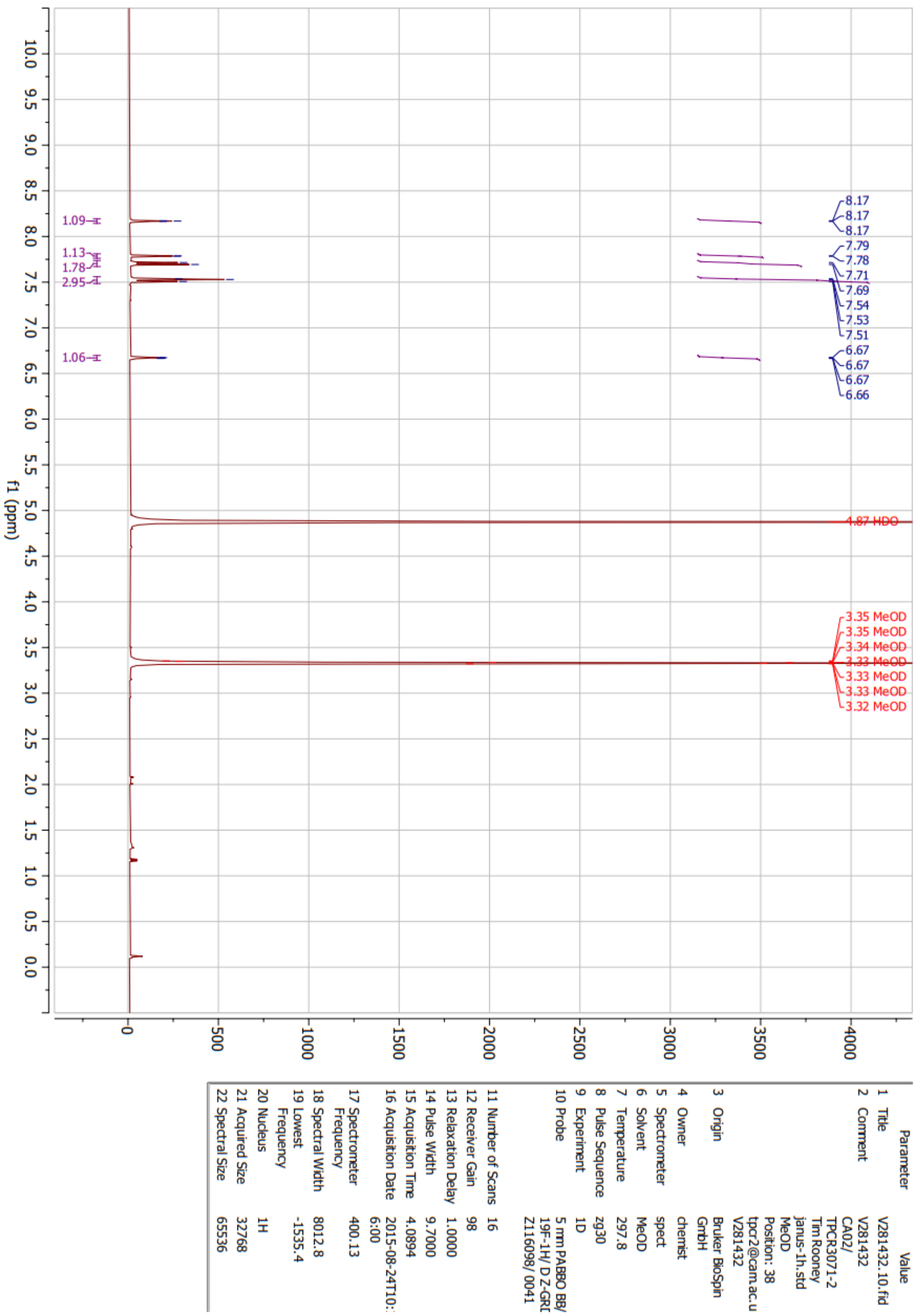

Figure S16 ^{1}H NMR of compound **3**

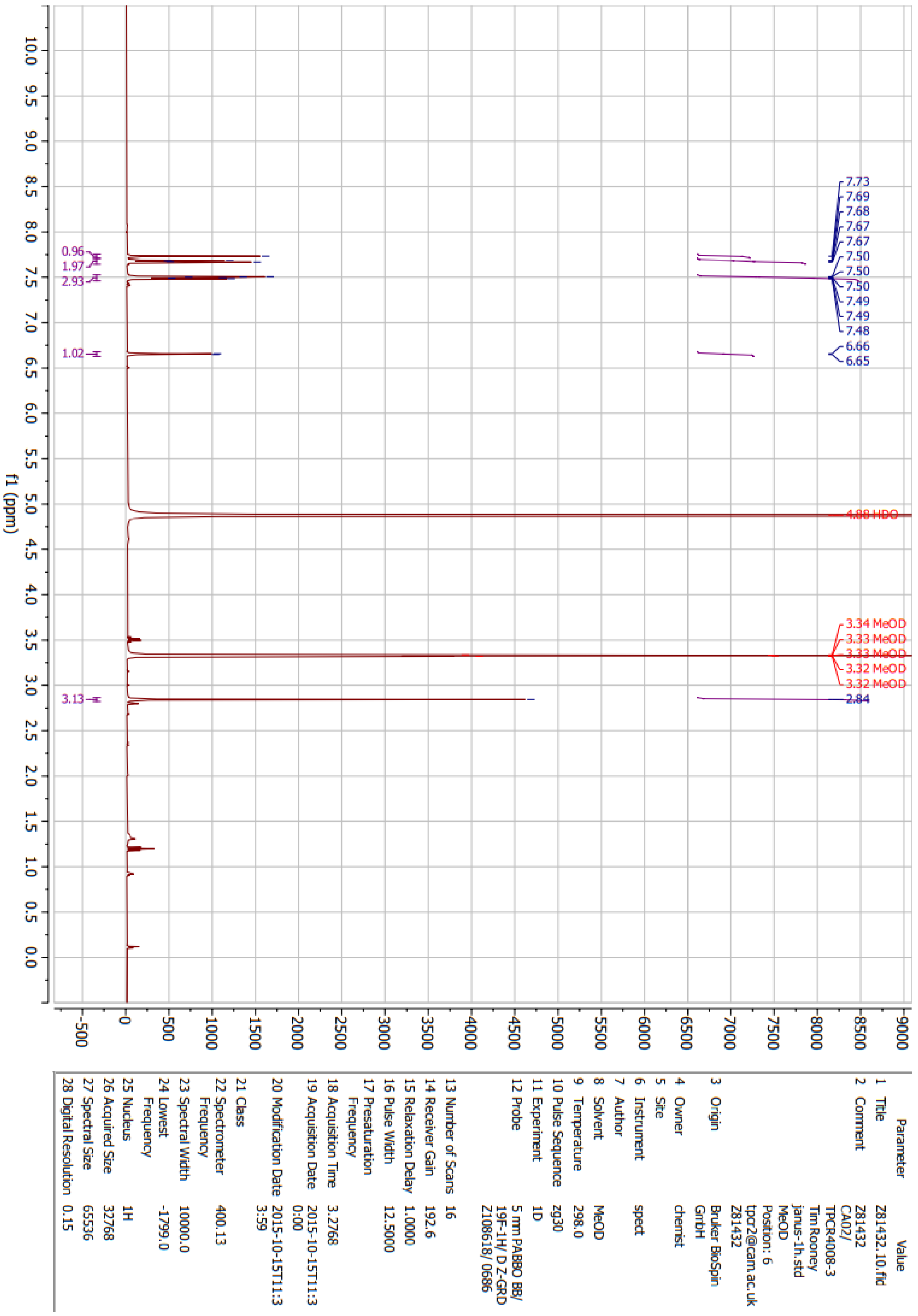

Figure S17 ^{1}H NMR of compound **4**

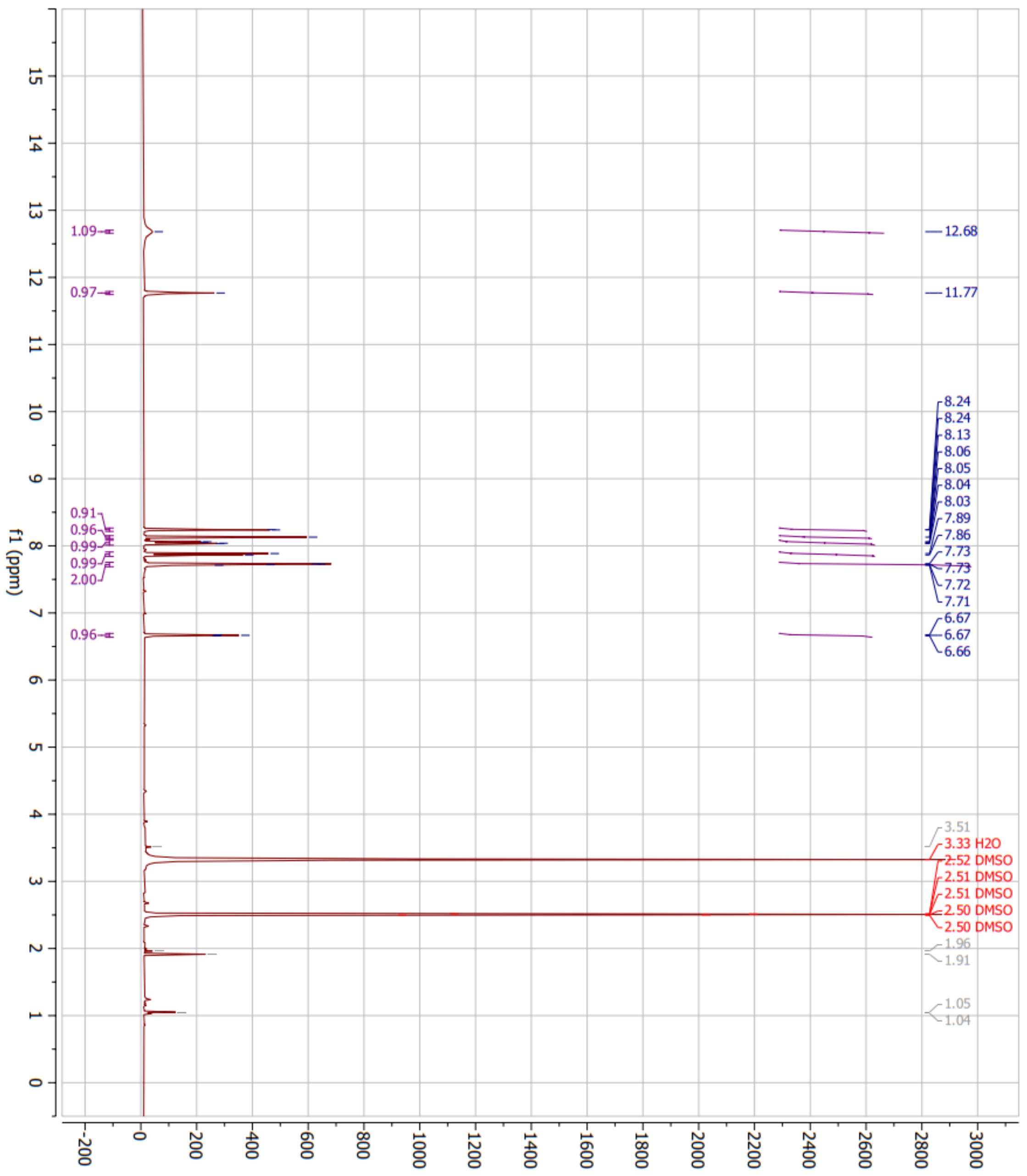

	Parameter	Value
1	Title	V262267.10.fid
2	Comment	V262267 CA02/ TPCR1033-3 Tim R janus-1h.std DMSO Position: 24 tpcr2@cam.ac.uk V262267
3	Origin	Bruker BioSpin GmbH
4	Owner	Administrator
5	Spectrometer	spect
6	Solvent	DMSO
7	Temperature	297.9
8	Pulse Sequence	zg30
9	Experiment	1D
10	Probe	5 mm PABBO BB/ 19F-1H/ D Z-GRD Z116098/ 0041
11	Number of Scans	16
12	Receiver Gain	98
13	Relaxation Delay	1.0000
14	Pulse Width	9.7000
15	Acquisition Time	4.0894
16	Acquisition Date	2014-08-01T15:22:00
17	Spectrometer Frequency	400.13
18	Spectral Width	8012.8
19	Lowest Frequency	-1535.4
20	Nucleus	1H
21	Acquired Size	32768
22	Spectral Size	65536

Figure S18 ^{1}H NMR of compound **5**

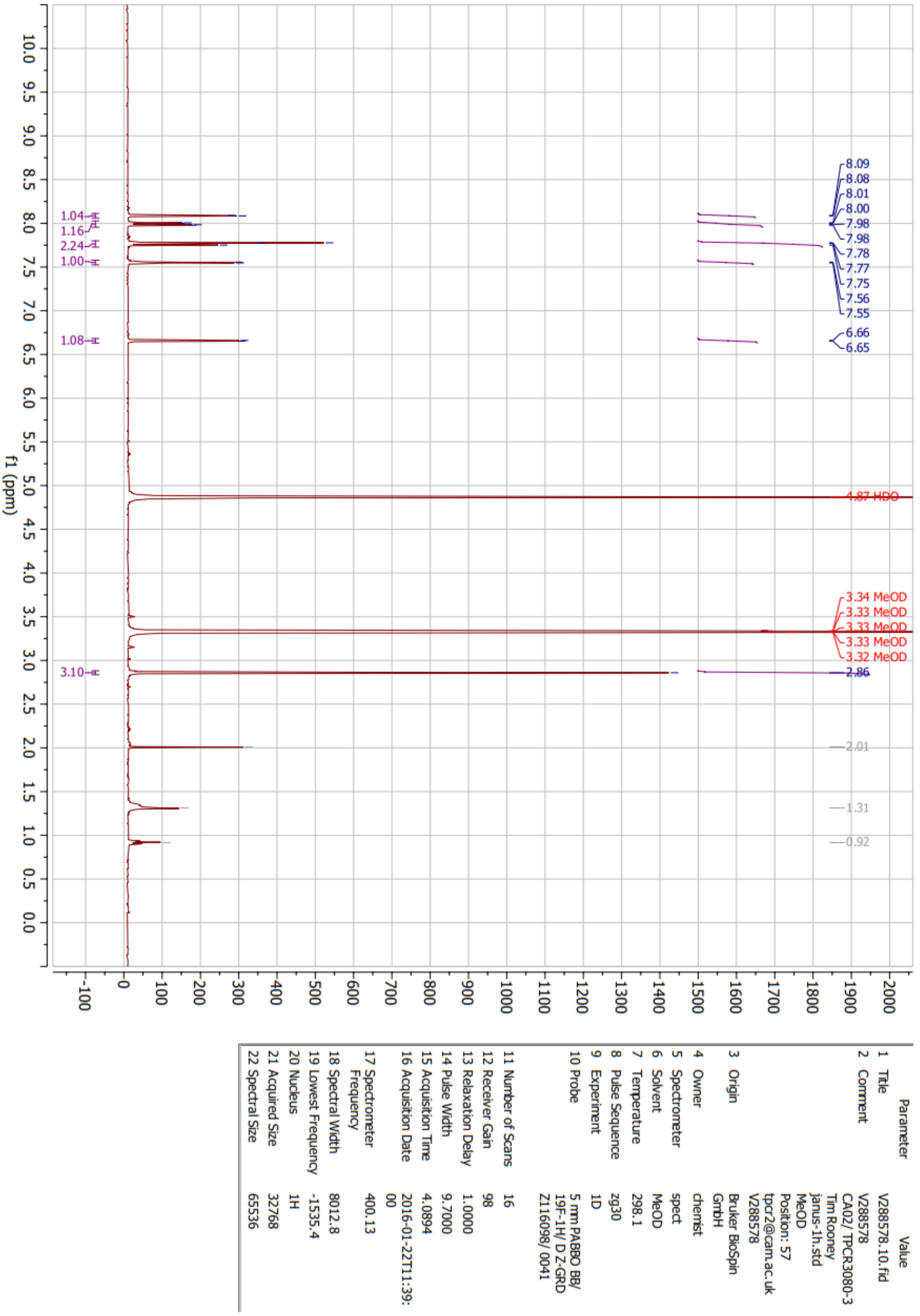

Figure S19 ^{1}H NMR of compound **6**

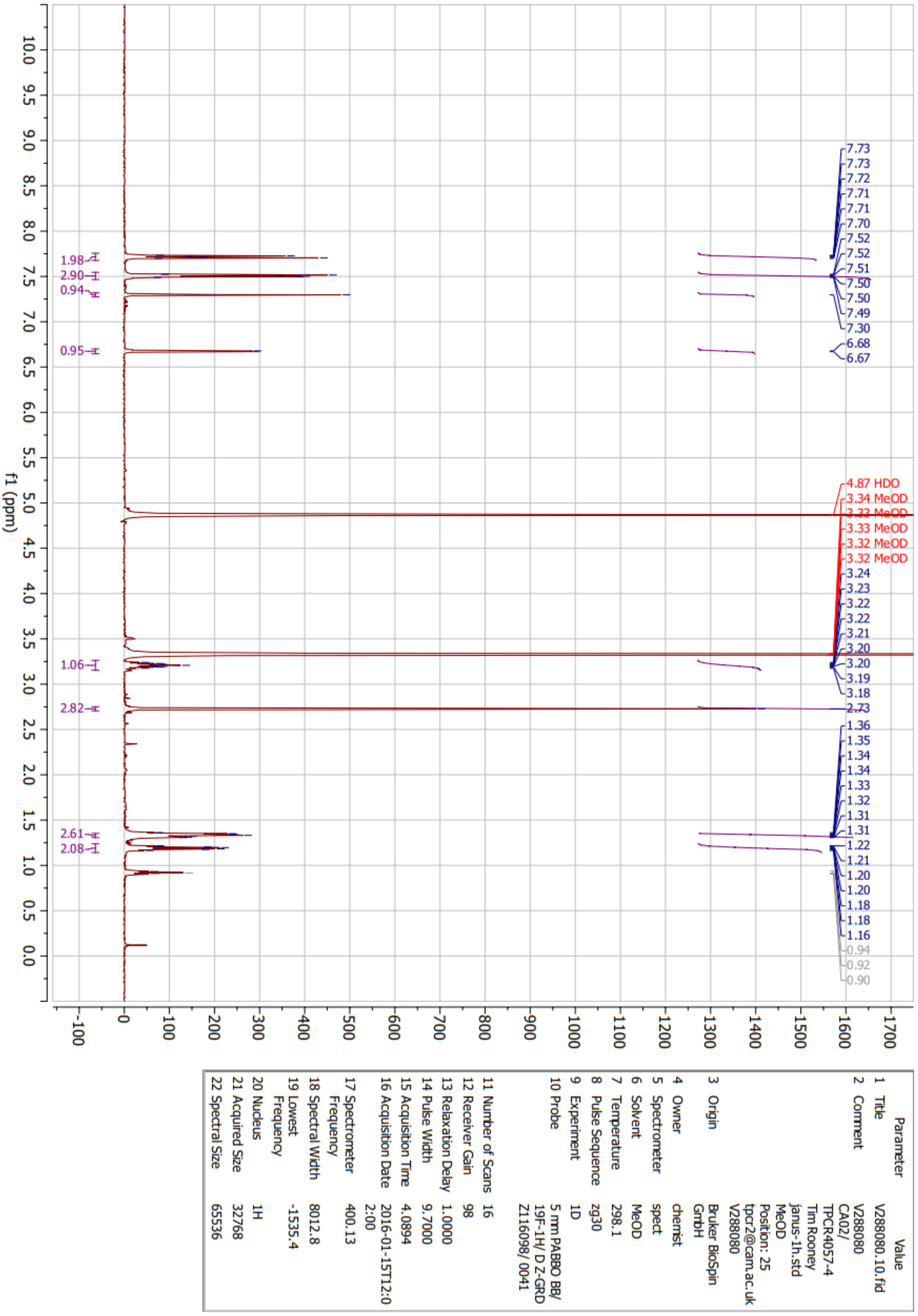

Figure S20 ^{1}H NMR of compound **7**

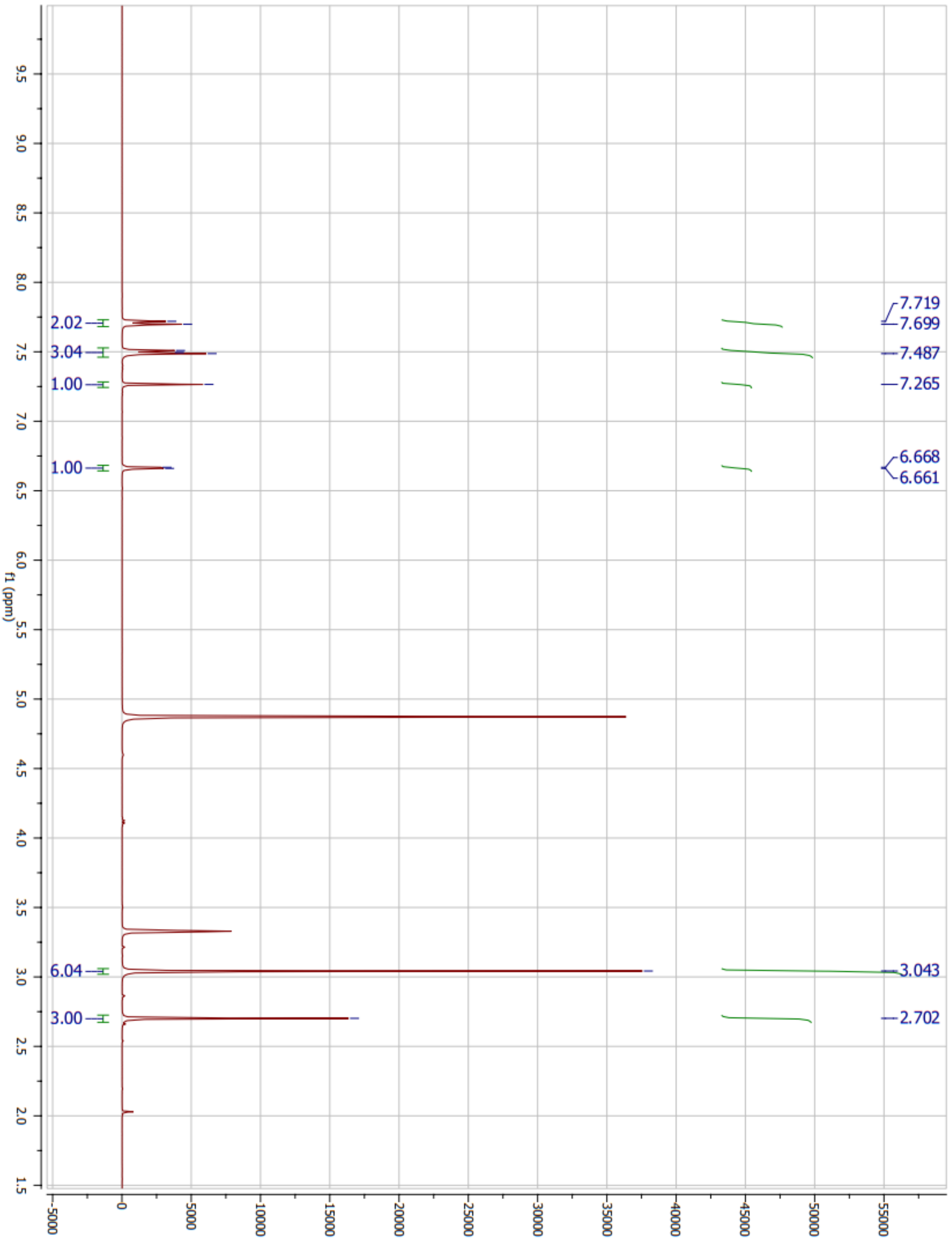

Figure S21 ^{1}H NMR of compound **8**

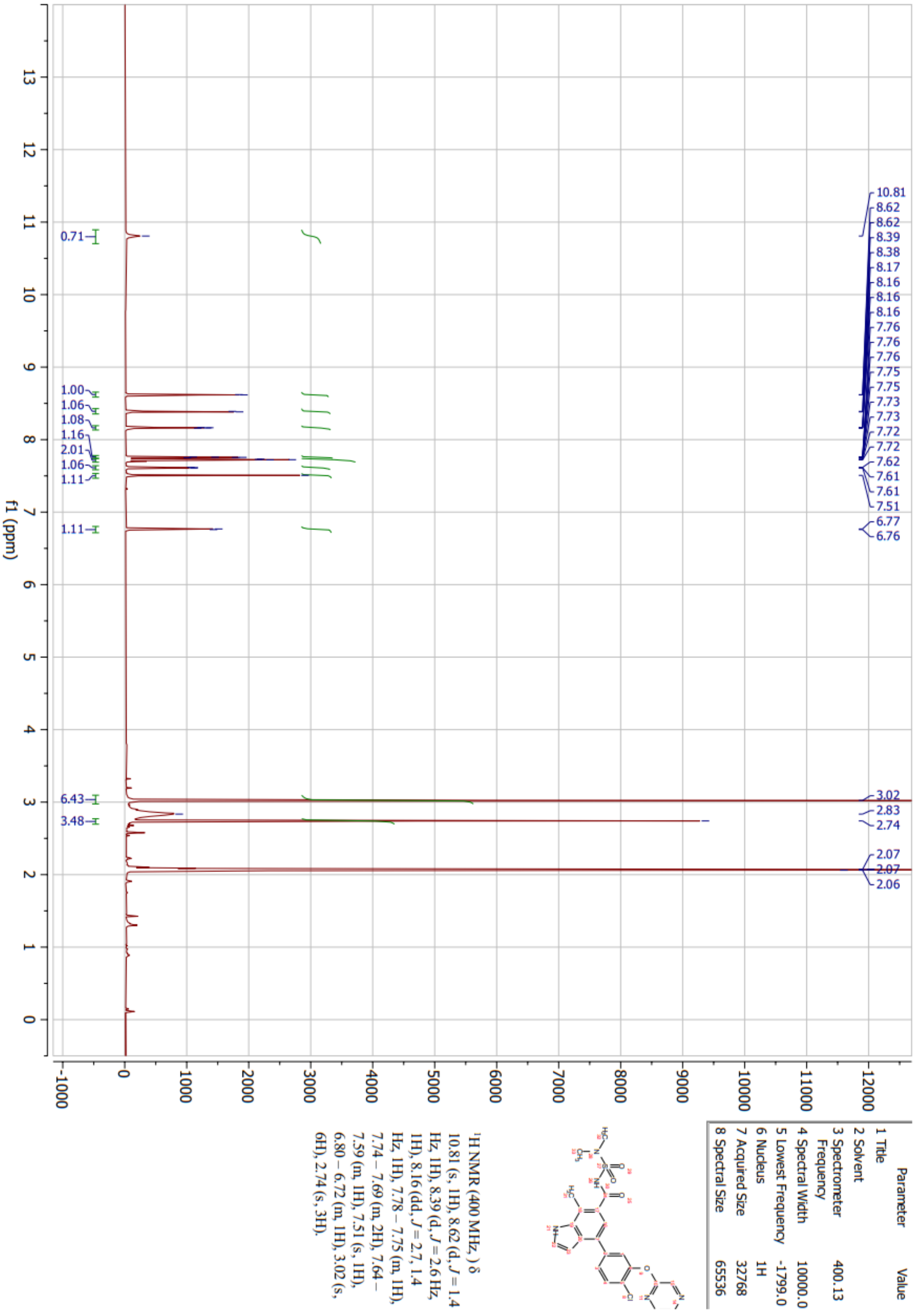

Figure S22 ^{1}H NMR of compound **9**

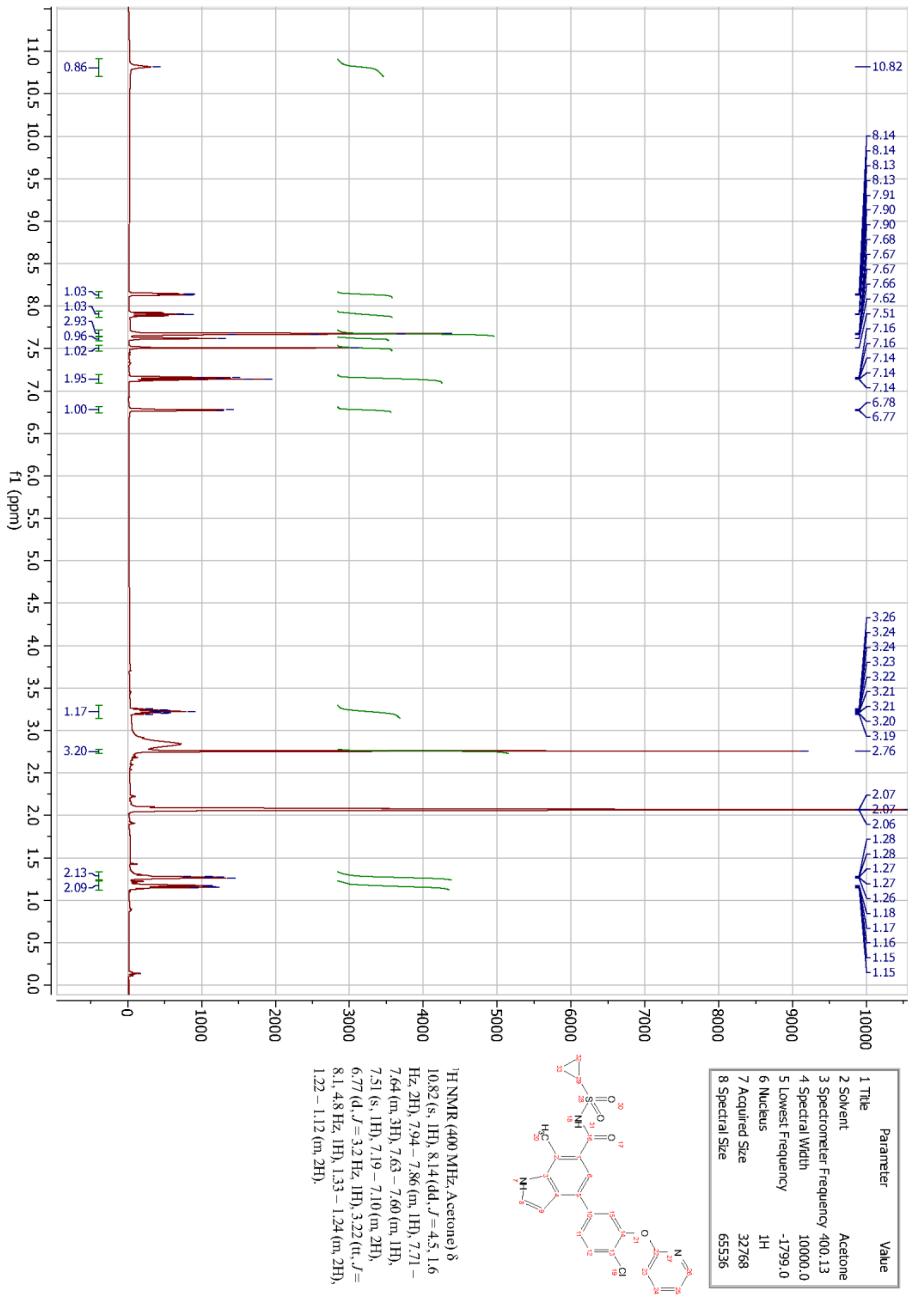

Figure S23 ^{1}H NMR of compound **10**

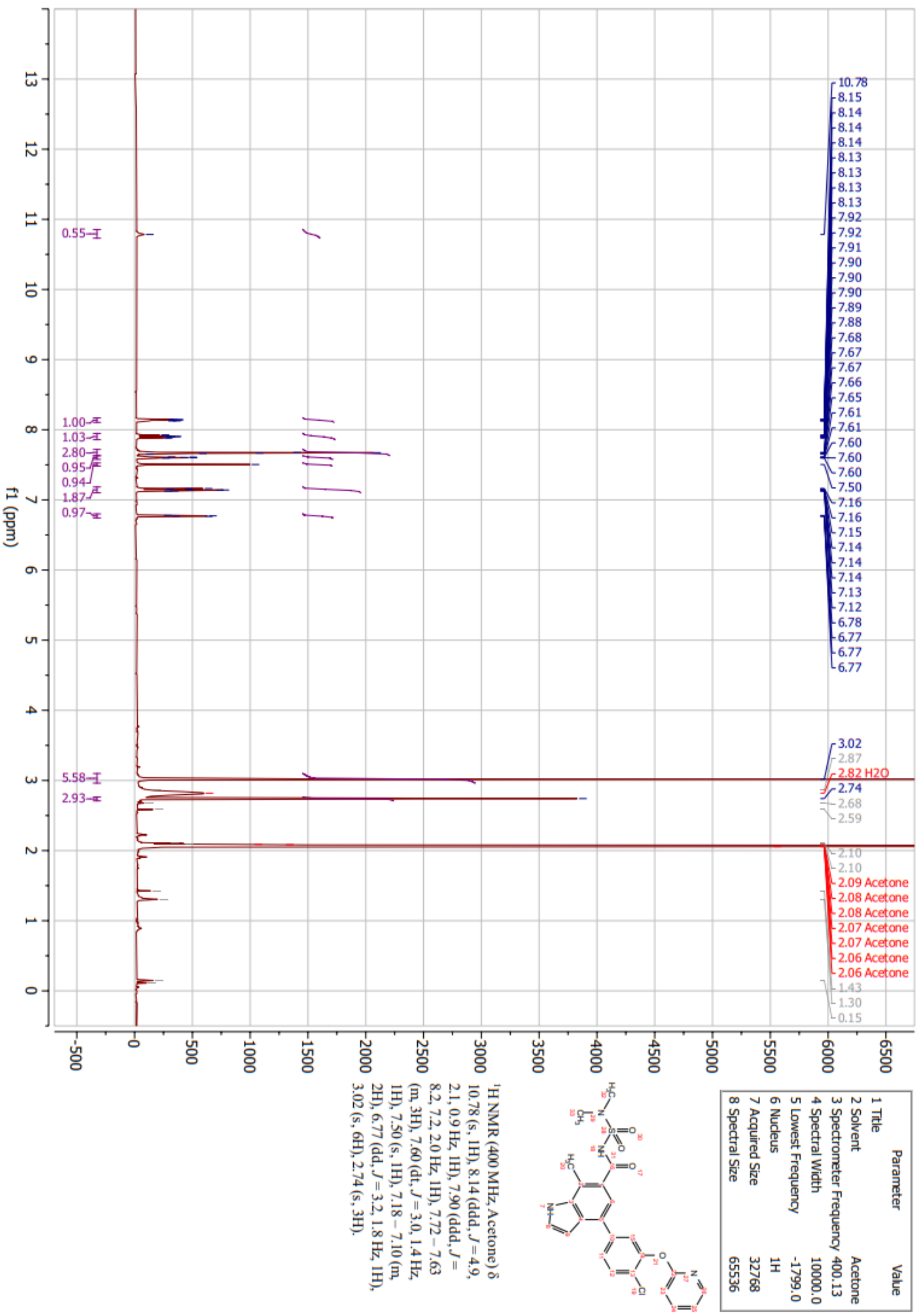

Figure S24 ^{1}H NMR of **CAM2602**

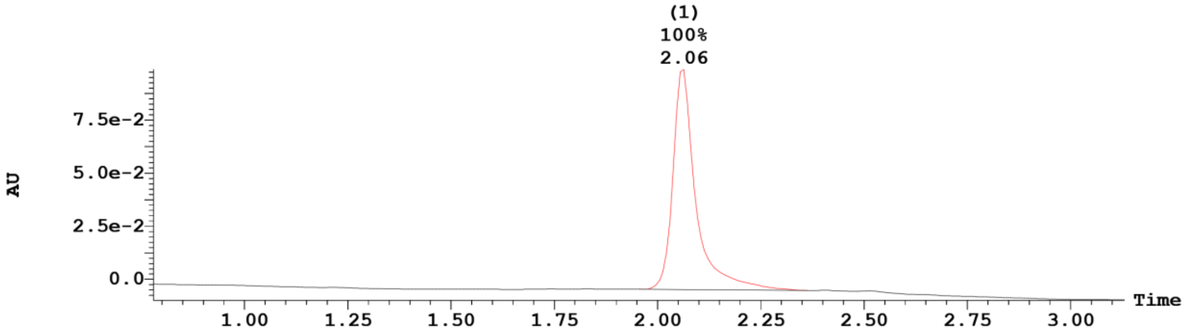

Peak Number	Time	AreaAbs	Area %Total	Height	Mass Found
1	2.06	6717	100.00	103629	

Figure S25. HPLC analysis of **2.**

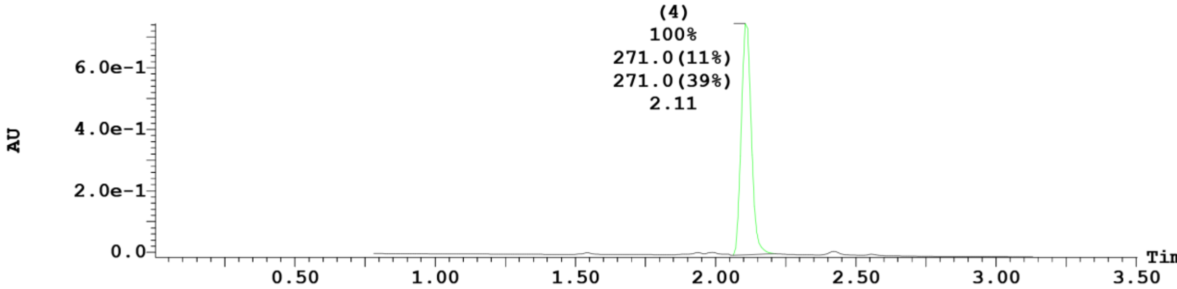

Peak Number	Time	AreaAbs	Area %Total	Height	Mass Found
4	2.11	31089	100.00	751327	271.00, 271.00

Figure S26. HPLC analysis of **3.**

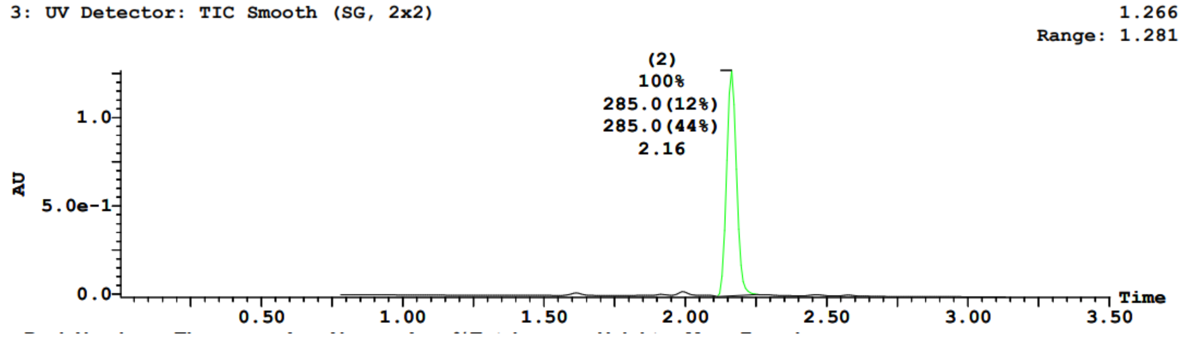

Figure S27. HPLC analysis of **4.**

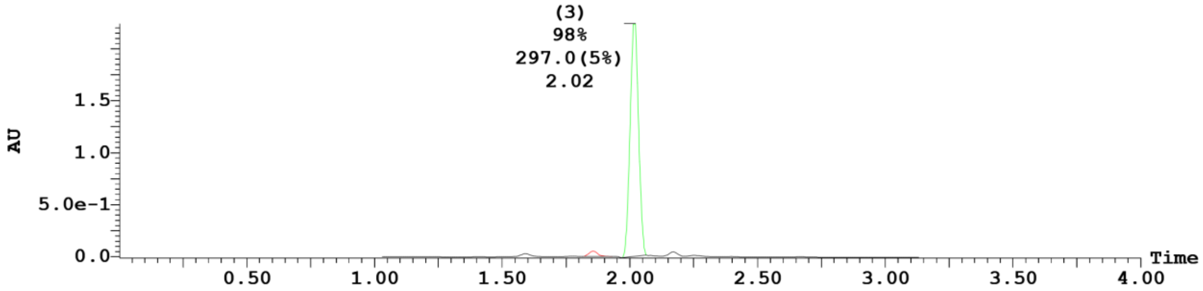

Peak Number	Compound	Time	AreaAbs	Area %Total	Width	Height	Mass Found
1		1.86	2e+003	2.37	0	5e+004	
3	Tentative	2.02	9e+004	97.63	0	2e+006	297.00

Figure S28. HPLC analysis of **5.**

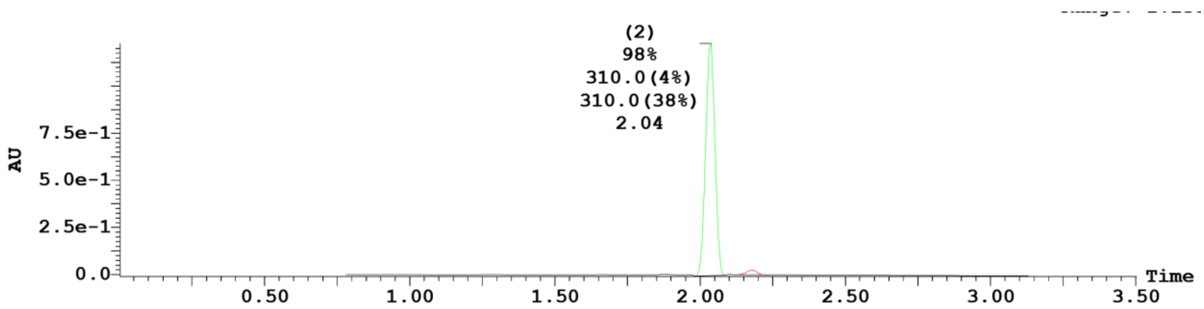

Peak Number	Time	AreaAbs	Area %Total	Height	Mass Found
2	2.04	46731	97.89	1230201	310.00, 310.00

Figure S29. HPLC analysis of **6.**

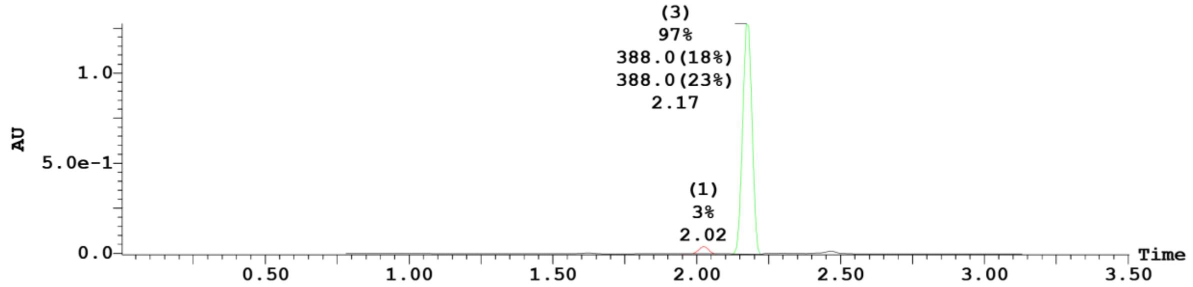

Peak Number	Time	AreaAbs	Area %Total	Height	Mass Found
1	2.02	1531	3.06	40077	
3	2.17	48551	96.94	1278146	388.00, 388.00

Figure S30. HPLC analysis of **7.**

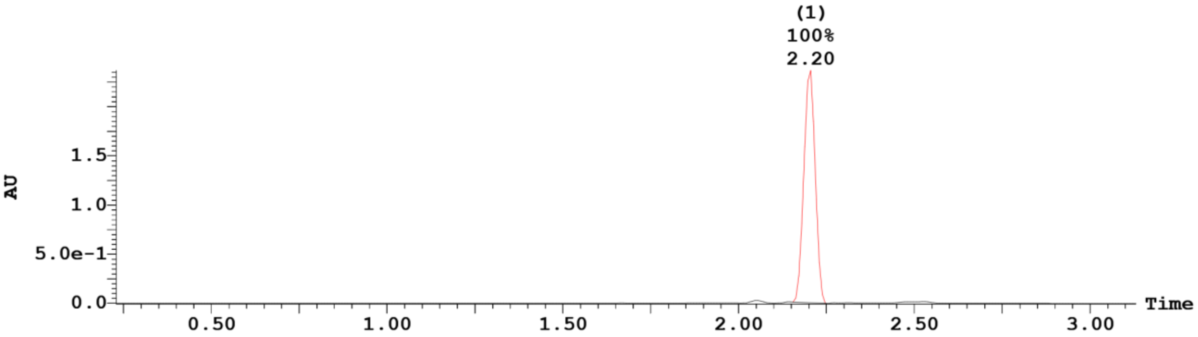

Peak Number	Time	AreaAbs	Area %Total	Height	Mass Found
1	2.20	89184	100.00	2358668	

Figure S31. HPLC analysis of **8.**

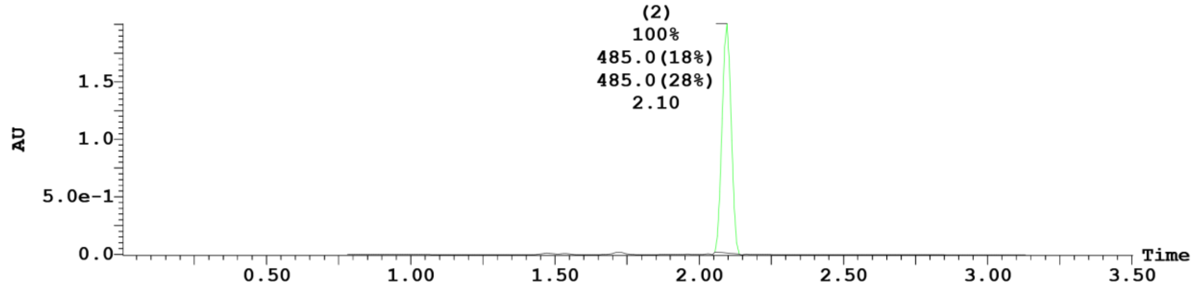

Peak Number	Time	AreaAbs	Area %Total	Height	Mass Found
2	2.10	72841	100.00	1993751	485.00, 485.00

Figure S32. HPLC analysis of **9.**

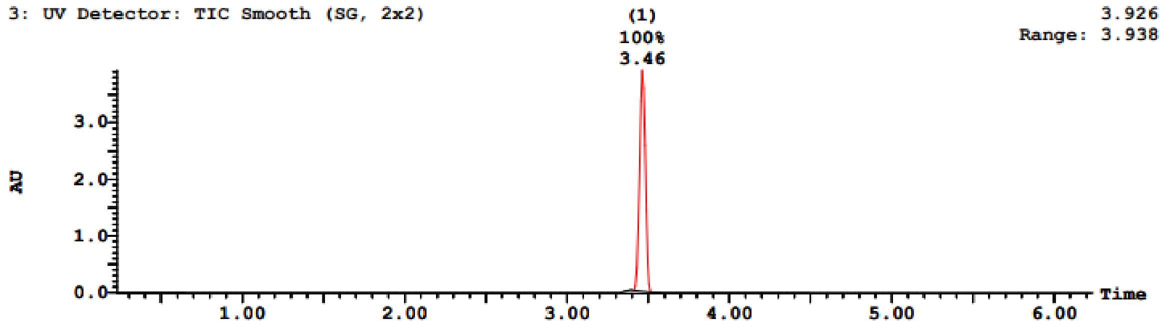

Peak Number	Time	AreaAbs	Area %Total	Height	Mass Found
1	3.46	162591	100.00	3906605	

Figure S33. HPLC analysis of **10.**

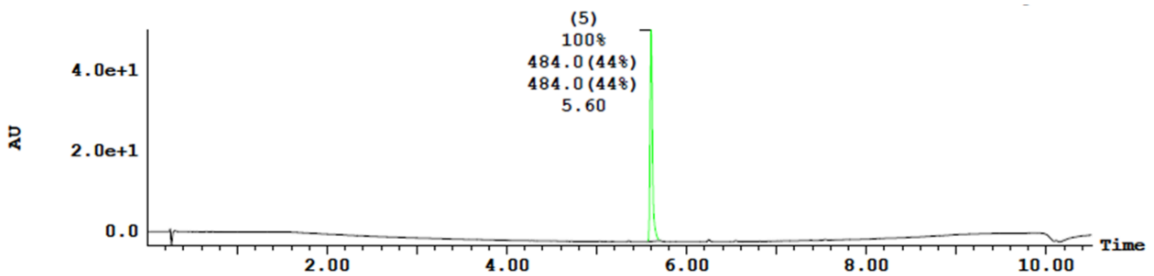

Peak Number	Compound	Time	Area %Total	Mass Found
5	Found	5.60	100.00	484.0000, 484.0000

Figure S34. HPLC analysis of **CAM2602.**
